# BCS Critical Temperature on Half-Spaces

**DOI:** 10.1007/s00205-025-02088-x

**Published:** 2025-03-02

**Authors:** Barbara Roos, Robert Seiringer

**Affiliations:** Institute of Science and Technology Austria, Am Campus 1, 3400 Klosterneuburg, Austria

## Abstract

We study the BCS critical temperature on half-spaces in dimensions $d=1,2,3$ with Dirichlet or Neumann boundary conditions. We prove that the critical temperature on a half-space is strictly higher than on $\mathbb {R}^d$, at least at weak coupling in $d=1,2$ and weak coupling and small chemical potential in $d=3$. Furthermore, we show that the relative shift in critical temperature vanishes in the weak coupling limit.

## Introduction and Results

We study the effect of a boundary on the critical temperature of a superconductor in Bardeen–Cooper–Schrieffer theory. It was recently observed [1, 2, 14–16] that the presence of a boundary may increase the critical temperature. For a one-dimensional system with $\delta$-interaction, a rigorous mathematical justification was given in [6]. Here, we generalize this result to generic interactions and higher dimensions. While in dimensions $d=2,3$ the existing numerical works only consider lattice models, our analytic approach allows us to study continuum models. We compare the half infinite superconductor with shape $\Omega _1=(0,\infty ) \times {\mathbb {R}}^{d-1}$ to the superconductor on $\Omega _0={\mathbb {R}}^d$ in dimensions $d=1,2,3$. We impose either Dirichlet or Neumann boundary conditions, and prove that in the presence of a boundary the critical temperature can increase. The critical temperature can be determined from the spectrum of the two-body operator

1.1 $$\begin{aligned} H_T^\Omega = \frac{-\Delta _x-\Delta _y-2\mu }{\tanh \left( \frac{-\Delta _x-\mu }{2T}\right) +\tanh \left( \frac{-\Delta _y-\mu }{2T}\right) }-\lambda V(x-y) \end{aligned}$$

acting in $L_\textrm{sym}^2(\Omega \times \Omega )=\{\psi \in L^2(\Omega \times \Omega ) \vert \psi (x,y)=\psi (y,x)\ \mathrm {for\ all}\ x,y\in \Omega \}$ with appropriate boundary conditions [3]. Here, $\Delta$ denotes the Dirichlet or Neumann Laplacian on $\Omega$ and the subscript indicates on which variable it acts. Furthermore, *T* denotes the temperature, $\mu$ is the chemical potential, *V* is the interaction and $\lambda$ is the coupling constant. The first term in $H_T^\Omega$ is defined through functional calculus.

Let us explain how $H_T^\Omega$ relates to the BCS critical temperature of a superconductor. A mathematical introduction to BCS theory can be found in [8]. BCS theory describes the state of the system as the minimizer of the BCS functional ${\mathcal {F}}$. The normal state $\Gamma _0$ is the minimizer of ${\mathcal {F}}$ among states which do not exhibit any superconductivity. If perturbations of $\Gamma _0$ that introduce pairing between electrons decrease the value of ${\mathcal {F}}$, the system is superconducting. It turns out that the normal state is always a critical point of ${\mathcal {F}}$ and therefore the behavior of ${\mathcal {F}}$ in the vicinity of $\Gamma _0$ is determined by the Hessian, which is exactly $2 H_T^\Omega$, as explained in [3]. Importantly, the normal state is unstable and the system is superconducting if $\inf \sigma (H_T^\Omega ) <0$. For translation invariant systems, i.e. $\Omega ={\mathbb {R}}^d$, with suitable interactions *V* superconductivity is equivalent to $\inf \sigma (H_T^\Omega ) <0$. This was shown in [5, 8] in the case without symmetry restriction on the Cooper pair wave function and can be adapted to the case with symmetry restriction, as explained in [12]. In this case, there is a unique critical temperature $T_c$ determined by $\inf \sigma (H_{T_c}^\Omega ) = 0$ which separates the superconducting and the normal phase. The critical temperatures $T_c^{\Omega _0}$ and $T_c^{\Omega _1}$ are defined as

1.2 $$\begin{aligned} T_c^{\Omega _j}(\lambda ):= \inf \{T\in (0,\infty ) \vert \inf \sigma (H_T^{\Omega _j})\ge 0\}. \end{aligned}$$

In [14] an equivalent definition of the critical temperature was used based on the Birman–Schwinger version of $H_T^\Omega$ and the Mittag-Leffler series for $\tanh$. In Lemma 2.3 we prove the inequality $\inf \sigma (H_T^{\Omega _1}) \le \inf \sigma (H_T^{\Omega _0})$. Therefore, $T_c^{\Omega _1}(\lambda )\ge T_c^{\Omega _0}(\lambda )$. Our main concern is to show that the inequality is strict, which means that there is a temperature range for which the system with boundary is superconducting while the system on ${\mathbb {R}}^d$ is not.

Our strategy involves proving $\inf \sigma (H_{T}^{\Omega _1})<0$ for $T=T_c^{\Omega _0}(\lambda )$ using the variational principle. The idea is to construct a trial state involving the ground state of $H_{T}^{\Omega _0}$ at temperature $T=T_c^{\Omega _0}(\lambda )$. However, $H_T^{\Omega _0}$ is translation invariant in the center of mass coordinate and thus has purely essential spectrum. To obtain a ground state eigenfunction, we remove the translation invariant directions, and instead consider the reduced operator

1.3 $$\begin{aligned} H_T^0=\frac{-\Delta -\mu }{\tanh \left( \frac{-\Delta -\mu }{2T}\right) }-\lambda V(r) \end{aligned}$$

acting in $L^2({\mathbb {R}}^d)$, which corresponds to zero total momentum in $H_T^{\Omega _0}$. At weak enough coupling, the infimum of $\sigma (H_{T}^{\Omega _0})$ for $T=T_c^{\Omega _0}(\lambda )$ is attained at zero total momentum (c.f. Lemma 2.4 and Remark 2.5). Our trial state involves the ground state of $H_{T}^0$ at temperature $T=T_c^{\Omega _0}(\lambda )$. In the weak coupling limit, $\lambda \rightarrow 0$, we can compute the asymptotic form of this ground state provided that $\mu >0$ and the operator $\mathcal {V}_\mu :L^2({\mathbb {S}}^{d-1})\rightarrow L^2({\mathbb {S}}^{d-1})$ with integral kernel

1.4 $$\begin{aligned} \mathcal {V}_\mu (p,q)=\frac{1}{(2\pi )^{d/2}}\widehat{V}(\sqrt{\mu }(p-q)) \end{aligned}$$

has a non-degenerate eigenvalue $e_\mu = \sup \sigma (\mathcal {V_\mu })>0$ at the top of its spectrum and the corresponding eigenfunction is even [8, 9]. Here, $\widehat{V}(p)=\frac{1}{(2\pi )^{d/2}}\int _{{\mathbb {R}}^d} V(r) e^{-i p \cdot r} {\textrm{d}}r$ denotes the Fourier transform of *V*. For $d=1$, $L^2({\mathbb {S}}^0)$ is a two-dimensional vector space, and $\mathcal {V}_\mu$ has the eigenvalues $\frac{\widehat{V}(0)\pm \widehat{V}(2\sqrt{\mu })}{(2\pi )^{1/2}}$, where the plus and minus sign correspond to an even and odd eigenfunction, respectively.

We make the following assumptions on the interaction potential:

### Assumption 1.1

Let $d\in \{1,2,3\}$ and $\mu >0$. Assume that

(i) $V\in L^1({\mathbb {R}}^d) \cap L^{p_d}({\mathbb {R}}^d)$, where $p_d=1$ for $d=1$, and $p_d>d/2$ for $d\in \{2,3\}$,
(ii) *V* is radial, $V\not \equiv 0$,
(iii) $$\vert \cdot \vert V \in L^1({\mathbb {R}}^d)$$,
(iv) $$\widehat{V}(0)>0$$,
(v) $e_\mu =\sup \sigma (\mathcal {V}_\mu )$ is a non-degenerate eigenvalue and the corresponding eigenfunction is even.

### Remark 1.2

The assumption $V\in L^1({\mathbb {R}}^d)$ implies that ${\widehat{V}}$ is continuous and bounded. The operator $\mathcal {V_\mu }$ is thus Hilbert–Schmidt and in particular compact. Due to Assumption (v) we have $e_\mu >0$. This in turn implies that the critical temperature $T_c^{\Omega _0}(\lambda )$ for the system on ${\mathbb {R}}^d$ is positive for all $\lambda >0$ (cf. Remark 2.5). Furthermore, for $d\ge 2$ radiality of *V* and (v) imply that the eigenfunction corresponding to $e_\mu$ must be rotation invariant, i.e. the constant function. Assumption (v) is satisfied for $d=2,3$ if $\widehat{V}\ge 0$ [8] and for $d=1$ if $\widehat{V}(0),\widehat{V}(2\sqrt{\mu })>0$.

These assumptions suffice to observe boundary superconductivity in $d=1,2$. For $d=3$, we need one additional condition. Let

1.5 $$\begin{aligned} j_d(r;\mu ):=\frac{1}{(2\pi )^{d/2}}\int _{{\mathbb {S}}^{d-1}} e^{i \omega \cdot r \sqrt{\mu }} {\textrm{d}}\omega . \end{aligned}$$

Define

1.6 $$\begin{aligned} {\widetilde{m}}_3^{D/N}(r;\mu )&:{=}&\int _{{\mathbb {R}}} \left( j_3(z_1,r_2,r_3;\mu )^2 {-} \vert j_3(z_1,r_2,r_3;\mu )\! \mp \! j_3(r;\mu ) \vert ^2 \chi _{|z_1|{<} |r_1|}\right) {\textrm{d}}z_1 \nonumber \\  &   \mp \frac{\pi }{\mu ^{1/2}} j_3(r;\mu )^2, \end{aligned}$$

where the indices *D* and *N* as well as the upper/lower signs correspond to Dirichlet/Neumann boundary conditions, respectively. Our main result is as follows:

### Theorem 1.3

Let $d\in \{1,2,3\}$, $\mu >0$ and let *V* satisfy Assumption 1.1. Assume either Dirichlet or Neumann boundary conditions. For $d=3$, additionally, assume that

1.7 $$\begin{aligned} \int _{{\mathbb {R}}^3} V(r) {\widetilde{m}}_3^{D/N}(r;\mu ) {\textrm{d}}r>0. \end{aligned}$$

Then there is a $\lambda _1>0$, such that for all $0<\lambda <\lambda _1$, $T_c^{\Omega _1}(\lambda )>T_c^{\Omega _0}(\lambda )$.

For $d=3$ we prove that (1.7) is satisfied for small enough chemical potential.

### Theorem 1.4

Let $d=3$ and let *V* satisfy 1.1 (i)–(iv). For Dirichlet boundary conditions, additionally assume that $\vert \cdot \vert ^2 V \in L^1({\mathbb {R}}^3)$ and $\int _{{\mathbb {R}}^3} V(r) |r|^2 {\textrm{d}}r >0$. Then there is a $\mu _0>0$ such that for all $0<\mu <\mu _0$, $\int _{{\mathbb {R}}^3} V(r) {\widetilde{m}}_3^{D/N}(r;\mu ) {\textrm{d}}r>0$. In particular, if *V* additionally satisfies 1.1 (v) for small $\mu$ (e.g. if ${\widehat{V}}\ge 0$), then for small $\mu$ there is a $\lambda _1(\mu )>0$ such that $T_c^{\Omega _1}(\lambda )>T_c^{\Omega _0}(\lambda )$ for $0<\lambda <\lambda _1(\mu )$.

### Remark 1.5

**Fig. 1:**
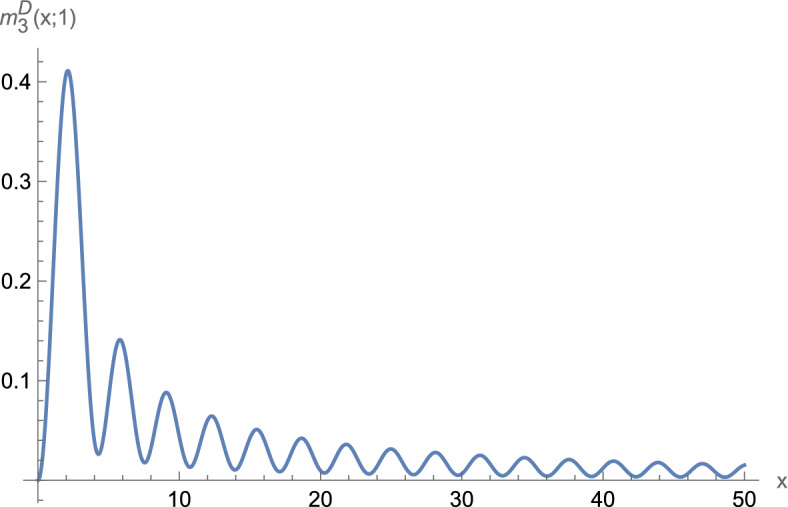
Plot of $m_3^D$ for $\mu =1$, created using [17]

Numerical evaluation of ${\widetilde{m}}_3^D$ suggests that ${\widetilde{m}}_3^D\ge 0$ (see Sect. 5, in particular Fig. 1). Hence, for Dirichlet boundary conditions (1.7) appears to hold under the additional assumption that $V\ge 0$. We therefore expect that for Dirichlet boundary conditions also in three dimensions boundary superconductivity occurs for all values of $\mu$. There is no proof so far, however.

### Remark 1.6

One may wonder why in $d=1,2$ no condition like (1.7) is needed. Actually, in $d=1,2$ the analogous condition is always satisfied if $\widehat{V}(0)>0$. The reason is that if one defines ${\widetilde{m}}_d^{D/N}(r;\mu )$ by replacing $j_3$ by $j_d$ in (1.6), the first term diverges and ${\widetilde{m}}_d^{D/N}(r;\mu )=+\infty$.

Our second main result is that the relative shift in critical temperature vanishes as $\lambda \rightarrow 0$. This generalizes the corresponding result for $d=1$ with contact interaction in [6].

### Theorem 1.7

Let $d\in \{1,2,3\}$, $\mu >0$ and let *V* satisfy Assumption 1.1 and $V\ge 0$. Then

1.8 $$\begin{aligned} \lim _{\lambda \rightarrow 0} \frac{T_c^{\Omega _1}(\lambda )-T_c^{\Omega _0}(\lambda )}{T_c^{\Omega _0}(\lambda )}=0. \end{aligned}$$

We expect that the additional assumption $V\ge 0$ in Theorem 1.7 is not necessary; it is required in our proof, however.

### Remark 1.8

The temperature $T_c^{\Omega _1}(\lambda )$ is the smallest temperature *T* satisfying $\inf \sigma (H_T^{\Omega _1})=0$. In principle, there could be other solutions to this equation, defining larger critical temperatures. An inspection of our proof shows that it applies equally well to these larger temperatures, i.e. Theorem 1.7 also holds if $T_c^{\Omega _1}(\lambda )$ is replaced by any other solution *T* of the equation $\inf \sigma (H_T^{\Omega _1})=0$.

The rest of the paper is organized as follows. In Sect. 2 we prove the Lemmas mentioned in the introduction. In Sect. 3 we use the Birman–Schwinger principle to study the ground state of $H_{T}^0$. Section 4 contains the proof of Theorem 1.3. Section 5 discusses the conditions under which (1.7) holds and in particular contains the proof Theorem 1.4. In Sect. 6 we study the relative temperature shift and prove Theorem 1.7. Section 7 contains the proof of auxiliary Lemmas from Sect. 6.

## Preliminaries

The following functions will occur frequently:

2.1 $$\begin{aligned} K_{T,\mu }(p,q):=\frac{p^2 + q^2 - 2\mu }{\tanh \left( \frac{p^2-\mu }{2T}\right) +\tanh \left( \frac{q^2-\mu }{2T}\right) } \end{aligned}$$

and

2.2 $$\begin{aligned} B_{T,\mu }(p,q):=\frac{1}{K_{T,\mu }(p+q,p-q)}. \end{aligned}$$

We will suppress the subscript $\mu$ and write $K_{T},B_T$ when the $\mu$-dependence is not relevant. The following estimate [6, Lemma 2.1] will prove useful:

### Lemma 2.1

For every $T_0>0$ there is a constant $C_1(T_0,\mu )>0$ such that for $T>T_0$, $C_1(T+p^2+q^2)\le K_{T}(p,q)$. For every $T>0$ there is a constant $C_2(T,\mu )>0$ such that $K_{T}(p,q)\le C_2(p^2+q^2+1)$.

The minimal value of $K_T$ is 2*T*. Since $|\tanh (x)|<1$, we have for all $p,q\in {\mathbb {R}}^d$ and $T\ge 0$

2.3 $$\begin{aligned}  &   B_T(p,q) \le \frac{1}{\max \{|p^2+q^2-\mu |,2T\}} \quad \textrm{and} \nonumber \\  &   B_T(p,q)\chi _{p^2+q^2>2\mu >0} \le \frac{C(\mu )}{1+p^2+q^2}, \end{aligned}$$

where $C(\mu )$ depends only on $\mu$.

### Remark 2.2

Assumption 1.1(i) guarantees that *V* is infinitesimally form bounded with respect to $-\Delta _x-\Delta _y$ [11, 13]. By Lemma 2.1, $H_T^\Omega$ defines a self-adjoint operator via the KLMN theorem. Furthermore, $H_T^\Omega$ becomes positive for *T* large enough and hence the critical temperatures are finite.

Let $K_{T}^\Omega$ be the kinetic term in $H_T^\Omega$. The corresponding quadratic form acts as $\langle \psi , K_{T}^\Omega \psi \rangle = \int _{\Omega ^4} \overline{\psi (x,y)}K_{T}^\Omega (x,y;x',y') \psi (x',y') {\textrm{d}}x {\textrm{d}}y{\textrm{d}}x'{\textrm{d}}y'$ where $K_{T}^\Omega (x,y; x',y')$ is the distribution

2.4 $$\begin{aligned} K_{T}^\Omega (x,y;x',y')=\int _{{\mathbb {R}}^{2d}}\overline{F_\Omega (x,p) F_\Omega (y,q)}K_{T}(p,q) F_\Omega (x',p) F_\Omega (y',q){\textrm{d}}p{\textrm{d}}q, \nonumber \\ \end{aligned}$$

with

2.5 $$\begin{aligned} F_{{\mathbb {R}}^d}(x,p)=\frac{e^{-i p \cdot x}}{(2\pi )^{d/2}} \quad \textrm{and} \quad F_{\Omega _1}(x,p)=\frac{(e^{-i p_1 x_1}\mp e^{i p_1 x_1}) e^{-i {\tilde{p}} \cdot {\tilde{x}}}}{2^{1/2}(2\pi )^{d/2}}, \nonumber \\ \end{aligned}$$

where the $-/+$ sign corresponds to Dirichlet and Neumann boundary conditions, respectively. Here, ${\tilde{x}}$ denotes the vector containing all but the first component of *x*. (In the case $d=1$, ${\tilde{x}}$ is empty and can be omitted.)

### Lemma 2.3

Let $T,\lambda >0$, $d\in \{1,2,3\}$, and let *V* satisfy 1.1 (i). Then $\inf \sigma (H_T^{\Omega _1})\le \inf \sigma (H_T^{\Omega _0})$.

With the with Lemma we may use $H_T^0$ instead of $H_T^{\Omega _0}$ to compute $T_c^{\Omega _0}(\lambda )$ at weak enough coupling.

### Lemma 2.4

Let $T,\lambda >0$, $d\in \{1,2,3\}$, and let *V* satisfy 1.1(i). Let $\sigma _{\textrm{s}}(H_T^{0})$ denote the spectrum of $H_T^0$ restricted to even functions. Then $\inf \sigma (H_T^0)\le \inf \sigma (H_T^{\Omega _0}) \le \inf \sigma _{\textrm{s}}(H_T^0)$.

### Remark 2.5

Under Assumption 1.1, for all couplings $\lambda >0$ there is a unique $T_c^0(\lambda )>0$ satisfying $\inf \sigma (H_{T_c^0(\lambda )}^0)=0$ (see [8, Theorem 3.2] for $d=3$, and [9, Theorem 2.5] for $d=1,2$). In Sect. 3, in particular Remark 3.4, we shall show that there is a $\lambda _0>0$ such that the ground state of $H_{T_c^0(\lambda )}^0$ is even for couplings $\lambda \le \lambda _0$. By Lemma 2.4, $\inf \sigma (H_{T_c^0(\lambda )}^0)=\inf \sigma (H^{\Omega _0}_{T_c^0(\lambda )})=0$. Furthermore, for $T<T_c^0(\lambda )$, due to strict monotonicity of $H_T^0$ in *T*,

$$\begin{aligned} \inf \sigma (H_T^{\Omega _0})\le \inf \sigma _s(H_{T,\lambda }^0)<\inf \sigma (H_{T_c^0(\lambda )}^0)=0. \end{aligned}$$

Hence, $T_c^{\Omega _0}(\lambda )=T_c^0(\lambda )$ for $\lambda \le \lambda _0$. In particular, the minimum of $\sigma ( H_{T}^{\Omega _0})$ for $T=T_c^{\Omega _0}(\lambda )$ is attained at zero total momentum.

### Remark 2.6

The essential spectrum of $H_T^0$ satisfies $\inf \sigma _\textrm{ess}(H_T^0)=2T$ (see e.g. [10, Proof of Thm 3.7]). Hence, zero is an eigenvalue of $H_{T_c^0(\lambda )}^0$.

### Proof of Lemma 2.3

#### Proof of Lemma 2.3

Let $S_l$ be the shift to the right by *l* in the first component, i.e. $S_l \psi (x,y)= \psi (((x_1-l),{\tilde{x}}), (y_1-l,{\tilde{y}}))$. Let $\psi$ be a compactly supported function in $H_\textrm{sym}^1({\mathbb {R}}^{2d})$, the Sobolev space restricted to functions satisfying $\psi (x,y)=\psi (y,x)$. For *l* large enough, $S_l \psi$ is supported on the half-space and satisfies both Dirichlet and Neumann boundary conditions. The goal is to prove that $\lim _{l\rightarrow \infty } \langle S_l \psi , H^{\Omega _1}_T S_l \psi \rangle = \langle \psi , H^{\Omega _0}_T \psi \rangle$. Then, since compactly supported functions are dense in $H_\textrm{sym}^1({\mathbb {R}}^{2d})$, the claim follows.

Note that $\langle S_l \psi ,V S_l \psi \rangle = \langle \psi ,V \psi \rangle$. Furthermore, using the symmetry of $K_T$ in $p_1$ and $q_1$, one obtains

2.6 $$\begin{aligned} \langle S_l \psi ,K_T^{\Omega _1} S_l \psi \rangle= &   \int _{{\mathbb {R}}^{2d}}\overline{\widehat{\psi }(p,q)}K_T(p,q)\Big [\widehat{\psi }(p,q)\mp \widehat{\psi }((-p_1,{\tilde{p}}),q) e^{2 i l p_1}\nonumber \\    &   \mp \widehat{\psi }(p,(-q_1,{\tilde{q}}))e^{2 i l q_1}+\widehat{\psi }((-p_1,{\tilde{p}}),(-q_1,{\tilde{q}}))e^{2 i l (p_1+q_1)}\Big ] {\textrm{d}}p{\textrm{d}}q\nonumber \\ \end{aligned}$$

for *l* large enough such that $\psi$ is supported on the half-space. The first term is exactly $\langle \psi ,K_T^{\Omega _0} \psi \rangle$. Note that by the Schwarz inequality and Lemma 2.1, the function

2.7 $$\begin{aligned} (p,q)\mapsto \overline{\widehat{\psi }(p,q)}K_T(p,q)\widehat{\psi }((-p_1,{\tilde{p}}),q) \end{aligned}$$

is in $L^1({\mathbb {R}}^{2d})$ since $\psi \in H^1({\mathbb {R}}^{2d})$. By the Riemann–Lebesgue Lemma, the second term in (2.6) vanishes for $l\rightarrow \infty$. By the same argument, also the remaining terms vanish in the limit. $\square$

### Proof of Lemma 2.4

First, we prove

#### Lemma 2.7

For all $x,y\in {\mathbb {R}}$ we have

2.8 $$\begin{aligned} \frac{x+y}{\tanh (x)+\tanh (y)}\ge \frac{1}{2}\left( \frac{x}{\tanh (x)}+\frac{y}{\tanh (y)}\right) . \end{aligned}$$

#### Proof of Lemma 2.7

Suppose $\vert x \vert \ne \vert y \vert$. Without loss of generality we may assume that $x>\vert y \vert$. Since $\frac{x}{\tanh x}\ge \frac{y}{\tanh y}$,

2.9 $$\begin{aligned} \frac{x}{2 \tanh x} \frac{\tanh x-\tanh y}{\tanh x + \tanh y}\ge \frac{y}{2 \tanh y}\frac{\tanh x-\tanh y}{\tanh x + \tanh y}. \end{aligned}$$

This inequality is equivalent to (2.8), as can be seen using $\frac{\tanh x-\tanh y}{\tanh x + \tanh y}=\frac{2\tanh x}{\tanh x + \tanh y} -1=1-\frac{2\tanh y}{\tanh x + \tanh y}$ on the left and right side, respectively. By continuity, (2.8) also holds in the case $\vert x \vert = \vert y \vert$. $\square$

#### Proof of Lemma 2.4

Let *U* denote the unitary transform $U\psi (r,z)=\frac{1}{2^{d/2}}\psi ((r+z)/2,(z-r)/2)$ for $\psi \in L^2({\mathbb {R}}^{2d})$. By Lemma 2.7 we have

2.10 $$\begin{aligned} UH_T^{\Omega _0}U^\dagger= &   \frac{-\left( \nabla _r+\nabla _z\right) ^2-\left( \nabla _r-\nabla _z\right) ^2-2\mu }{\tanh \left( \frac{-\left( \nabla _r+\nabla _z\right) ^2-\mu }{2T}\right) +\tanh \left( \frac{-\left( \nabla _r-\nabla _z\right) ^2-\mu }{2T}\right) }+V(r)\nonumber \\\ge &   \frac{1}{2}\left( \frac{-\left( \nabla _r+\nabla _z\right) ^2-\mu }{\tanh \left( \frac{-\left( \nabla _r+\nabla _z\right) ^2-\mu }{2T}\right) }+V(r)\right) \nonumber \\  &   + \frac{1}{2}\left( \frac{-\left( \nabla _r-\nabla _z\right) ^2-\mu }{\tanh \left( \frac{-\left( \nabla _r-\nabla _z\right) ^2-\mu }{2T}\right) }+V(r)\right) . \end{aligned}$$

Both summands are unitarily equivalent to $\frac{1}{2}H_T^0\otimes {\mathbb {I}}$, where ${\mathbb {I}}$ acts on $L^2({\mathbb {R}}^d)$. Therefore, $\inf \sigma (H_T^{\Omega _0})\ge \inf \sigma (H_T^0)$.

For the second inequality let $f\in H^1({\mathbb {R}}^d)$ with $f(r)=f(-r)$ and $\psi _{\epsilon }(r,z)=e^{-\epsilon \sum _{j=1}^d\vert z_j \vert } f(r)$. Note that $\Vert \psi _\epsilon \Vert ^2_2=\frac{1}{\epsilon ^d}\Vert f \Vert _2^2$. Since the Fourier transform of $e^{-\epsilon \vert r_1 \vert }$ in $L^2({\mathbb {R}})$ is $(2/\pi )^{1/2} {\epsilon }/({\epsilon ^2+p_1^2})$, we have $\widehat{\psi _\epsilon }(p,q)= \widehat{f}(q) (2/\pi )^{d/2} \prod _{j=1}^d {\epsilon }/(\epsilon ^2 +p_j^2)$. Therefore,

2.11 $$\begin{aligned}  &   \frac{\langle \psi _\epsilon \vert UH_T^{\Omega _0}U^\dagger \psi _\epsilon \rangle }{\Vert \psi _\epsilon \Vert ^2}\nonumber \\  &   \quad =\frac{2^d}{\pi ^d \Vert f\Vert ^2}\int _{{\mathbb {R}}^{2d}} K_{T}(p+q,p-q)\prod _{j=1}^d \frac{\epsilon ^3}{(\epsilon ^2+p_j^2)^2} |\widehat{f}(q)|^2 {\textrm{d}}p {\textrm{d}}q \nonumber \\  &   \qquad +\frac{1}{\Vert f \Vert ^2} \int _{{\mathbb {R}}^d}V(r) |f(r)|^2{\textrm{d}}r\nonumber \\  &   \quad =\frac{2^d}{\pi ^d \Vert f\Vert ^2}\int _{{\mathbb {R}}^{2d}} K_{T}(\epsilon p+q,\epsilon p-q)\left( \prod _{j=1}^d \frac{1}{(1+p_j^2)^2}\right) |\widehat{f}(q)|^2 {\textrm{d}}p {\textrm{d}}q\nonumber \\  &   \qquad +\frac{1}{\Vert f \Vert ^2} \int _{{\mathbb {R}}^d}V(r) |f(r)|^2{\textrm{d}}r, \end{aligned}$$

where we substituted $p\rightarrow \epsilon p$ in the second step. By Lemma 2.1,

2.12 $$\begin{aligned}  &   K_{T}(\epsilon p+q,\epsilon p-q)\left( \prod _{j=1}^d \frac{1}{(1+p_j^2)^2}\right) |\widehat{f}(q)|^2 \nonumber \\    &   \qquad \le C (1+d \epsilon ^2+q^2)\left( \prod _{j=1}^d \frac{1}{1+p_j^2}\right) |\widehat{f}(q)|^2, \end{aligned}$$

which is integrable. With $\int _{{\mathbb {R}}}\frac{1}{(1+p_j^2)^2} {\textrm{d}}p_j=\pi /2$ it follows by dominated convergence that

2.13 $$\begin{aligned} \lim _{\epsilon \rightarrow 0} \frac{\langle \psi _\epsilon \vert UH_T^{\Omega _0}U^\dagger \psi _\epsilon \rangle }{\Vert \psi _\epsilon \Vert ^2}=\frac{\langle f \vert H_T^0 f \rangle }{\Vert f\Vert ^2}. \end{aligned}$$

Therefore, $$\inf \sigma (H_T^{\Omega _0})\le \inf \sigma _s(H_T^0)$$. $$\square $$

## Ground State of $H_{T_c^0(\lambda )}^0$

Let $T_c^0(\lambda )$ be the unique temperature satisfying $\inf \sigma (H_{T_c^0(\lambda )}^0)=0$ as in Remark 2.5, where $H_T^0$ was defined in (1.3). To study the ground state of $H_{T_c^0(\lambda )}^0$, it is convenient to apply the Birman–Schwinger principle. For $q\in {\mathbb {R}}^d$ let $B_{T}(\cdot ,q)$ denote the operator on $L^2({\mathbb {R}}^d)$ which acts as multiplication by $B_{T}(p,q)$ (defined in (2.2)) in momentum space. The Birman–Schwinger operator corresponding to $H_{T}^0$ acts on $L^2({\mathbb {R}}^d)$ and is given by

3.1 $$\begin{aligned} A_{T}^0=V^{1/2} B_{T}(\cdot ,0) \vert V\vert ^{1/2}, \end{aligned}$$

where we use the notation $V^{1/2}(x)=\textrm{sgn}(V(x))\vert V\vert ^{1/2}(x)$. This operator is compact [8, 9]. It follows from the Birman–Schwinger principle that $\sup \sigma (A_{T}^0)=1/\lambda$ exactly for $T=T_c^0(\lambda )$ and that the eigenvalue 0 of $H_{T_c^0(\lambda )}^0$ has the same multiplicity as the largest eigenvalue of $A_{T_c^0(\lambda )}^0$.

Let ${\mathcal {F}}:L^1({\mathbb {R}}^d)\rightarrow L^2({\mathbb {S}}^{d-1})$ act as ${\mathcal {F}}\psi (\omega )=\widehat{\psi }(\sqrt{\mu } \omega )$ and define $O_{\mu }=V^{1/2} {\mathcal {F}}^\dagger {\mathcal {F}}\vert V\vert ^{1/2}$ on $L^2({\mathbb {R}}^d)$. Furthermore, let

3.2 $$\begin{aligned} m_\mu (T)=\int _0^{\sqrt{2\mu }} B_{T}(t,0)t^{d-1} {\textrm{d}}t. \end{aligned}$$

Note that $m_\mu (T)= \mu ^{d/2-1}\left( \ln \left( \mu /T\right) +c_d\right) +o(1)$ for $T\rightarrow 0$, where $c_d$ is a number depending only on *d* [9, Prop 3.1].

The operator $O_\mu$ captures the singularity of $A_{T}^0$ as $T\rightarrow 0$. The following has been proved in [4, Lemma 2] for $d=3$ and in [9, Lemma 3.4] for $d=1,2$.

### Lemma 3.1

Let $d\in \{1,2,3\}$ and $\mu >0$ and let *V* satisfy Assumption 1.1. Then,

3.3 $$\begin{aligned} \sup _{T\in (0,\infty )} \left\Vert A_{T}^0 -m_\mu (T) O_{\mu }\right\Vert _\textrm{HS} <\infty , \end{aligned}$$

where $\Vert \cdot \Vert _\textrm{HS}$ denotes the Hilbert–Schmidt norm.

Thus, the asymptotic behavior of $\sup \sigma (A_{T}^0)$ depends on the largest eigenvalue of $O_\mu$. Note that $O_\mu$ is isospectral to $\mathcal {V}_\mu ={\mathcal {F}}V {\mathcal {F}}^\dagger$, since both operators are compact. The eigenfunction of $O_\mu$ corresponding to the eigenvalue $e_\mu$ is

3.4 $$\begin{aligned} \Psi (r):= V^{1/2}(r) j_d(r;\mu ), \end{aligned}$$

where $j_d$ was defined in (1.5). Note that

3.5 $$\begin{aligned} j_1(r;\mu )=\sqrt{\frac{2}{\pi }}\cos (\sqrt{\mu } r), \quad j_2(r;\mu )= J_0(\sqrt{\mu } \vert r \vert ), \nonumber \\ j_3(r;\mu )=\frac{2}{(2\pi )^{1/2}} \frac{\sin \sqrt{\mu } \vert r \vert }{\sqrt{\mu } \vert r \vert }, \end{aligned}$$

where $J_0$ is the Bessel function of order 0. Furthermore

3.6 $$\begin{aligned} e_\mu =\frac{1}{(2\pi )^{d/2}} \int _{{\mathbb {S}}^{d-1}} \widehat{V}(\sqrt{\mu }((1,0,...,0)-p)){\textrm{d}}p = \frac{1}{\vert {\mathbb {S}}^{d-1}\vert }\int _{{\mathbb {R}}^d} V(r)j_d(r;\mu )^2 {\textrm{d}}r \nonumber \\ \end{aligned}$$

The following asymptotics of $T_c^0(\lambda )$ for $\lambda \rightarrow 0$ were computed in [8, Theorem 3.3] and [9, Theorem 2.5]:

### Lemma 3.2

Let $\mu >0$, $d\in \{1,2,3\}$ and let *V* satisfy Assumption 1.1. Then

3.7 $$\begin{aligned} \lim _{\lambda \rightarrow 0} \Bigg \vert e_\mu m_\mu (T_c^0(\lambda ))-\frac{1}{\lambda }\Bigg \vert =\lim _{\lambda \rightarrow 0} \Bigg \vert e_\mu \mu ^{d/2-1}\ln \left( \frac{\mu }{T_c^0(\lambda )}\right) -\frac{1}{\lambda }\Bigg \vert <\infty . \end{aligned}$$

Lemma 3.1 does not only contain information about eigenvalues, but also about the corresponding eigenfunctions. In the following we prove that the eigenstate corresponding to the maximal eigenvalue of $A_{T}^0$ converges to $\Psi$.

### Lemma 3.3

Let $\mu >0$, $d\in \{1,2,3\}$ and let *V* satisfy Assumption 1.1.

(i) There is a $\lambda _0>0$ such that for $\lambda \le \lambda _0$, the largest eigenvalue of $A_{T_c^0(\lambda )}^0$ is non-degenerate.
(ii) Let $\lambda \le \lambda _0$ and let $\Psi _{T_c^0(\lambda )}$ be the eigenvector of $A_{T_c^0(\lambda )}^0$ corresponding to the largest eigenvalue, normalized such that $\Vert \Psi _{T_c^0(\lambda )}\Vert _2=\Vert \Psi \Vert _2$. Pick the phase of $\Psi _{T_c^0(\lambda )}$ such that $\langle \Psi _{T_c^0(\lambda )},\Psi \rangle \ge 0$. Then
  3.8 $$\begin{aligned} \lim _{\lambda \rightarrow 0} \frac{1}{\lambda } \Vert \Psi -\Psi _{T_c^0(\lambda )}\Vert _2^2<\infty \end{aligned}$$

### Remark 3.4

Let $\lambda _0$ be as in Lemma 3.3. By the Birman–Schwinger principle, the multiplicity of the largest eigenvalue of $A_{T_c^0(\lambda )}^0$ equals the multiplicity of the ground state of $H^0_{T_c^0(\lambda )}$. Hence, $H^0_{T_c^0(\lambda )}$ has a unique ground state for $\lambda \le \lambda _0$. For $d\ge 2$, since $H^0_{T_c^0(\lambda )}$ is rotation invariant, uniqueness of the ground state implies that the ground state is radial. For $d=1$, since $\Psi$ is even, the second part of Lemma 3.3 implies that $\Psi _{T_c^0(\lambda )}$ is even for small enough $\lambda$. Hence, also the ground state of $H^0_{T_c^0(\lambda )}$ is even for small $\lambda$.

It follows that for $\lambda \le \lambda _0$ we have $T_c^{\Omega _0}(\lambda )=T_c^0(\lambda )$ as discussed in Remark 2.5.

For values of $\lambda$ such that the operator $H_{T_c^0(\lambda )}^0$ has a non-degenerate eigenvalue at the bottom of its spectrum let $\Phi _{\lambda }$ be the corresponding eigenfunction, with normalization and phase chosen such that $\Psi _{T_c^0(\lambda )}=V^{1/2}\Phi _{\lambda }$. The next Lemma with regularity and convergence properties of $\Phi _{\lambda }$ will be useful.

### Lemma 3.5

Let $d\in \{1,2,3\}$, $\mu >0$ and let *V* satisfy Assumption 1.1. For all $0<\lambda <\infty$ such that $H_{T_c^0(\lambda )}^0$ has a non-degenerate ground state $\Phi _\lambda$, we have

(i) $\vert \widehat{\Phi _\lambda }(p)\vert \le \frac{C(\lambda )}{1+p^2} \vert \widehat{V \Phi }_\lambda (p)\vert \le \frac{C(\lambda ) \Vert V\Vert _1^{1/2} \Vert \Psi \Vert _2 }{1+p^2}$ for some number $C(\lambda )$ depending on $\lambda$,
(ii) $p \mapsto \widehat{\Phi }_\lambda (p)$ is continuous,
(iii) $$\Vert \widehat{\Phi }_\lambda \Vert _1<\infty $$ and $$\Vert \Phi _\lambda \Vert _\infty <\infty $$.

Furthermore, in the limit $\lambda \rightarrow 0$

(iv) $$\Vert \widehat{\Phi }_\lambda \chi _{p^2>2\mu }\Vert _1=O(\lambda )$$,
(v) $$\Vert \widehat{\Phi }_\lambda \Vert _1=O(1)$$,
(vi) and in particular $\Vert \Phi _\lambda \Vert _\infty =O(1)$.

In three dimensions, because of the additional condition (1.7), we need to compute the limit of $\Phi _\lambda$.

### Lemma 3.6

Let $d=3$, $\mu >0$ and let *V* satisfy Assumption 1.1. Then $\Vert \Phi _\lambda -j_3 \Vert _\infty =O(\lambda ^{1/2})$ as $\lambda \rightarrow 0$.

### Proof of Lemma 3.3

#### Proof of Lemma 3.3

*(i)* The proof uses ideas from [7, Proof of Thm 1]. Let $M_T=B_T(\cdot ,0)-m_\mu (T) {\mathcal {F}}^\dagger {\mathcal {F}}$. By Lemma 3.1, for $\lambda$ small enough the operator $1-\lambda V^{1/2} M_T \vert V\vert ^{1/2}$ is invertible for all *T*. Then we can write

3.9 $$\begin{aligned} 1-\lambda A_{T}^0 = (1-\lambda V^{1/2} M_T \vert V\vert ^{1/2})\Bigg (1-\frac{\lambda m_\mu (T) }{1-\lambda V^{1/2} M_T \vert V\vert ^{1/2} }V^{1/2} {\mathcal {F}}^\dagger {\mathcal {F}}\vert V\vert ^{1/2} \Bigg ). \nonumber \\ \end{aligned}$$

Recall that the largest eigenvalue of $A_{T_c^0(\lambda )}^0$ equals $1/\lambda$. Hence, 1 is an eigenvalue of

3.10 $$\begin{aligned} \frac{\lambda m_\mu (T_c^0(\lambda )) }{1-\lambda V^{1/2} M_{T_c^0(\lambda )} \vert V\vert ^{1/2} }V^{1/2} {\mathcal {F}}^\dagger {\mathcal {F}}\vert V\vert ^{1/2}, \end{aligned}$$

and it has the same multiplicity as the eigenvalue $1/\lambda$ of $A_{T_c^0(\lambda )}^0$. This operator is isospectral to the self-adjoint operator

3.11 $$\begin{aligned} {\mathcal {F}}\vert V\vert ^{1/2}\frac{\lambda m_\mu ({T_c^0(\lambda )}) }{1-\lambda V^{1/2} M_{T_c^0(\lambda )} \vert V\vert ^{1/2} }V^{1/2} {\mathcal {F}}^\dagger . \end{aligned}$$

Note that the operator difference

3.12 $$\begin{aligned}  &   {\mathcal {F}}\vert V\vert ^{1/2}\frac{1 }{1-\lambda V^{1/2} M_{T_c^0(\lambda )} \vert V\vert ^{1/2} }V^{1/2} {\mathcal {F}}^\dagger - \mathcal {V}_\mu \nonumber \\    &   \qquad =\lambda {\mathcal {F}}\vert V\vert ^{1/2}\frac{ V^{1/2} M_{T_c^0(\lambda )} \vert V\vert ^{1/2}}{1-\lambda V^{1/2} M_{T_c^0(\lambda )} \vert V\vert ^{1/2} }V^{1/2} {\mathcal {F}}^\dagger \end{aligned}$$

has operator norm of order $O(\lambda )$ according to Lemma 3.1. By assumption, the largest eigenvalue of $\mathcal {V}_\mu$ has multiplicity one, and $\lambda m_\mu ({T_c^0(\lambda )}) e_\mu =1+O(\lambda )$ by Lemma 3.2. Let $\alpha <1$ be the ratio between the second largest and the largest eigenvalue of $\mathcal {V}_\mu$. The second largest eigenvalue of $\lambda m_\mu ({T_c^0(\lambda )}) \mathcal {V}_\mu$ is of order $\alpha +O(\lambda )$. Therefore, the largest eigenvalue of (3.11) must have multiplicity 1 for small enough $\lambda$, and it is of order $1+O(\lambda )$, whereas the rest of the spectrum lies below $\alpha +O(\lambda )$. Hence, 1 is the maximal eigenvalue of (3.11) and it has multiplicity 1 for small enough $\lambda$.

*(ii)* Note that $\Psi _{T_c^0(\lambda )}$ is an eigenvector of (3.10) with eigenvalue 1. Furthermore, let $\psi _{\lambda }$ be a normalized eigenvector of (3.11) with eigenvalue 1. Then

3.13 $$\begin{aligned} \tilde{\Psi }_{T_c^0(\lambda )}=\frac{\Vert \Psi \Vert _2}{\Vert \frac{1 }{(1-\lambda V^{1/2} M_{T_c^0(\lambda )} \vert V\vert ^{1/2}) }V^{1/2} {\mathcal {F}}^\dagger \psi _{\lambda }\Vert _2}\frac{1 }{1-\lambda V^{1/2} M_{T_c^0(\lambda )} \vert V\vert ^{1/2} }V^{1/2} {\mathcal {F}}^\dagger \psi _{\lambda } \nonumber \\ \end{aligned}$$

agrees with $\Psi _{T_c^0(\lambda )}$ up to a constant phase. Since $\Vert \Psi _{T_c^0(\lambda )}-\Psi \Vert ^2 \le \Vert \tilde{\Psi }_{T_c^0(\lambda )}-\Psi \Vert ^2$, it suffices to prove that the latter is of order $O(\lambda )$ for a suitable choice of phase for $\psi _{\lambda }$.

Let $\psi (p)=\frac{1}{\vert {\mathbb {S}}^{d-1}\vert ^{1/2}}$. This is the eigenfunction of $\mathcal {V}_\mu$ corresponding to the maximal eigenvalue, and $\Psi =V^{1/2} {\mathcal {F}}^\dagger \psi$. In particular, for all $\phi \in L^2({\mathbb {S}}^{d-1})$,

3.14 $$\begin{aligned} \langle \phi , \mathcal {V}_\mu \phi \rangle \le e_\mu |\langle \phi , \psi \rangle |^2+\alpha e_\mu (\Vert \phi \Vert _2^2-|\langle \phi , \psi \rangle |^2). \end{aligned}$$

We choose the phase of $\psi _{\lambda }$ such that $\langle \psi _{\lambda }, \psi \rangle \ge 0$. We shall prove that $\Vert \psi _{\lambda }-\psi \Vert _2^2=O(\lambda )$. We have by (3.12) and (3.14)

3.15 $$\begin{aligned} O(\lambda )= &   \langle \psi _{\lambda },(1-\lambda m_\mu ({T_c^0(\lambda )}) \mathcal {V}_\mu )\psi _{\lambda }\rangle \nonumber \\\ge &   1-\lambda m_\mu ({T_c^0(\lambda )}) e_\mu \vert \langle \psi _\lambda ,\psi \rangle \vert ^2-\lambda m_\mu ({T_c^0(\lambda )})\alpha e_\mu (1-\vert \langle \psi _\lambda ,\psi \rangle \vert ^2 )\nonumber \\= &   O(\lambda )+(1-\alpha ) (1-\vert \langle \psi _\lambda ,\psi \rangle \vert ^2 ), \end{aligned}$$

where we used Lemma 3.2 for the last equality. In particular, $1-\vert \langle \psi _\lambda ,\psi \rangle \vert ^2=O(\lambda )$. Hence,

3.16 $$\begin{aligned} \Vert \psi -\psi _{\lambda } \Vert _2^2 =2 (1-\langle \psi _{\lambda }, \psi \rangle )= 2 \frac{1-\langle \psi _{\lambda }, \psi \rangle ^2}{1+\langle \psi _{\lambda }, \psi \rangle } =O(\lambda ). \end{aligned}$$

Using Lemma 3.1 and that $V^{1/2} {\mathcal {F}}^\dagger : L^2({\mathbb {S}}^{d-1})\rightarrow L^2({\mathbb {R}}^d)$ is a bounded operator, and subsequently (3.16) we obtain

3.17 $$\begin{aligned} \frac{1 }{1-\lambda V^{1/2} M_{T_c^0(\lambda )} \vert V\vert ^{1/2} }V^{1/2} {\mathcal {F}}^\dagger \psi _\lambda= &   V^{1/2} {\mathcal {F}}^\dagger \psi _{\lambda }+O(\lambda )\nonumber \\  = &   V^{1/2} {\mathcal {F}}^\dagger \psi +O(\lambda ^{1/2}), \end{aligned}$$

where $O(\lambda )$ here denotes a vector with $L^2$-norm of order $O(\lambda )$. Furthermore,

3.18 $$\begin{aligned}  &   \left| \Vert (1-\lambda V^{1/2} M_{T_c^0(\lambda )} \vert V\vert ^{1/2})^{-1} V^{1/2} {\mathcal {F}}^\dagger \psi _{\lambda }\Vert _2-\Vert V^{1/2} {\mathcal {F}}^\dagger \psi \Vert _2 \right| \nonumber \\  &   \quad \le \Vert (1-\lambda V^{1/2} M_{T_c^0(\lambda )} \vert V\vert ^{1/2})^{-1}V^{1/2} {\mathcal {F}}^\dagger \psi _{\lambda }-V^{1/2} {\mathcal {F}}^\dagger \psi \Vert _2 =O(\lambda ^{1/2}). \nonumber \\ \end{aligned}$$

In total, we have

3.19 $$\begin{aligned} \tilde{\Psi }_{T_c^0(\lambda )}= &   \frac{\Vert \Psi \Vert _2}{\Vert V^{1/2} {\mathcal {F}}^\dagger \psi \Vert _2+O(\lambda ^{1/2})}(V^{1/2} {\mathcal {F}}^\dagger \psi +O(\lambda ^{1/2}))\nonumber \\  = &   \frac{\Vert \Psi \Vert _2}{\Vert V^{1/2} {\mathcal {F}}^\dagger \psi \Vert _2}V^{1/2} {\mathcal {F}}^\dagger \psi +O(\lambda ^{1/2})\nonumber \\= &   \Psi +O(\lambda ^{1/2}). \end{aligned}$$

$$\square $$

### Regularity and Convergence of $\Phi _\lambda$

In this section, we prove Lemma 3.5 and Lemma 3.6. The following standard results (see e.g. [11, Sections 11.3, 5.1]) will be helpful:

#### Lemma 3.7

(i) Let $V \in L^{p}({\mathbb {R}}^d)$, where $p=1$ for $d=1$, $p>1$ for $d=2$ and $p=3/2$ for $d=3$. Let $\psi \in H^1({\mathbb {R}}^d)$. Then $V^{1/2} \psi \in L^2({\mathbb {R}}^d)$.
(ii) If $V\in L^1({\mathbb {R}}^d)$ and $\psi \in L^2({\mathbb {R}}^d)$, then $V^{1/2} \psi \in L^1({\mathbb {R}}^d)$ and hence $\widehat{V^{1/2} \psi }$ is continuous and bounded.
(iii) For $1\le t$, $\Vert \widehat{V^{1/2} \psi } \Vert _s \le C \Vert V \Vert _t^{1/2} \Vert \psi \Vert _2$, where $s=2t/(t-1)$ and *C* is some constant independent of $\psi$ and *V*.
(iv) Let *f* be a radial, measurable function on ${\mathbb {R}}^3$ and $p\ge 1$. Then there is a constant *C* independent of *f* such that $\sup _{p_1 \in {\mathbb {R}}} \Vert f(p_1, \cdot ) \Vert _{L^p( {\mathbb {R}}^{2})} = \Vert f(0, \cdot ) \Vert _{L^p( {\mathbb {R}}^{2})}\le C(\Vert f \Vert _{L^p({\mathbb {R}}^3)}^p+\Vert f \Vert _{L^\infty ({\mathbb {R}}^3)}^p)^{1/p}$.

#### Proof

For (i) and (ii) see e.g. [11, Sections 11.3, 5.1]. For (iii) let $s\ge 2$. Applying the Hausdorff-Young and Hölder inequalities gives

3.20 $$\begin{aligned} \Vert \widehat{V^{1/2} \psi } \Vert _s \le C \Vert V^{1/2} \psi \Vert _p \le C \Vert V \Vert _t^{1/2} \Vert \psi \Vert _2, \end{aligned}$$

where $1=1/p+1/s$ and $1=p/2t+p/2$. Hence, $s=2t/(t-1)$.

For (iv) we write

3.21 $$\begin{aligned}  &   \Vert f(p_1, \cdot ) \Vert _{L^p( {\mathbb {R}}^{2})}^p \nonumber \\  &   \quad = 2\pi \int _0^\infty |f(\sqrt{p_1^2+t^2})|^p t {\textrm{d}}t =2\pi \int _{|p_1|}^\infty |f(s)|^p s{\textrm{d}}s \le \Vert f(0, \cdot ) \Vert _{L^p( {\mathbb {R}}^{2})}^p \nonumber \\  &   \quad \le 2\pi \int _{0}^1 |f(s)|^p {\textrm{d}}s +2\pi \int _{0}^\infty |f(s)|^p s^2 {\textrm{d}}s \le 2\pi \Vert f \Vert _\infty ^p+\frac{1}{2}\Vert f \Vert ^p_p, \nonumber \\ \end{aligned}$$

where in the second step we substituted $s=\sqrt{p_1^2+t^2}$ and in the third step we used $s\le \max \{1,s^2\}$. $\square$

#### Proof of Lemma 3.5

The eigenvalue equation $H_{T_c^0(\lambda )}^0 \Phi _\lambda =0$ implies that

3.22 $$\begin{aligned} \widehat{\Phi }_{\lambda }(p)=\lambda B_{T_c^0(\lambda )}(p,0)\widehat{V\Phi _\lambda }(p). \end{aligned}$$

Part (i) follows with Lemma 2.1 and 3.7(iii) and the normalization $\Vert V^{1/2}\Phi _\lambda \Vert _2=\Vert \Psi \Vert _2$. For part (ii), note that $p \mapsto B_{T}(p,0)$ is continuous for $T>0$. Since $\Phi _\lambda \in H^1({\mathbb {R}}^d)$, continuity of $\widehat{V\Phi _\lambda }$ follows by Lemma 3.7(i) and (ii).

Note that $\Vert \Phi _\lambda \Vert _\infty {\le } (2\pi )^{-d/2} \Vert \widehat{\Phi _\lambda } \Vert _1 {=}(2\pi )^{-d/2} (\Vert \widehat{\Phi _\lambda } \chi _{p^2<2\mu }\Vert _1 + \Vert \widehat{\Phi _\lambda } \chi _{p^2>2\mu } \Vert _1 )$. In particular, the second part of (iii) and (vi) follow from the first part of (iii) and (v), respectively. Using (3.22) and $\Vert \Psi _{T_c^0(\lambda )}\Vert _2= \Vert \Psi \Vert _2$ we obtain

3.23 $$\begin{aligned} \Vert \widehat{\Phi _\lambda } \chi _{p^2<2\mu }\Vert _1\le &   \lambda m_\mu (T_c^0(\lambda ))|{\mathbb {S}}^{d-1}| \Vert \widehat{V^{1/2} \Psi _{T_c^0(\lambda )}}\Vert _\infty \nonumber \\\le &   \lambda m_\mu (T_c^0(\lambda ))|{\mathbb {S}}^{d-1}| \Vert V\Vert _1^{1/2} \Vert \Psi \Vert _2, \end{aligned}$$

where $m_\mu$ was defined in (3.2). In particular, for fixed $\lambda$, $\Vert \widehat{\Phi _\lambda } \chi _{p^2<2\mu }\Vert _1 <\infty$ and from Lemma 3.2 it follows that $\Vert \widehat{\Phi _\lambda } \chi _{p^2<2\mu }\Vert _1$ is bounded for $\lambda \rightarrow 0$.

It only remains to prove that $\Vert \widehat{\Phi _\lambda } \chi _{p^2>2\mu }\Vert _1$ is bounded for fixed $\lambda$ and is $O(\lambda )$ for $\lambda \rightarrow 0$. By (2.3) $B_{T}(p,0)\chi _{p^2>2\mu }\le C/(1+p^2)$ for some *C* independent of *T*. Using (3.22) and applying Hölder’s inequality and Lemma 3.7(iii),

3.24 $$\begin{aligned} \Vert \widehat{\Phi _\lambda } \chi _{p^2>2\mu }\Vert _s\le &   C \lambda \left\Vert \frac{1}{1+\vert \cdot \vert ^2}\right\Vert _p \Vert \widehat{V^{1/2} \Psi _{T_c^0(\lambda )}} \Vert _q \nonumber \\\le &   C \lambda \left\Vert \frac{1}{1+\vert \cdot \vert ^2}\right\Vert _p \Vert V\Vert _t^{1/2} \Vert \Psi \Vert _2, \end{aligned}$$

where $1/s=1/p+1/q$ and $q=2t/(t-1)$. For $d=1$ the claim follows with the choice $t=p=1$. For $d=2$, $V\in L^{1+\epsilon }$ for some $0<\epsilon \le 1$. With the choice $t=1+\epsilon , p=2t/(t+1)>1$ the claim follows.

For $d=3$, we may choose $1 \le t\le 3/2$ and $3/2<p\le \infty$ which gives

3.25 $$\begin{aligned} \Vert \widehat{\Phi }_\lambda \chi _{p^2>2\mu }\Vert _s=O(\lambda ) \end{aligned}$$

for all $6/5<s\le \infty$. We use a bootstrap argument to decrease *s* to one. Let us use the short notation *B* for multiplication with $B_{T}(p,0)$ in momentum space and $F:L^2({\mathbb {R}}^d)\rightarrow L^2({\mathbb {R}}^d)$ the Fourier transform. Using (3.22) one can find by induction that

3.26 $$\begin{aligned} \widehat{\Phi _\lambda } \chi _{p^2>2\mu }= &   \lambda ^{n} (\chi _{p^2>2\mu } B F V F^\dagger )^n \widehat{\Phi _\lambda } \chi _{p^2>2\mu }\nonumber \\  &   +\sum _{j=1}^n \lambda ^{j}(\chi _{p^2>2\mu } B F VF^\dagger )^j \widehat{\Phi _\lambda }\chi _{p^2<2\mu } \end{aligned}$$

for any $n\in {\mathbb {N}}$. The strategy is to prove that applying $\chi _{p^2>2\mu } B F VF^\dagger$ to an $L^r$ function will give a function in $L^s\cap L^\infty$, where $s/r< c <1$ for some fixed constant *c*. For *n* large enough, the first term will be in $L^1$, while the second term is in $L^1$ for all *n* since $\widehat{\Phi _\lambda }\chi _{p^2<2\mu }$ is $L^1$.

#### Lemma 3.8

Let $V\in L^1\cap L^{3/2+\epsilon }({\mathbb {R}}^3)$ for some $0<\epsilon \le 1/2$ and let $1\le r \le 3/2$ and $f \in L^r({\mathbb {R}}^3)$. Let $2\ge q\ge r$ and $3/2<t\le \infty$.

(i) Then,
  3.27 $$\begin{aligned} \Vert \chi _{p^2>2\mu } B F V F^\dagger f \Vert _s \le C(r,q)\left\Vert \frac{1}{1+|\cdot |^2}\right\Vert _t \Vert V \Vert _{q}\Vert f \Vert _{r} \end{aligned}$$
  where $1/s=1/t+1/r-1/q$ and $C(r,q) < \infty$. (For $s<1$, $\Vert \cdot \Vert _s$ has to be interpreted as $\Vert f \Vert _s=\left( \int _{{\mathbb {R}}^3} |f(p)|^s {\textrm{d}}p\right) ^{1/s}$.)
(ii) Let $c=\frac{\epsilon }{(3+\epsilon )(3+2\epsilon )}>0$ and let $r/(1+c)\le s \le \infty$. Then $\Vert \chi _{p^2>2\mu } B F V F^\dagger f \Vert _s \le C(r,s) \Vert f \Vert _{r}$ for $C(r,s)<\infty$.

#### Proof of Lemma 3.8

(i): Using (2.3) we have $|\chi _{p^2>2\mu } B F V F^\dagger f (p)|\le \frac{C}{1+p^2} | {\widehat{V}} * f (p)|$. By the Young and Hausdorff-Young inequalities, the convolution satisfies

3.28 $$\begin{aligned} \Vert {\widehat{V}} * f \Vert _p \le C(q,r) \Vert V \Vert _q \Vert f \Vert _r \end{aligned}$$

for some finite constant *C*(*q*, *r*), where $1/p=1/r-1/q$. The claim follows from Hölder’s inequality.

(ii): For fixed *r* and choosing *q*, *t* in the range $r \le q \le 3/2+\epsilon$ and $3/2+\epsilon /2 \le t \le \infty$, $s=(1/t+1/r-1/q)^{-1}$ can take all values in $[r/(1+c), \infty ]$. The claim follows immediately from (i). $\square$

Let *n* be the smallest integer such that $\frac{7}{5} \frac{1}{(1+c)^n}\le 1$. To bound the first term in (3.26), recall from (3.25) that $\Vert \widehat{\Phi }_\lambda \chi _{p^2>2\mu }\Vert _{s}=O(\lambda )$ for $s=7/5$. We apply the second part of Lemma 3.8*n* times. After the *j*th step, we have $\Vert (\chi _{p^2>2\mu } B F V F^\dagger )^j \widehat{\Phi _\lambda } \chi _{p^2>2\mu }\Vert _s=O(\lambda )$ for $s=\frac{7}{5} \frac{1}{(1+c)^j}$. In the *n*th step we pick $s=1$ and obtain $\Vert (\chi _{p^2>2\mu } B F V F^\dagger )^n \widehat{\Phi _\lambda } \chi _{p^2>2\mu }\Vert _1=O(\lambda )$. To bound the second term in (3.26) recall that $\Vert \widehat{\Phi _\lambda }\chi _{p^2<2\mu }\Vert _1=O(1)$. Applying the first part of Lemma 3.8 with $r=1, t=q=3/2+\epsilon$ implies that

3.29 $$\begin{aligned}  &   \left\Vert \sum _{j=1}^n \lambda ^{j}(\chi _{p^2>2\mu } B F V F^\dagger )^j \widehat{\Phi _\lambda }\chi _{p^2<2\mu }\right\Vert _1 \nonumber \\  &   \quad \le \sum _{j=1}^n \lambda ^{j}\left( C(1,3/2+\epsilon )\left\Vert \frac{1}{1+|\cdot |^2}\right\Vert _{3/2+\epsilon } \Vert V \Vert _{3/2+\epsilon }\right) ^j\Vert \widehat{\Phi _\lambda }\chi _{p^2<2\mu } \Vert _{1}\nonumber \\    &   \quad =O(\lambda ). \end{aligned}$$

It follows that $\Vert \widehat{\Phi }_\lambda \chi _{p^2>2\mu }\Vert _1$ is finite and $O(\lambda )$ for $d=3$. $\square$

#### Proof of Lemma 3.6

Using the eigenvalue equation, Eq. (3.22), we write

3.30 $$\begin{aligned} \begin{aligned} \Phi _\lambda (r)=&\int _{\vert p \vert >\sqrt{2\mu }}\frac{e^{i p \cdot r}}{(2\pi )^{3/2}} \widehat{\Phi }_\lambda (p) {\text {d}}p +\lambda \int _{\vert p \vert<\sqrt{2\mu }}\frac{e^{i p \cdot (r-r')}-e^{i \sqrt{\mu }\frac{p}{|p|} \cdot (r-r')}}{(2\pi )^3} \\  &\times B_{T_c^0(\lambda )}(p,0)|V|^{1/2} (r') \Psi _{T_c^0(\lambda )}(r') {\text {d}}p {\text {d}}r' \\  &+\lambda \int _{\vert p \vert<\sqrt{2\mu }}\frac{e^{i \sqrt{\mu }\frac{p}{|p|} \cdot (r-r')}}{(2\pi )^3} B_{T_c^0(\lambda )}(p,0)|V|^{1/2} (r') \\  &\times (\Psi _{T_c^0(\lambda )}(r')-V^{1/2} (r') j_3(r')) {\text {d}}p {\text {d}}r' \\  &+\lambda \int _{\vert p \vert <\sqrt{2\mu }}\frac{e^{i \sqrt{\mu }\frac{p}{|p|} \cdot (r-r')}}{(2\pi )^3} B_{T_c^0(\lambda )}(p,0)V(r')j_3(r') {\text {d}}p {\text {d}}r'. \end{aligned} \end{aligned}$$

We prove that the first three terms have $L^\infty$-norm of order $O(\lambda ^{1/2})$. For the first term this follows from Lemma 3.5. For the second term in (3.30), we proceed as in the proof of [8, Lemma 3.1]. First, integrate over the angular variables

3.31 $$\begin{aligned}  &   \int _{\vert p \vert<\sqrt{2\mu }}\left[ e^{i p \cdot (r-r')}-e^{i \sqrt{\mu }\frac{p}{|p|} \cdot (r-r')}\right] B_{T_c^0(\lambda )}(p,0){\textrm{d}}p\nonumber \\  &   \quad =\int _{\vert p \vert <\sqrt{2\mu }}\left[ \frac{\sin \vert p\vert \vert r-r'\vert }{ \vert p\vert \vert r-r'\vert }-\frac{\sin \sqrt{\mu } \vert r-r'\vert }{ \sqrt{\mu } \vert r-r'\vert }\right] B_{T_c^0(\lambda )}(\vert p\vert ,0) |p|^2 {\textrm{d}}\vert p\vert , \nonumber \\ \end{aligned}$$

where we slightly abuse notation writing $B_T(|p|,0)$ for the radial function $B_T(p,0)$. Bounding the absolute value of this using $\vert \sin x/x-\sin y/y|<C |x-y|/|x+y|$ and $B_{T}(p,0)\le 1/|p^2-\mu |$ gives

3.32 $$\begin{aligned} 3.31 \le C\int _{\vert p \vert<\sqrt{2\mu }}\frac{|p|^2}{( \vert p\vert +\sqrt{\mu })^2} {\textrm{d}}\vert p\vert =:{\tilde{C}}<\infty . \end{aligned}$$

In particular, the second term in (3.30) is bounded uniformly in *r* by

3.33 $$\begin{aligned} \lambda \frac{{\tilde{C}}}{(2\pi )^3} \Vert V\Vert _1^{1/2} \Vert \Psi _{T_c^0(\lambda )} \Vert _2, \end{aligned}$$

which is of order $O(\lambda )$.

To bound the absolute value of the third term in (3.30), we pull the absolute value into the integral, carry out the integration over *p* and use the Schwarz inequality in $r'$. This results in the bound

3.34 $$\begin{aligned} \lambda \frac{\vert {\mathbb {S}}^2 \vert }{(2\pi )^3} m_\mu (T_c^0(\lambda )) \Vert V\Vert _1^{1/2} \Vert \Psi _{T_c^0(\lambda )} -\Psi \Vert _2. \end{aligned}$$

By Lemma 3.2, $\lambda m_\mu (T_c^0(\lambda ))$ is bounded and by Lemma 3.3, $\Vert \Psi _{T_c^0(\lambda )}- \Psi \Vert _2$ decays like $\lambda ^{1/2}$ for small $\lambda$.

The fourth term in (3.30) equals $\lambda m_\mu (T_c^0(\lambda )) {\mathcal {F}}^\dagger {\mathcal {F}}V j_3$, where we carried out the radial part of the *p* integration. Recall that $j_3={\mathcal {F}}^\dagger 1_{{\mathbb {S}}^2}$ and $\mathcal {V}_\mu 1_{{\mathbb {S}}^2} =e_\mu 1_{{\mathbb {S}}^2}$, where $1_{{\mathbb {S}}^2}$ is the constant function with value 1 on ${\mathbb {S}}^2$. Hence, ${\mathcal {F}}^\dagger {\mathcal {F}}V j_3={\mathcal {F}}^\dagger \mathcal {V}_\mu 1_{{\mathbb {S}}^2} = e_\mu j_3$ and the fourth term in (3.30) equals $\lambda m_\mu (T_c^0(\lambda )) e_\mu j_3$. By Lemma 3.2, $\lambda m_\mu (T_c^0(\lambda )) e_\mu = 1+O(\lambda )$ as $\lambda \rightarrow 0$. Thus, $\Vert \Phi _\lambda -j_3 \Vert _\infty = \vert \lambda m_\mu (T_c^0(\lambda )) e_\mu -1 \vert \Vert j_3 \Vert _\infty +O(\lambda )=O(\lambda )$. $\square$

## Proof of Theorem 1.3

Instead of directly looking at $H_T^{\Omega _1}$, we extend the domain to $L^2({\mathbb {R}}^{2d})$ by extending the wavefunctions (anti)symmetrically across the boundary. Recall that ${\tilde{x}}$ denotes the vector containing all but the first component of *x*. The half-space operator $H_T^{\Omega _1}$ with Dirichlet/Neumann boundary conditions is unitarily equivalent to

4.1 $$\begin{aligned} H^\textrm{ext}_{T}=K_{T}^{{\mathbb {R}}^d}-\lambda V(x-y) \chi _{\vert x_1-y_1 \vert<\vert x_1+y_1 \vert }-\lambda V(x_1+y_1,{\tilde{x}}-{\tilde{y}}) \chi _{\vert x_1+y_1 \vert <\vert x_1-y_1 \vert } \nonumber \\ \end{aligned}$$

on $L^2({\mathbb {R}}^{d}\times {\mathbb {R}}^{d})$ restricted to functions antisymmetric/symmetric under swapping $x_1\leftrightarrow -x_1$ and symmetric under exchange of $x \leftrightarrow y$. Next, we express $H^\textrm{ext}_{T}$ in relative and center of mass coordinates $r=x-y$ and $z=x+y$. Let *U* be the unitary on $L^2({\mathbb {R}}^{2d})$ given by $U\psi (r,z)=2^{-d/2} \psi ((r+z)/2,(z-r)/2)$. Then

4.2 $$\begin{aligned} H^1_{T}:= U H^\textrm{ext}_{T}U^\dagger =UK_{T}^{{\mathbb {R}}^d}U^\dagger -\lambda V(r) \chi _{\vert r_1 \vert<\vert z_1 \vert }-\lambda V(z_1,{\tilde{r}}) \chi _{\vert z_1 \vert <\vert r_1 \vert } \end{aligned}$$

on $L^2({\mathbb {R}}^{2d})$ restricted to functions antisymmetric/symmetric under swapping $r_1\leftrightarrow z_1$ and symmetric in *r*. The spectra of $H^1_{T}$ and $H_T^{\Omega _1}$ agree.

For an upper bound on $\inf \sigma (H_{T}^1)$, we restrict $H_{T}^1$ to zero momentum in the translation invariant center of mass directions and call the resulting operator ${\tilde{H}}_{T}^1$. The operator ${\tilde{H}}_{T}^1$ acts on $\{\psi \in L^2({\mathbb {R}}^d\times {\mathbb {R}})| \psi (r,z_1)=\psi (-r,z_1)=\mp \psi ((z_1,{\tilde{r}}),r_1)\}$. The kinetic part of ${\tilde{H}}_{T}^1$ reads as

4.3 $$\begin{aligned} {\tilde{K}}_{T}(r,z_1;r',z_1')=\int _{{\mathbb {R}}^{d+1}} \frac{e^{i p(r-r')+iq_1(z_1-z_1')}}{(2\pi )^{d+1}} B_{T}^{-1}(p,(q_1,{\tilde{0}})) {\textrm{d}}p {\textrm{d}}q_1. \end{aligned}$$

An important property is the continuity of $\inf \sigma (H_T^1)$, proven in Sect. 4.1.

### Lemma 4.1

Let $d\in \{1,2,3\}$ and let *V* satisfy Assumption 1.1. Then $\inf \sigma (H_T^0)$, $\inf \sigma (H_T^{\Omega _0})$ and $\inf \sigma (H_T^1)$ depend continuously on *T* for $T>0$.

To prove Theorem 1.3 we show that there is a $\lambda _1>0$ such that for $\lambda \le \lambda _1$, $\inf \sigma (H_{T_c^{\Omega _0}(\lambda )}^1)\le \inf \sigma ({\tilde{H}}_{T_c^{\Omega _0}(\lambda )}^1)<0$. For all $T<T_c^{\Omega _0}(\lambda )$ we have by Lemma 2.3 that $\inf \sigma (H_{T}^1)\le \inf \sigma (H_{T}^{\Omega _0})<0$. By continuity (Lemma 4.1) there is an $\epsilon >0$ such that $\inf \sigma (H_{T}^1)<0$ for all $T<T_c^{\Omega _0}(\lambda )+\epsilon$. Therefore, $T_c^{\Omega _1}(\lambda )> T_c^{\Omega _0}(\lambda )$.

To prove that $\inf \sigma ({\tilde{H}}_{T_c^{\Omega _0}(\lambda )}^1)<0$ for small enough $\lambda$, we pick a suitable family of trial states $\psi _\epsilon (r,z_1)$. Let $\lambda$ be such that $T_c^{\Omega _0}(\lambda )=T_c^0(\lambda )$ and $H_{T_c^0(\lambda )}^0$ has a unique and radial ground state $\Phi _\lambda$. According to Remark 3.4, this is the case for $0<\lambda \le \lambda _0$. We choose the trial states

4.4 $$\begin{aligned} \psi _\epsilon (r,z_1)=\Phi _{\lambda }(r)e^{-\epsilon \vert z_1 \vert }\mp \Phi _{\lambda }(z_1, {\tilde{r}})e^{-\epsilon \vert r_1 \vert }, \end{aligned}$$

with the − sign for Dirichlet and $+$ for Neumann boundary conditions. Since $\Phi _{\lambda }(r)=\Phi _{\lambda }(-r)=\Phi _{\lambda }(-r_1,{\tilde{r}})$, these trial states satisfy the symmetry constraints and lie in the form domain of ${\tilde{H}}_T^1$. The norm of $\psi _\epsilon$ diverges as $\epsilon \rightarrow 0$.

### Remark 4.2

The trial state is the (anti-)symmetrization of $\Phi _{\lambda }(r)e^{-\epsilon \vert z_1 \vert }$, i.e. the projection of $\Phi _{\lambda }(r)e^{-\epsilon \vert z_1 \vert }$ onto the domain of ${\tilde{H}}_T^{1}$. The intuition behind our choice is that, as we will see in Sect. 6, at weak coupling the Birman–Schwinger operator corresponding to $H_T^{\Omega _1}$ approximately looks like $A^0_T$ (defined in (3.1)) on a restricted domain. This is why we want our trial state to look like the ground state $\Phi _{\lambda }$ of $H_T^{0}$ projected onto the domain of ${\tilde{H}}_T^{1}$.

We shall prove that $\lim _{\epsilon \rightarrow 0} \langle \psi _\epsilon , {\tilde{H}}^1_{T_c^{\Omega _0}(\lambda )} \psi _\epsilon \rangle$ is negative for weak enough coupling. This is the content of the next two Lemmas, which are proved in Sects. 4.2 and 4.3, respectively.

### Lemma 4.3

Let $d\in \{1,2,3\}$, $\mu >0$ and let *V* satisfy Assumption 1.1. Let $\lambda$ be such that $H_{T_c^0(\lambda )}^0$ has a unique ground state $\Phi _\lambda$. Then,

4.5 $$\begin{aligned} \lim _{\epsilon \rightarrow 0} \langle \psi _\epsilon , {\tilde{H}}^1_{T_c^0(\lambda )} \psi _\epsilon \rangle= &   -2\lambda \Bigg (\int _{{\mathbb {R}}^{d+1}} V(r) \Bigg [ -\vert \Phi _\lambda (r)\mp \Phi _\lambda (z_1, {\tilde{r}}) \vert ^2 \chi _{\vert z_1 \vert < \vert r_1 \vert } \nonumber \\  &   +\vert \Phi _\lambda (z_1, {\tilde{r}})\vert ^2 \Bigg ] {\textrm{d}}r {\textrm{d}}z_1 \nonumber \\  &   \mp 2\pi \int _{{\mathbb {R}}^{d-1}} \overline{ \widehat{\Phi }_{\lambda }(0,{\tilde{p}})} \widehat{V\Phi _{\lambda }}(0,{\tilde{p}}) {\textrm{d}}{\tilde{p}}\Bigg ), \end{aligned}$$

where the upper signs correspond to Dirichlet and the lower signs to Neumann boundary conditions. For $d=1$, the last term in (4.5) is to be understood as $\mp 2\pi \overline{ \widehat{\Phi }_{\lambda }(0)} \widehat{V\Phi _{\lambda }}(0)$.

For small $\lambda$ we shall prove that the expression in the round bracket in (4.5) is positive.

### Lemma 4.4

Let $d\in \{1,2,3\}$, $\mu >0$ and let *V* satisfy Assumption 1.1. Let $\lambda _0$ be as in Remark 3.4. Assume Dirichlet or Neumann boundary conditions. For $d=3$ assume that $\int _{{\mathbb {R}}^3} V(r) {\widetilde{m}}_3^{D/N}(r) {\textrm{d}}r>0$, where ${\widetilde{m}}_3^{D/N}$ was defined in (1.6). Then there is a $\lambda _0\ge \lambda _1>0$ such that for $\lambda \le \lambda _1$ the right hand side in (4.5) is negative.

Therefore, for small enough $\epsilon$, $\langle \psi _\epsilon , {\tilde{H}}^1_{T_c^0(\lambda )} \psi _\epsilon \rangle <0$. Since $T_c^{\Omega _0}$ and $T_c^0$ coincide at weak coupling, this proves that $\inf \sigma ({\tilde{H}}_{T_c^{\Omega _0}(\lambda )}^1)<0$ at weak coupling. This concludes the proof of Theorem 1.3.

### Remark 4.5

The additional condition $\int _{{\mathbb {R}}^3} V(r) {\widetilde{m}}_3^{D/N}(r) {\textrm{d}}r>0$ for $d=3$ is exactly the limit of the terms in the round brackets in (4.5) for $\lambda \rightarrow 0$. Taking the limit amounts to replacing $\Phi _\lambda$ by $j_3$ (cf. Lemma 3.6).

### Proof of Lemma 4.1

#### Proof of Lemma 4.1

Let $0<T_0<T_1<\infty$. We claim that there exists a constant $C_{T_0,T_1}$ such that $|K_T(p,q)-K_{T'}(p,q)|\le C_{T_0,T_1} |T-T'|(1+p^2+q^2)$ for all $T_0\le T,T'\le T_1$. To see this, compute

4.6 $$\begin{aligned} \frac{\partial }{\partial T} K_T(p,q)=\frac{K_T(p,q)}{2T^2} \frac{{{\,\textrm{sech}\,}}\left( \frac{p^2-\mu }{2T}\right) ^2(p^2-\mu )+{{\,\textrm{sech}\,}}\left( \frac{q^2-\mu }{2T}\right) ^2(q^2-\mu )}{\tanh \left( \frac{p^2-\mu }{2T}\right) +\tanh \left( \frac{q^2-\mu }{2T}\right) }. \nonumber \\ \end{aligned}$$

$K_T$ can be estimated using Lemma 2.1 and the remaining term is bounded.

The kinetic part $K_T^0$ of $H_T^0$ acts as multiplication by $K_T(p,0)$ in momentum space. For $T_0<T,T'<T_1$ and $\psi$ in the Sobolev space $H^1({\mathbb {R}}^{d})$, therefore

4.7 $$\begin{aligned} \langle \psi , (K_T^{0}-K_{T'}^{0} )\psi \rangle \le C_{T_0,T_1} |T-T'| \Vert \psi \Vert _{H^1({\mathbb {R}}^{d})}. \end{aligned}$$

Similarly, for $T_0<T,T'<T_1$ and $\psi \in H^1({\mathbb {R}}^{2d})$,

4.8 $$\begin{aligned} \langle \psi , (K_T^{{\mathbb {R}}^d}-K_{T'}^{{\mathbb {R}}^d} )\psi \rangle \le C_{T_0,T_1} |T-T'| \Vert \psi \Vert _{H^1({\mathbb {R}}^{2d})}. \end{aligned}$$

Set $D_0:=H^1({\mathbb {R}}^{d})$, $D_{\Omega _0}:=\{\psi \in H^1({\mathbb {R}}^{2d}) \vert \psi (x,y)=\psi (y,x)\}$ and $D_1:=\{\psi \in H^1({\mathbb {R}}^{2d}) \vert \psi (x,y)=\psi (y,x)=\mp \psi ((-x_1,{\tilde{x}}),y)\}$, where $-/+$ corresponds to Dirichlet/Neumann boundary conditions, respectively. Let $j\in \{0,1, \Omega _0\}$ and $\epsilon >0$. There is a family $\{\psi _T \}$ of functions in $D_j$ such that $\Vert \psi _T \Vert _2=1$ and $\langle \psi _T,H_T^j \psi _T \rangle \le \inf \sigma (H_T^j) +\epsilon$.

We first argue that there is a constant $C>0$ such that for all $T\in [T_0,T_1]: \Vert \psi _T \Vert _{H^1}<C$. Recall that 2*T* lies in the essential spectrum of $H_T^0$ restricted to even functions. Together with Lemmas 2.3 and 2.4, $\langle \psi _T,H_T^j \psi _T \rangle \le 2T_1 +\epsilon$. Furthermore, by Lemma 2.1, the kinetic part of $H_T^j$ is bounded below by some constant $C_1(T_0)(1-\Delta )$, where $\Delta$ denotes the Laplacian in all variables. Since the interaction is infinitesimally form bounded with respect to the Laplacian, there is a finite constant $C_2(T_0)$, such that for all $\psi \in D_j$ with $\Vert \psi \Vert _2=1$, $\langle \psi ,H_T^j \psi \rangle \ge \frac{C_1(T_0)}{2}\langle \psi , (1-\Delta )\psi \rangle -C_2(T_0)=\frac{C_1(T_0)}{2} \Vert \psi \Vert _{H^1}-C_2(T_0)$. In particular, $\Vert \psi _T\Vert _{H^1} \le \frac{2}{C_1(T_0)}( 2T_1 +\epsilon +C_2(T_0))=:C$.

Let $$T,T'\in [T_0,T_1]$$. Then

4.9 $$\begin{aligned} \inf \sigma (H_T^j) +\epsilon\ge &   \langle \psi _T,H_T^j \psi _T \rangle =\langle \psi _T,H_{T'}^j \psi _T \rangle +\langle \psi _T, (K_T-K_{T'}) \psi _T \rangle \nonumber \\\ge &   \inf \sigma (H_{T'}^j) -|T-T'| C_{T_0,T_1} C. \end{aligned}$$

Swapping the roles of $T,T'$, we obtain

4.10 $$\begin{aligned}  &   \inf \sigma (H_T^j) -\epsilon - |T-T'| C_{T_0,T_1} C \nonumber \\  &   \quad \le \inf \sigma (H_{T'}^j) \le \inf \sigma (H_T^j) +\epsilon +|T-T'| C_{T_0,T_1} C \end{aligned}$$

and thus

4.11 $$\begin{aligned} \inf \sigma (H_T^j) -\epsilon \le \lim _{T'\rightarrow T} \inf \sigma (H_{T'}^j) \le \inf \sigma (H_T^j) +\epsilon . \end{aligned}$$

Since $\epsilon$ was arbitrary, equality follows. Hence $\inf \sigma (H_T^j)$ is continuous in *T* for $T>0$. $\square$

### Proof of Lemma 4.3

The following technical lemma will be helpful for $d=3$:

#### Lemma 4.6

Let $V,W \in L^1\cap L^{3/2}({\mathbb {R}}^3)$, let *W* be radial and let $\psi \in L^2({\mathbb {R}}^3)$. Then

4.12 $$\begin{aligned}  &   \int _{{\mathbb {R}}^5} \vert \widehat{V^{1/2} \psi }(p) \vert \frac{1}{1+p^2} \widehat{W}(0,{\tilde{p}}-{\tilde{q}}) \frac{1}{1+p_1^2 +{\tilde{q}}^2} \vert \widehat{V^{1/2} \psi } (p_1,{\tilde{q}})\vert {\textrm{d}}p {\textrm{d}}{\tilde{q}}\nonumber \\  &   \quad \le C \Vert \widehat{W}(0,\cdot ) \Vert _{L^3({\mathbb {R}}^2)} \Vert V\Vert _{3/2} \Vert \psi \Vert _2^2<\infty \end{aligned}$$

for some constant *C* independent of *V*, *W* and $\psi$.

#### Proof of Lemma 4.6

By Lemma 3.7(iv), $\widehat{W}(0, \cdot ) \in L^3({\mathbb {R}}^2)\cap L^\infty ({\mathbb {R}}^2)$. By Young’s inequality, the integral is bounded by

4.13 $$\begin{aligned} C \Vert \widehat{W}(0,\cdot ) \Vert _{L^3({\mathbb {R}}^2)} \int _{\mathbb {R}}\left| \int _{{\mathbb {R}}^2} \left| \frac{1}{1+p^2}\widehat{V^{1/2} \psi }(p) \right| ^{6/5} {\textrm{d}}{\tilde{p}} \right| ^{5/3} {\textrm{d}}p_1. \end{aligned}$$

By Lemma 3.7(iii), $\Vert \widehat{V^{1/2} \psi }\Vert _6 \le C \Vert V \Vert _{3/2}^{1/2} \Vert \psi \Vert _2$. Applying Hölder’s inequality in the ${\tilde{p}}$ variables, we obtain the bound

4.14 $$\begin{aligned} C \Vert \widehat{W}(0,\cdot ) \Vert _{L^3({\mathbb {R}}^2)} \int _{\mathbb {R}}\left| \int _{{\mathbb {R}}^2} \frac{1}{(1+p^2)^{3/2}}{\textrm{d}}{\tilde{p}} \right| ^{4/3} \left| \int _{{\mathbb {R}}^2} |\widehat{V^{1/2} \psi }(p)|^6{\textrm{d}}{\tilde{p}} \right| ^{1/3}{\textrm{d}}p_1. \nonumber \\ \end{aligned}$$

Applying Hölder’s inequality in $p_1$, we further obtain

4.15 $$\begin{aligned} C \Vert \widehat{W}(0,\cdot ) \Vert _{L^3({\mathbb {R}}^2)} \left( \int _{\mathbb {R}}\left| \int _{{\mathbb {R}}^2} \frac{1}{(1+p^2)^{3/2}}{\textrm{d}}{\tilde{p}} \right| ^{2} {\textrm{d}}p_1\right) ^{2/3} \Vert \widehat{V^{1/2}\psi }\Vert _6^2. \end{aligned}$$

The remaining integral is finite. $\square$

#### Proof of Lemma 4.3

Plugging in the trial state and regrouping terms we obtain

4.16 $$\begin{aligned}  &   \langle \psi _\epsilon , {\tilde{H}}^1_{T_c^0(\lambda )} \psi _\epsilon \rangle = 2\int _{{\mathbb {R}}^{2d+2}} \Bigg [ \overline{\Phi _{\lambda }(r)}e^{-\epsilon \vert z_1 \vert }(K_{T}(r,z_1;r',z_1') \nonumber \\  &   \quad -\lambda V(r)\delta (r-r' )\delta (z_1-z_1'))\Phi _{\lambda }(r')e^{-\epsilon \vert z_1' \vert } \nonumber \\  &   \quad \mp \overline{\Phi _{\lambda }(r)}e^{-\epsilon \vert z_1 \vert }(K_{T}(r,z_1;r',z_1') \nonumber \\  &   \quad -\lambda V(r) \delta (r-r' )\delta (z_1-z_1'))e^{-\epsilon \vert r_1' \vert }\Phi _{\lambda }(z_1',{\tilde{r}}')\Bigg ] {\text {d}}r{\text {d}}z_1 {\text {d}}r' {\text {d}}z_1' \nonumber \\  &   \quad +2\int _{{\mathbb {R}}^{d+1}} \Bigg [ \lambda V(r)\chi _{\vert z_1 \vert<\vert r_1 \vert }|\Phi _{\lambda }(r)|^2 e^{-2\epsilon \vert z_1 \vert } \nonumber \\  &   \quad \mp \overline{\Phi _{\lambda }(r)}e^{-\epsilon \vert z_1 \vert } \lambda V(r) \chi _{\vert z_1 \vert<\vert r_1 \vert }e^{-\epsilon \vert r_1 \vert }\Phi _{\lambda }(z_1,{\tilde{r}}) \nonumber \\  &   \quad + \lambda V(z_1, {\tilde{r}})\chi _{\vert r_1 \vert <\vert z_1 \vert }|\Phi _{\lambda }(r)|^2 e^{-2\epsilon \vert z_1 \vert } \nonumber \\  &   \quad \mp \overline{\Phi _{\lambda }(r)}e^{-\epsilon \vert z_1 \vert } \lambda V(z_1, {\tilde{r}})\chi _{\vert z_1 \vert >\vert r_1 \vert }e^{-\epsilon \vert r_1 \vert }\Phi _{\lambda }(z_1,{\tilde{r}}) \nonumber \\  &   \quad - \lambda V(z_1, {\tilde{r}})|\Phi _{\lambda }(r)|^2 e^{-2\epsilon \vert z_1 \vert } \nonumber \\  &   \quad \pm \overline{\Phi _{\lambda }(r)}e^{-\epsilon \vert z_1 \vert } \lambda V(z_1, {\tilde{r}})e^{-\epsilon \vert r_1 \vert }\Phi _{\lambda }(z_1,{\tilde{r}}) \Bigg ] {\text {d}}r{\text {d}}z_1. \end{aligned}$$

We will prove that the first integral vanishes due to the eigenvalue equation $H^0_{T_c^0(\lambda )}\Phi _\lambda =0$ as $\epsilon \rightarrow 0$. For the second integral in (4.16), we will show that it is bounded as $\epsilon \rightarrow 0$ and argue that it is possible to interchange limit and integration. The limit of the second integral is exactly the right hand side of (4.5).

The first two terms in the integrand of the second integral in (4.16) can be bounded by $\lambda \Vert \Phi _{\lambda }\Vert _\infty ^2 |V(r)|\chi _{|z_1| <| r_1 |}$. This is an $L^1$ function, since $\vert \cdot \vert V\in L^1$ and $\Vert \Phi _{\lambda }\Vert _\infty < \infty$ by Lemma 3.5. The same argument applies to the next two terms as well.

For the fifth term in the second integral, we can interchange limit and integration by dominated convergence if $\int _{{\mathbb {R}}^{d+1}} \vert V(r)\vert \vert \Phi _{\lambda }(z_1, {\tilde{r}}) \vert ^2 {\textrm{d}}r {\textrm{d}}z_1<\infty$. Observe that

4.17 $$\begin{aligned}  &   \int _{{\mathbb {R}}^{d+1}} \vert V(r)\vert \vert \Phi _{\lambda }(z_1, {\tilde{r}}) \vert ^2 {\textrm{d}}r {\textrm{d}}z_1 \nonumber \\    &   \qquad = (2\pi )^{1-d/2} \int _{{\mathbb {R}}^{2d-1}} \overline{\widehat{\Phi }_{\lambda }(p)}\widehat{\vert V\vert }(0,{\tilde{p}} - {\tilde{q}}) \widehat{\Phi }_{\lambda }(p_1,{\tilde{q}}) {\textrm{d}}p {\textrm{d}}{\tilde{q}}. \end{aligned}$$

According to Lemma 3.5(i) the latter is bounded by

4.18 $$\begin{aligned}  &   C \int _{{\mathbb {R}}^{2d-1}}\vert \widehat{V^{1/2} \Psi _{T_c^0(\lambda )}}(p) \vert \frac{1}{1+p^2} \widehat{\vert V\vert }(0,{\tilde{p}}-{\tilde{q}})\nonumber \\  &   \quad \times \frac{1}{1+p_1^2 +{\tilde{q}}^2} \vert \widehat{V^{1/2} \Psi _{T_c^0(\lambda )}} (p_1,{\tilde{q}})\vert {\textrm{d}}p {\textrm{d}}{\tilde{q}}. \end{aligned}$$

For $d=1,2$ we bound this by

4.19 $$\begin{aligned} C \Vert V \Vert _1^2 \Vert \Psi \Vert _2^2 \int _{{\mathbb {R}}^{2d-1}} \frac{1}{(1+p_1^2+{\tilde{p}}^2)(1+p_1^2+{\tilde{q}}^2)} {\textrm{d}}p {\textrm{d}}{\tilde{q}}, \end{aligned}$$

which is finite. For $d=3$, (4.19) is finite by Lemma 4.6 since $W=|V|$ is radial and in $L^1\cap L^{3/2}$. Hence, limit and integration can be interchanged for the fifth term in the second integral in (4.16).

For the last term in (4.16) we have

4.20 $$\begin{aligned}  &   \int _{{\mathbb {R}}^{d+1}} \overline{\Phi _{\lambda }(r)}e^{-\epsilon \vert z_1 \vert } V(z_1,{\tilde{r}})e^{-\epsilon \vert r_1 \vert }\Phi _{\lambda }(z_1,{\tilde{r}}) {\textrm{d}}z_1 {\textrm{d}}r\nonumber \\  &   \quad =\frac{2}{\pi }\int _{{\mathbb {R}}^{d+1}} \overline{ \widehat{\Phi }_{\lambda }(p)} \frac{\epsilon }{\epsilon ^2+q_1^2} \frac{\epsilon }{\epsilon ^2+p_1^2} \widehat{V\Phi _{\lambda }}(q_1,{\tilde{p}}) {\textrm{d}}p {\textrm{d}}q_1\nonumber \\  &   \quad =\frac{2}{\pi }\int _{{\mathbb {R}}^{d+1}} \overline{ \widehat{\Phi }_{\lambda }(\epsilon p_1,{\tilde{p}})} \frac{1}{1+q_1^2} \frac{1}{1+p_1^2} \widehat{V\Phi _{\lambda }}(\epsilon q_1,{\tilde{p}}) {\textrm{d}}p {\textrm{d}}q_1. \end{aligned}$$

According to Lemma 3.5(i) and Lemma 3.7(iii), the integrand is bounded by $\frac{C(\lambda ) }{1+{\tilde{p}}^2} \frac{ \Vert V\Vert _1 \Vert \Psi \Vert _2^2}{(1+q_1^2)(1+p_1^2)}$. For $d=1,2$ this is integrable, so by dominated convergence and since $\int _{\mathbb {R}}\frac{1}{1+x^2} {\textrm{d}}x =\pi$, this term converges to the last term in (4.5). For $d=3$, the following result will be useful:

#### Lemma 4.7

Let $\lambda ,T,\mu >0$ and $d=3$ and let *V* satisfy 1.1. The functions

4.21 $$\begin{aligned} f(p_1,q_1)=\int _{{\mathbb {R}}^{2}}\overline{ \widehat{\Phi }_{\lambda }(p)} \widehat{V\Phi _{\lambda }}(q_1,{\tilde{p}}) {\textrm{d}}{\tilde{p}} \end{aligned}$$

and

4.22 $$\begin{aligned} g(p_1,q_1)= \int _{{\mathbb {R}}^{2}} B^{-1}_{T}(( p_1, {\tilde{p}}),( q_1,{\tilde{0}})) \overline{\widehat{\Phi }_{\lambda }( p_1, {\tilde{p}})} \widehat{\Phi }_{\lambda }( q_1,{\tilde{p}}) {\textrm{d}}{\tilde{p}} \end{aligned}$$

are bounded and continuous.

Its proof can be found after the end of the current proof.

We write the term in (4.20) as

4.23 $$\begin{aligned} \frac{2}{\pi }\int _{{\mathbb {R}}^{2}} \frac{f(\epsilon p_1,\epsilon q_1)}{(1+q_1^2)(1+p_1^2)}{\textrm{d}}p_1 {\textrm{d}}q_1. \end{aligned}$$

By Lemma 4.7 we can exchange limit and integration by dominated convergence and (4.23) converges to the last term in (4.5).

For the second summand in the first integral in (4.16) we also want to argue using dominated convergence. The interaction term agrees with (4.20). The kinetic term can be written as

4.24 $$\begin{aligned}  &   \frac{4}{\pi } \int _{{\mathbb {R}}^{d+1}} \frac{1}{(1+q_1^2)(1+p_1^2)}B^{-1}_{T}((\epsilon p_1, {\tilde{p}}),(\epsilon q_1,{\tilde{0}})) \overline{\widehat{\Phi }_{\lambda }(\epsilon p_1, {\tilde{p}}) } \widehat{\Phi }_{\lambda }(\epsilon q_1,{\tilde{p}}) {\textrm{d}}p {\textrm{d}}q_1 \nonumber \\  &   \quad =\frac{4}{\pi } \int _{{\mathbb {R}}^{2}} \frac{1}{(1+q_1^2)(1+p_1^2)} g(\epsilon p_1,\epsilon q_1) {\textrm{d}}p_1 {\textrm{d}}q_1. \end{aligned}$$

For $d=3$, we can apply dominated convergence according to Lemma 4.7. For $d=1,2$ note that by Lemma 3.5 and Lemma 2.1,

4.25 $$\begin{aligned} B^{-1}_{T}(p,(q_1,{\tilde{0}})) \vert \widehat{\Phi }_{\lambda }(p) \vert \vert \widehat{\Phi }_{\lambda }(q_1, {\tilde{p}})\vert\le &   C_{T,\mu ,\lambda }\frac{1+p^2+q_1^2}{(1+p^2)(1+q_1^2+{\tilde{p}}^2)}\Vert V\Vert _1 \Vert \Psi \Vert _2^2 \nonumber \\\le &   2 C_{T,\mu ,\lambda }\frac{\Vert V\Vert _1 \Vert \Psi \Vert _2^2 }{1+{\tilde{p}}^2}. \end{aligned}$$

Therefore, the integrand is bounded by $\frac{C \Vert V\Vert _1 \Vert \Psi \Vert _2^2 }{(1+q_1^2)(1+p_1^2)(1+{\tilde{p}}^2)}$. For $d=1,2$ this is integrable and we can apply dominated convergence. We conclude that the limit of the second summand in the first integral in (4.16) as $\epsilon \rightarrow 0$ equals

4.26 $$\begin{aligned} 4 \pi \int _{{\mathbb {R}}^{d-1}}\left( \frac{\vert \widehat{\Phi }_{\lambda }(0,{\tilde{p}}) \vert ^2}{B_{T}((0,{\tilde{p}}),0)}-\lambda \overline{ \widehat{\Phi }_{\lambda }(0,{\tilde{p}})} \widehat{ V \Phi _{\lambda }}(0,{\tilde{p}}) \right) {\textrm{d}}{\tilde{p}}= 0, \end{aligned}$$

where we used that $\int _{\mathbb {R}}\frac{1}{1+x^2} {\textrm{d}}x =\pi$ and (3.22).

To see that the first summand in the first integral in (4.16) vanishes as $\epsilon \rightarrow 0$, we use (3.22) to obtain

4.27 $$\begin{aligned}  &   \frac{2}{\epsilon }\lambda \int _{{\mathbb {R}}^d} V(r) \vert \Phi _{\lambda }(r)\vert ^2{\textrm{d}}r = \frac{2}{\epsilon } \int _{{\mathbb {R}}^d} B_{T}^{-1}(p,0)\vert \widehat{\Phi }_{\lambda }(p)\vert ^2 {\textrm{d}}p \nonumber \\  &   \quad = \frac{4}{\pi } \int _{{\mathbb {R}}^{d+1}} \frac{\epsilon ^2}{(\epsilon ^2+q_1^2)^2} B_{T}^{-1}(p,0)\vert \widehat{\Phi }_{\lambda }(p)\vert ^2 {\textrm{d}}p {\textrm{d}}q_1. \end{aligned}$$

Hence, we need to prove that

4.28 $$\begin{aligned} \lim _{\epsilon \rightarrow 0} \int _{{\mathbb {R}}^{d+1}} \frac{\epsilon ^2}{(\epsilon ^2+q_1^2)^2} (B^{-1}_{T}(p,(q_1, {\tilde{0}}))- B^{-1}_{T}(p,0))\vert \widehat{\Phi }_{\lambda }(p) \vert ^2 {\textrm{d}}p {\textrm{d}}q_1=0. \nonumber \\ \end{aligned}$$

We split the integration into two regions with $\vert q_1 \vert >C_1$ and $\vert q_1 \vert <C_1$, respectively. By Lemma 2.1, we have $B^{-1}_{T}(p,q)\le C_2 (1+p^2+q^2)$. Together with $\Phi _{\lambda } \in H^1({\mathbb {R}}^d)$, we therefore have that

4.29 $$\begin{aligned}  &   \int _{{\mathbb {R}}^{d+1}, \vert q_1 \vert>C_1}\frac{\epsilon ^2}{(\epsilon ^2+q_1^2)^2} \vert B^{-1}_{T}(p,(q_1,{\tilde{0}}))- B^{-1}_{T}(p,0)\vert \vert \widehat{\Phi }_{\lambda }(p) \vert ^2 {\textrm{d}}p {\textrm{d}}q_1\nonumber \\  &   \quad \le 2C_2 \int _{{\mathbb {R}}^2, \vert q _1\vert >C_1} \frac{\epsilon ^2(1+p^2+q_1^2)\vert \widehat{\Phi }_{\lambda }(p) \vert ^2 }{q_1^4} {\textrm{d}}p {\textrm{d}}q_1 < C_3 \epsilon ^2 \Vert \Phi _\lambda \Vert _{H^1}^2, \nonumber \\ \end{aligned}$$

which vanishes in the limit $\epsilon \rightarrow 0$. For the case $\vert q_1 \vert <C$, the next lemma is useful: its proof can be found at the end of this Section.

#### Lemma 4.8

Let $T,\mu >0$, $d\in \{1,2,3\}$. The function

4.30 $$\begin{aligned} k(p,q):=\frac{1}{\vert q\vert }(B_{T}(p,q)- B_{T}(p,0)) \end{aligned}$$

is continuous at $q=0$ and satisfies $k(p,0)=0$ for all $p\in {\mathbb {R}}^d$. Furthermore, there is a constant *C* depending only on $T,\mu ,d$ such that $\vert k(p,q) \vert < \frac{C}{1+p^2}$ for all $p,q\in {\mathbb {R}}^d$.

Since $B^{-1}_{T}(p,q)- B^{-1}_{T}(p,0)= -\frac{ \vert q \vert k(p,q)}{B_{T}(p,q) B_{T}(p,0)}$, we have

4.31 $$\begin{aligned}  &   \int _{{\mathbb {R}}^{d+1}, \vert q_1 \vert<C_1}\frac{\epsilon ^2}{(\epsilon ^2+q_1^2)^2} ( B^{-1}_{T}(p,(q_1,{\tilde{0}}))- B^{-1}_{T}(p,0)) \vert \widehat{\Phi }_{\lambda }(p) \vert ^2 {\textrm{d}}p {\textrm{d}}q_1\nonumber \\  &   \quad =-\int _{{\mathbb {R}}^{d+1}}\frac{ \vert q_1 \vert \chi _{\vert q_1 \vert <C_1/\epsilon }}{(1+q_1^2)^2} \frac{ k(p,(\epsilon q_1,{\tilde{0}}))}{B_{T}(p,(\epsilon q_1,{\tilde{0}})) B_{T}(p,0)} \vert \widehat{\Phi }_{\lambda }(p) \vert ^2 {\textrm{d}}p {\textrm{d}}q_1 \nonumber \\ \end{aligned}$$

By Lemma 2.1 and Lemma 4.8, we can bound the absolute value of the integrand by

4.32 $$\begin{aligned} C \frac{ \vert q_1 \vert \chi _{\vert q_1 \vert <C_1/\epsilon }}{(1+q_1^2)^2} (1+p^2+\epsilon ^2 q_1^2) \vert \widehat{\Phi }_{\lambda }(p) \vert ^2 \le C \frac{ \vert q_1 \vert }{(1+q_1^2)^2} (1+p^2+C_1^2)\vert \widehat{\Phi }_{\lambda }(p) \vert ^2 \end{aligned}$$

The latter is integrable since $\Phi _\lambda \in H^1({\mathbb {R}}^d)$. Thus, by dominated convergence and since $k(p,0)=0$, the integral vanishes in the limit $\epsilon \rightarrow 0$.$\square$

#### Proof of Lemma 4.7

For convenience, we introduce the notation $D_f(p,q_1)=\lambda B_{T}(p,0)$ and

4.33 $$\begin{aligned} D_g(p,q_1)=\lambda ^2 B_{T}(p,0) B_{T}(p,(q_1,\tilde{0}))^{-1}B_{T}((q_1,{\tilde{p}}),0). \end{aligned}$$

For $h\in \{f,g\}$, $D_f(p,q_1),D_g(p,q_1)\le \frac{C}{1+{\tilde{p}}^2}$ by Lemma 2.1 and (2.3). Furthermore,

4.34 $$\begin{aligned} h(p_1,q_1)=\int _{{\mathbb {R}}^{d-1}}\overline{ \widehat{V\Phi _{\lambda }}(p_1,{\tilde{p}}) }D_h(p,q_1) \widehat{V\Phi _{\lambda }}(q_1,{\tilde{p}}) {\textrm{d}}{\tilde{p}}, \end{aligned}$$

using (3.22).

#### Lemma 4.9

For $$h\in \{f,g\}$$,

4.35 $$\begin{aligned} \sup _{p_1,q_1,w_1 \in {\mathbb {R}}} \Vert D_h((p_1,\cdot ),q_1)\widehat{V\Phi _{\lambda }}(w_1,\cdot )\Vert _{L^1({\mathbb {R}}^2)}\!\le &   \!\sup _{w_1 \in {\mathbb {R}}} \left\Vert \frac{C}{1{+}\vert \cdot \vert ^2}\widehat{V\Phi _{\lambda }}(w_1,\cdot )\right\Vert _{L^1({\mathbb {R}}^2)}\nonumber \\< &   \infty . \end{aligned}$$

#### Proof

Using Hölder’s inequality,

4.36 $$\begin{aligned}  &   \Vert D_h((p_1,\cdot ),q_1)\widehat{V\Phi _{\lambda }}(w_1,\cdot )\Vert _{L^1({\mathbb {R}}^2)}\nonumber \\  &   \quad \le \left\Vert \frac{C}{1+\vert \cdot \vert ^2}\widehat{V\Phi _{\lambda }}(w_1,\cdot )\right\Vert _{L^1({\mathbb {R}}^2)}\nonumber \\  &   \quad \le \frac{1}{(2\pi )^{3/2}}\int _{{\mathbb {R}}^{2}}\int _{{\mathbb {R}}^3}\frac{C}{1+{\tilde{p}}^2} \vert \widehat{V}((w_1,{\tilde{p}})-k)\vert \vert \widehat{\Phi _{\lambda }}(k)\vert {\textrm{d}}k {\textrm{d}}{\tilde{p}} \nonumber \\  &   \quad \le C \left\| \frac{1}{1+\vert \cdot \vert ^2} \right\| _{L^r({\mathbb {R}}^{2})} \int _{\mathbb {R}}\left( \int _{{\mathbb {R}}^2}\vert \widehat{V}(w_1-k_1,{\tilde{p}})\vert ^s {\textrm{d}}{\tilde{p}}\right) ^{1/s} \left( \int _{{\mathbb {R}}^2}\vert \widehat{\Phi _{\lambda }}(k)\vert {\textrm{d}}{\tilde{k}}\right) {\textrm{d}}k_1 \nonumber \\  &   \quad \le C \left\| \frac{1}{1+\vert \cdot \vert ^2} \right\| _{L^r({\mathbb {R}}^{2})} \sup _{k_1} \Vert \widehat{V}(k_1,\cdot )\Vert _s \Vert \widehat{\Phi }_{\lambda }\Vert _1, \end{aligned}$$

where $1=1/r+1/s$. For this to be finite we need $r>1$, i.e. $s<\infty$. By Lemma 3.7(iv), $\sup _{q_1} \Vert \widehat{V}(q_1,\cdot )\Vert _3 <\infty$. Furthermore $\Vert \widehat{\Phi }_{\lambda } \Vert _1$ is bounded by Lemma 3.5. $\square$

The functions *f* and *g* are bounded, as can be seen using that $\Vert \widehat{V \Phi _{\lambda }}\Vert _\infty \le C \Vert V\Vert _1^{1/2} \Vert \Psi _{T_c^0(\lambda )} \Vert _2$ by Lemma 3.7(iii) and $\Vert \Psi _{T_c^0(\lambda )} \Vert _2=1$, hence we get that, for $h\in \{f,g\}$,

4.37 $$\begin{aligned} |h(p_1,q_1)|\le C \Vert V\Vert _1^{1/2} \sup _{p_1,q_1} \Vert D_h((p_1,\cdot ),q_1)\widehat{V\Phi _{\lambda }}(q_1,\cdot )\Vert _{L^1({\mathbb {R}}^2)}, \end{aligned}$$

which is finite by Lemma 4.9. To see continuity, we write, for $h\in \{f,g\}$,

4.38 $$\begin{aligned}&|h(p_1+\epsilon _1,q_1+\epsilon _2)-h(p_1,q_1)|\nonumber \\&\quad \le \Bigg | \int _{{\mathbb {R}}^2}\overline{(\widehat{V\Phi _{\lambda }}(p_1{+}\epsilon _1,{\tilde{p}}){-} \widehat{V \phi _{T,\lambda }}(p))} D_h((p_1{+}\epsilon _1,{\tilde{p}}),q_1{+}\epsilon _2) \widehat{V\Phi _{\lambda }}(q_1{+}\epsilon _2,{\tilde{p}}){\textrm{d}}{\tilde{p}}\Bigg |\nonumber \\&\qquad + \Bigg |\int _{{\mathbb {R}}^2}\overline{ \widehat{V\Phi _{\lambda }}(p)} D_h((p_1+\epsilon _1,{\tilde{p}}),q_1+\epsilon _2) (\widehat{V\Phi _{\lambda }}(q_1+\epsilon _2,{\tilde{p}})-\widehat{V\Phi _{\lambda }}(q_1,{\tilde{p}})) {\textrm{d}}{\tilde{p}} {\textrm{d}}k \Bigg |\nonumber \\&\qquad + \Bigg |\int _{{\mathbb {R}}^2}\overline{ \widehat{V \Phi _{\lambda }}(p)} (D_h((p_1+\epsilon _1,{\tilde{p}}),q_1+\epsilon _2)-D_h(p,q_1))\widehat{V\Phi _{\lambda }}(q_1,{\tilde{p}}) {\textrm{d}}{\tilde{p}}\Bigg |. \end{aligned}$$

Observe that

4.39 $$\begin{aligned} |\widehat{V \Phi _{\lambda }}(p_1+\epsilon _1,{\tilde{p}})- \widehat{V \Phi _{\lambda }}(p)|\le &   \frac{1}{(2\pi )^{d/2}}\int _{{\mathbb {R}}^d} | e^{i \epsilon _1 r_1}-1| |V(r)||\Phi _{\lambda }(r)| {\textrm{d}}r\nonumber \\\le &   \frac{\epsilon _1 \Vert \Phi _{\lambda }\Vert _\infty \Vert |\cdot | V \Vert _1}{(2\pi )^{d/2}}. \end{aligned}$$

With Lemmas 4.9 and 3.5, we bound the first two terms in (4.38) by $C \epsilon _1$ and $C\epsilon _2$, respectively. Hence they vanish as $\epsilon _1,\epsilon _2\rightarrow 0$. The absolute value of the integrand in the last term in (4.38) is bounded by $\Vert \widehat{V \Phi _{\lambda }}\Vert _\infty \frac{2C}{1+{\tilde{p}}^2} \widehat{V\Phi _{\lambda }}(q_1,{\tilde{p}})$. By Lemma 4.9, this is an $L^1$ function. Hence, when taking the limit $\epsilon _1,\epsilon _2\rightarrow 0$, we are allowed to pull the limit into the integral by dominated convergence, showing that also the last term vanishes. Therefore, the functions *f* and *g* are continuous.$\square$

#### Proof of Lemma 4.8

This Lemma is a generalization of [6, Lemma 3.2] and its proof follows the same ideas. For $\vert q \vert >1$, Lemma 2.1 implies the bound $\vert k(p,q) \vert < \frac{C}{1+p^2}$. For $\vert q \vert <1$, we use the partial fraction expansion (see [6, (2.2)])

4.40 $$\begin{aligned} k(p,q)=2T\sum _{n\in {\mathbb {Z}}} \frac{\vert q \vert (2\mu -q^2-2p^2+4 (p \cdot \frac{q}{\vert q \vert })^2)- 4 i w_n p \cdot \frac{q}{\vert q \vert }}{\left( \left( p+q\right) ^2-\mu -iw_n\right) \left( \left( p-q\right) ^2-\mu +iw_n\right) \left( p^2-\mu -iw_n\right) \left( p^2-\mu +iw_n\right) }, \end{aligned}$$

where $w_n=(2n+1)\pi T$. Continuity of *k* follows e.g. using the Weierstrass M-test. Noting that $w_n=-w_{-n-1}$, it is easy to see that $k(p,0)=0$.

With the estimates

4.41 $$\begin{aligned}&\sup _{(p,q)\in {\mathbb {R}}^{2d}, \vert q \vert<1} \left| \frac{\vert q \vert (2\mu -q^2-2p^2+4 (p \cdot \frac{q}{\vert q \vert })^2)}{ \left( \left( p+q\right) ^2-\mu -iw_n\right) \left( \left( p-q\right) ^2-\mu +iw_n\right) }\right| \nonumber \\&\quad \le \sup _{(p,q)\in {\mathbb {R}}^{2d}, \vert q \vert<1} \frac{ \vert q \vert (2\mu +q^2+6p^2) }{\sqrt{\left[ \left( p+q\right) ^2-\mu \right] ^2+w_0^2}\sqrt{\left[ \left( p-q\right) ^2-\mu \right] ^2+w_0^2}}=:c_1 <\infty \end{aligned}$$

and

4.42 $$\begin{aligned}  &   \sup _{(p,q)\in {\mathbb {R}}^{2d}, \vert q \vert<1}\left| \frac{4 i w_n p}{\left( \left( p+q\right) ^2-\mu -iw_n\right) \left( \left( p-q\right) ^2-\mu +iw_n\right) }\right| \nonumber \\  &   \quad \le \sup _{(p,q)\in {\mathbb {R}}^{2d}, \vert q \vert<1} \frac{ 4\vert p\vert }{\sqrt{\left[ \left( p + q\right) ^2-\mu \right] ^2+w_0^2}}=:c_2 <\infty \end{aligned}$$

one obtains

4.43 $$\begin{aligned} \vert k(p,q) \vert \le 2T(c_1+c_2)\sum _{n\in {\mathbb {Z}}} \frac{1}{\left( p^2-\mu \right) ^2+w_n^2}. \end{aligned}$$

Using that the summands are decreasing in *n*, we can estimate the sum by an integral

4.44 $$\begin{aligned} \vert k(p,q) \vert\le &   4T(c_1+c_2)\left[ \frac{1}{\left( p^2-\mu \right) ^2+w_0^2} +\int _{1/2}^\infty \frac{1}{\left( p^2- \mu \right) ^2+4\pi ^2T^2 x^2} {\textrm{d}}x \right] \nonumber \\= &   4T(c_1+c_2) \left[ \frac{1}{\left( p^2-\mu \right) ^2+w_0^2} +\frac{\arctan \left( \frac{\vert p^2- \mu \vert }{\pi T}\right) }{2\pi T \vert p^2- \mu \vert }\right] \nonumber \\< &   C \frac{1}{1+p^2} \end{aligned}$$

for some constant *C* independent of *p* and *q*. $\square$

### Proof of Lemma 4.4

#### Proof of Lemma 4.4

Recall that $\Psi _{T_c^0(\lambda )} =V^{1/2}\Phi _\lambda$ with normalization $\Vert \Psi _{T_c^0(\lambda )} \Vert _2^2=\Vert \Psi \Vert _2^2=\int _{{\mathbb {R}}^d} V(r)j_d(r)^2 {\textrm{d}}r$, where $j_d$ was defined in (1.5). Recall from (4.5) that

4.45 $$\begin{aligned}  &   -\frac{1}{2\lambda } \lim _{\epsilon \rightarrow 0} \langle \psi _\epsilon , H^1_{T_c^0(\lambda ),\lambda } \psi _\epsilon \rangle \nonumber \\  &   = \int _{{\mathbb {R}}^{d+1}} V(r) \vert \Phi _\lambda (z_1, {\tilde{r}})\vert ^2 {\text {d}}r {\text {d}}z_1 \nonumber \\  &   - \int _{{\mathbb {R}}^{d+1}} V(r)\vert \Phi _\lambda (z_1, {\tilde{r}})\mp \Phi _\lambda (r) \vert ^2 \chi _{\vert z_1 \vert < \vert r_1 \vert }{\text {d}}r {\text {d}}z_1 \nonumber \\  &   \mp 2\pi \int _{{\mathbb {R}}^{d-1}} \overline{ \widehat{\Phi }_{\lambda }(0,{\tilde{p}})} \widehat{V\Phi _{\lambda }}(0,{\tilde{p}}) {\text {d}}{\tilde{p}}. \end{aligned}$$

The claim follows, if we prove that the right hand side is positive in the limit $\lambda \rightarrow 0$. For $d\in \{1,2\}$ we prove that the second and third term are bounded and the first term diverges as $\lambda \rightarrow 0$. For $d=3$ the first term is bounded too, so we need to compute the limit of all terms. The idea is that in the limit, one would like to replace $\Phi _\lambda$ by $j_3$ using Lemmas 3.3 and 3.6. We consider each of the three summands in (4.45) separately.

*Second term:* The second term is bounded by $4\Vert |\cdot | V \Vert _1 \Vert \Phi _\lambda \Vert _\infty ^2$, which is bounded for small $\lambda$ by Lemma 3.5. For $d=3$ we want to compute the limit. By Lemma 3.6 the integrand is bounded by $8 |V(r)| \Vert j_3 \Vert _\infty ^2 \chi _{|z_1|<|r_1|}$ for $\lambda$ small enough, which is integrable. By dominated convergence, the term thus converges to

4.46 $$\begin{aligned} -\int _{{\mathbb {R}}^4} V(r) \vert j_3(z_1,{\tilde{r}}) \mp j_3(r) \vert ^2 \chi _{|z_1|<|r_1|} {\textrm{d}}r {\textrm{d}}z_1. \end{aligned}$$

*Third term:* Using (3.22) the third term in (4.45) equals

4.47 $$\begin{aligned} \mp 2\pi \lambda \int _{{\mathbb {R}}^{d-1}} \vert \widehat{V^{1/2} \Psi _{T_c^0(\lambda )} }(0,{\tilde{p}})\vert ^2 B_{T_c^0(\lambda )} ((0,{\tilde{p}} ),0) {\textrm{d}}{\tilde{p}} \end{aligned}$$

For $d=1$, this is bounded by $2\pi \lambda B_{T_c^0(\lambda ) } (0,0) \Vert \widehat{V^{1/2} \Psi _{T_c^0(\lambda ) } }\Vert _\infty ^2$. By Lemma 3.7(iii) and since $\sup _T B_{T}(0,0)=\frac{1}{\mu }$, this is $O(\lambda )$ as $\lambda \rightarrow 0$. For $d=2$ we use (2.3) to bound (4.47) by

4.48 $$\begin{aligned}  &   2\pi \lambda \int _{\vert {\tilde{p}} \vert ^2 <2\mu } B_{T_c^0(\lambda )}((0,{\tilde{p}} ),0) {\textrm{d}}{\tilde{p}} \Vert \widehat{V^{1/2} \Psi _{T_c^0(\lambda )} }\Vert _\infty ^2\nonumber \\  &   \quad +C\lambda \int _{\vert {\tilde{p}} \vert ^2 >2\mu }\frac{1}{1+{\tilde{p}}^2}{\textrm{d}}{\tilde{p}} \Vert \widehat{V^{1/2} \Psi _{T_c^0(\lambda ) }}\Vert _\infty ^2, \end{aligned}$$

where *C* is independent of $\lambda$. By Lemma 3.7(iii) $\Vert \widehat{V^{1/2} \Psi _{T_c^0(\lambda )} }\Vert _\infty$ is bounded as $\lambda \rightarrow 0$. The second term in (4.48) thus vanishes as $\lambda \rightarrow 0$. For the first term, recall from (3.2) that $\int _{\vert {\tilde{p}} \vert ^2 <2\mu } B_{T_c^0,\mu }((0,{\tilde{p}} ),0) {\textrm{d}}{\tilde{p}} = 2 \pi m_\mu ^{d=2}(T_c^0(\lambda ))$. By Lemma 3.2 the first term is bounded for small $\lambda$. For $d=3$, we rewrite (4.47) as

4.49 $$\begin{aligned}  &   \mp 2\pi \lambda \int _{{\tilde{p}}^2>2\mu } \vert \widehat{V^{1/2} \Psi _{T_c^0(\lambda )}}(0,{\tilde{p}})\vert ^2 B_{T_c^0} ((0,{\tilde{p}} ),0) {\textrm{d}}{\tilde{p}}\nonumber \\  &   \quad \mp \lambda \int _{{\tilde{p}}^2<2\mu }\int _{{\mathbb {R}}^6} \overline{V^{1/2}\Psi _{T_c^0(\lambda )}(x) } \frac{e^{i{\tilde{p}}\cdot ({\tilde{x}}-{\tilde{y}})}-e^{i\sqrt{\mu } \frac{{\tilde{p}}}{|{\tilde{p}}|}\cdot ({\tilde{x}}-{\tilde{y}})}}{(2\pi )^2}\nonumber \\  &   \qquad \times B_{T_c^0}((0,{\tilde{p}} ),0)V^{1/2} \Psi _{T_c^0(\lambda )}(y){\textrm{d}}x {\textrm{d}}y{\textrm{d}}{\tilde{p}}\nonumber \\  &   \quad \mp \lambda \int _{{\tilde{p}}^2<2\mu }\int _{{\mathbb {R}}^6} \overline{\left( V^{1/2}\Psi _{T_c^0(\lambda )}(x)-Vj_3(x) \right) } \frac{e^{i\sqrt{\mu } \frac{{\tilde{p}}}{|{\tilde{p}}|}\cdot ({\tilde{x}}-{\tilde{y}})}}{(2\pi )^2}\nonumber \\  &   \qquad \times B_{T_c^0}((0,{\tilde{p}} ),0) V^{1/2} \Psi _{T_c^0(\lambda )} (y){\textrm{d}}x {\textrm{d}}y{\textrm{d}}{\tilde{p}}\nonumber \\  &   \quad \mp \lambda \int _{{\tilde{p}}^2<2\mu }\int _{{\mathbb {R}}^6} V(x)j_3(x) \frac{e^{i\sqrt{\mu } \frac{{\tilde{p}}}{|{\tilde{p}}|}\cdot ({\tilde{x}}-{\tilde{y}})}}{(2\pi )^2}B_{T_c^0}((0,{\tilde{p}} ),0)\nonumber \\  &   \qquad \times \left( V^{1/2} \Psi _{T_c^0(\lambda )}(y)-Vj_3(y) \right) {\textrm{d}}x {\textrm{d}}y{\textrm{d}}{\tilde{p}}\nonumber \\  &   \quad \mp \lambda \int _{{\tilde{p}}^2<2\mu }\int _{{\mathbb {R}}^6} V(x)j_3(x) \frac{e^{i\sqrt{\mu } \frac{{\tilde{p}}}{|{\tilde{p}}|}\cdot ({\tilde{x}}-{\tilde{y}})}}{(2\pi )^2}B_{T_c^0} ((0,{\tilde{p}} ),0) V(y)j_3(y){\textrm{d}}x {\textrm{d}}y{\textrm{d}}{\tilde{p}}.\nonumber \\ \end{aligned}$$

We prove that the first four integrals vanish as $\lambda \rightarrow 0$ and compute the limit of the expression in the last line.

Using (2.3), Lemma 3.7(iii) and $\Psi _{T_c^0(\lambda )}=V^{1/2}\Phi _{\lambda }$ the first term in (4.49) is bounded by

4.50 $$\begin{aligned} C \lambda \Vert V\Vert _1^{1/2} \Vert \Psi _{T_c^0(\lambda )}\Vert _2 \left\Vert \frac{1}{1+\vert \cdot \vert ^2}\widehat{V\Phi _{\lambda }}(0,\cdot )\right\Vert _{L^1({\mathbb {R}}^2)}, \end{aligned}$$

where *C* is independent of $\lambda$. By (4.36),

4.51 $$\begin{aligned} \left\Vert \frac{1}{1+\vert \cdot \vert ^2}\widehat{V\Phi _{\lambda }}(0,\cdot )\right\Vert _{L^1({\mathbb {R}}^2)}\le \left\| \frac{1}{1+\vert \cdot \vert ^2} \right\| _{L^{3/2}({\mathbb {R}}^{2})} \sup _{k_1} \Vert \widehat{V}(k_1,\cdot )\Vert _3 \Vert \widehat{\Phi }_{\lambda }\Vert _1. \nonumber \\ \end{aligned}$$

By Lemma 3.7(iv), $\sup _{k_1} \Vert \widehat{V}(k_1,\cdot )\Vert _3 <\infty$. Furthermore $\Vert \widehat{\Phi }_\lambda \Vert _1$ is bounded uniformly in $\lambda$ by Lemma 3.5. In total, the first term in (4.49) is $O(\lambda )$ as $\lambda \rightarrow 0$.

For the second line of (4.49) we use that

4.52 $$\begin{aligned} \sup _{\lambda >0} \sup _{{\tilde{x}},{\tilde{y}} \in {\mathbb {R}}^2} \left| \int _{{\mathbb {R}}^{2},{\tilde{p}}^2<2\mu }\frac{e^{i{\tilde{p}}\cdot ({\tilde{x}}-{\tilde{y}})}-e^{i\sqrt{\mu } \frac{{\tilde{p}}}{|{\tilde{p}}|}\cdot ({\tilde{x}}-{\tilde{y}})}}{(2\pi )^3}B_{T_c^0(\lambda )}((0,{\tilde{p}} ),0){\textrm{d}}{\tilde{p}} \right| <\infty ,\nonumber \\ \end{aligned}$$

as was shown in the proof of [9, Lemma 3.4]. Applying the Schwarz inequality, the second line is bounded by $C \lambda \Vert V \Vert _1\Vert \Psi _{T_c^0(\lambda )} \Vert _2^2$ for some constant *C* and vanishes for $\lambda \rightarrow 0$.

We bound the third line of (4.49) by

4.53 $$\begin{aligned}  &   \frac{\lambda }{(2\pi )^2} \int _{{\mathbb {R}}^{2},{\tilde{p}}^2<2\mu }\int _{{\mathbb {R}}^6} \vert \overline{\left( V^{1/2} \Psi _\lambda (x)-Vj_3(x) \right) }\vert \nonumber \\  &   \qquad \times B_{T_c^0(\lambda )}((0,{\tilde{p}} ),0) \vert V^{1/2} \Psi _{T_c^0(\lambda )} (y) \vert {\textrm{d}}x {\textrm{d}}y{\textrm{d}}{\tilde{p}}\nonumber \\  &   \quad \le \lambda \frac{\vert {\mathbb {S}}^1 \vert }{(2\pi )^2} m_\mu ^{d=2}(T_c^0(\lambda ))\Vert V \Vert _1 \Vert \Psi _{T_c^0(\lambda )} \Vert _2\Vert \Psi _{T_c^0(\lambda )}-\Psi \Vert _2, \end{aligned}$$

where in the second step we carried out the ${\tilde{p}}$ integration and used the Schwarz inequality in *x* and *y*. By Lemma 3.2, $\lambda m_\mu ^{d=2}(T_c^0(\lambda ))$ is bounded and by Lemma 3.3, $\Vert \Psi _{T_c^0(\lambda )} -\Psi \Vert _2$ decays like $\lambda ^{1/2}$. Hence, this vanishes for $\lambda \rightarrow 0$. Similarly, the fourth integral in (4.49) is bounded by

4.54 $$\begin{aligned} \lambda \frac{\vert {\mathbb {S}}^1 \vert }{(2\pi )^2} m_\mu ^{d=2}(T_c^0(\lambda ))\Vert V \Vert _1 \Vert V^{1/2}j_3 \Vert _2\Vert \Psi _{T_c^0(\lambda )} -\Psi \Vert _2, \end{aligned}$$

which vanishes for $\lambda \rightarrow 0$.

For the last line of (4.49) we first carry out the integration over *x*, *y* and the radial part of ${\tilde{p}}$, and then use that $\widehat{Vj_3}$ is a radial function. This way, we obtain that

4.55 $$\begin{aligned}  &   \mp \lambda m_\mu ^{d=2}(T_c^0(\lambda )) 2\pi \int _{{\mathbb {S}}^{1}}\vert \widehat{Vj_3}(0,\sqrt{\mu }w)\vert ^2{\textrm{d}}w\nonumber \\  &   \quad =\mp \lambda m_\mu ^{d=2}(T_c^0(\lambda )) \pi \int _{{\mathbb {S}}^{2}}\vert \widehat{Vj_3}(\sqrt{\mu }w)\vert ^2{\textrm{d}}w. \end{aligned}$$

The latter integral equals $\langle \vert V\vert ^{1/2}j_3,O_\mu V^{1/2} j_3\rangle = e_\mu \int _{{\mathbb {R}}^3} V(x)j_3(x)^2 {\textrm{d}}x$. By Lemma 3.2,

4.56 $$\begin{aligned} \lim _{\lambda \rightarrow 0}\lambda m_\mu ^{d=2}(T_c^0(\lambda ))e_\mu =\lim _{\lambda \rightarrow 0} \lambda \ln (\mu /T_c^0(\lambda ))e_\mu =\frac{1}{\mu ^{1/2}}. \end{aligned}$$

Therefore, the limit of the last line of (4.49) for $\lambda \rightarrow 0$ equals

4.57 $$\begin{aligned} \mp \frac{\pi }{\mu ^{1/2}} \int _{{\mathbb {R}}^3} V(x)j_3(x)^2 {\textrm{d}}x. \end{aligned}$$

*First term:* It remains to consider the first term in (4.45). If $V\ge 0$, one could argue directly using the convergence of $\Phi _\lambda$ in Lemma 3.6 for $d=3$. However, the analogue of Lemma 3.6 does not hold for $d=1$. Instead, the strategy is to use the $L^2$-convergence of the ground state in the Birman–Schwinger picture, Lemma 3.3. This approach also allows us to treat *V* that take negative values.

Switching to momentum space and using the eigenvalue equation (3.22), we rewrite the first term in (4.45) as

4.58 $$\begin{aligned}  &   (2\pi )^{1-\frac{d}{2}}\int _{{\mathbb {R}}^{2d-1}} \overline{\widehat{\Phi }_\lambda (p)} {\widehat{V}}(0,{\tilde{p}}-{\tilde{q}})\widehat{\Phi }_\lambda (p_1,{\tilde{q}}) {\textrm{d}}p {\textrm{d}}{\tilde{q}} \nonumber \\  &   \quad =(2\pi )^{1-\frac{d}{2}} \lambda ^2\langle \Psi _{T_c^0(\lambda )}, D_{T_c^0(\lambda )} \Psi _{T_c^0(\lambda )} \rangle , \end{aligned}$$

where $D_T$ is the operator given by

4.59 $$\begin{aligned} \langle \psi , D_T \psi \rangle= &   \int _{{\mathbb {R}}^{2d-1}} \overline{\widehat{|V|^{1/2} \psi } (p)} B_{T} (p,0) {\widehat{V}}(0,{\tilde{p}}-{\tilde{q}})\nonumber \\  &   \times B_{T}((p_1,{\tilde{q}}),0)\widehat{|V|^{1/2} \psi }(p_1,{\tilde{q}}) {\textrm{d}}p {\textrm{d}}{\tilde{q}} \end{aligned}$$

for $\psi \in L^2({\mathbb {R}}^d)$. We decompose (4.58) as

4.60 $$\begin{aligned}  &   (2\pi )^{1-\frac{d}{2}} \lambda ^2\langle \Psi _{T_c^0(\lambda )}, D_{T_c^0(\lambda )} \Psi _{T_c^0(\lambda )} \rangle \nonumber \\  &   \quad = (2\pi )^{1-\frac{d}{2}} \lambda ^2 \Big (\langle \Psi _{T_c^0(\lambda )}-\Psi , D_{T_c^0(\lambda )} \Psi _{T_c^0(\lambda )} \rangle +\langle \Psi , D_{T_c^0(\lambda )} ( \Psi _{T_c^0(\lambda )}- \Psi ) \rangle \nonumber \\  &   \qquad +\langle \Psi , D_{T_c^0(\lambda )} \Psi \rangle \Big ). \end{aligned}$$

Recall that by Lemma 3.3, $\Vert \Psi _{T_c^0} - \Psi \Vert _2=O(\lambda ^{1/2})$. The strategy is to prove that $\Vert D_T \Vert$ and $\langle \Psi , D_{T} \Psi \rangle$ are of the same order for $T\rightarrow 0$. Then, the positive term $\langle \Psi , D_{T_c^0(\lambda )} \Psi \rangle$ will be the leading order term in (4.60) as $\lambda \rightarrow 0$. The asymptotic behavior of $\Vert D_T \Vert$ and $\langle \Psi , D_{T} \Psi \rangle$ is the content of the following two Lemmas. These asymptotics strongly depend on the dimension and this is where the different treatment of $d=3$ versus $d\in \{1,2\}$ in Theorem 1.3 originates.

It will be convenient to introduce the operator $D_T^{<}$ as

4.61 $$\begin{aligned} \langle \psi , D_T^{<} \psi \rangle= &   \int _{\vert p|^2<2\mu , |(p_1,{\tilde{q}})|^2<2\mu , p_1^2<\mu } \overline{\widehat{|V|^{1/2} \psi } (p)} B_{T} (p,0) {\widehat{V}}(0,{\tilde{p}}-{\tilde{q}})\nonumber \\  &   \times B_{T}((p_1,{\tilde{q}}),0)\widehat{|V|^{1/2} \psi }(p_1,{\tilde{q}}) {\textrm{d}}p {\textrm{d}}{\tilde{q}} \end{aligned}$$

for $\psi \in L^2({\mathbb {R}}^d)$. Furthermore, for $d=2$ we define for $0<\delta <\mu$ the operator $D_T^\delta$ as

4.62 $$\begin{aligned} \langle \psi , D_T^{\delta } \psi \rangle= &   \int _{ \mu -\delta<p_1^2<\mu ,p_2^2<2\delta , q_2^2<2\delta } \overline{\widehat{|V|^{1/2} \psi } (p)} B_{T} (p,0) {\widehat{V}}(0,p_2- q_2)\nonumber \\  &   \times B_{T}((p_1,{\tilde{q}}),0)\widehat{|V|^{1/2} \psi }(p_1, q_2) {\textrm{d}}p {\textrm{d}}q_2 \end{aligned}$$

for $$\psi \in L^2({\mathbb {R}}^2)$$.

#### Lemma 4.10

Let $\mu>\delta >0$ and let *V* satisfy 1.1. There are constants $C,T_0>0$ such that for all $0<T<T_0$ for $d=1$ $\Vert D_T \Vert \le C/T$, for $d=2$ $\Vert D_T \Vert \le C(\ln \mu /T)^3$ and $\Vert D_T-D_T^\delta \Vert \le C (\ln \mu /T)^2$, and for $d=3$ $\Vert D_T \Vert \le C(\ln \mu /T)^2$ and $\Vert D_T-D_T^< \Vert \le C \ln \mu /T$.

#### Lemma 4.11

Let $\mu >0$ and let *V* satisfy 1.1. Recall that $\Psi =V^{1/2}j_d$. There are constants $C,T_0>0$ such that for all $0<T<T_0$, $\langle \Psi , D_T \Psi \rangle \ge C/T$ for $d=1$ and $\ge C(\ln \mu /T)^3$ for $d=2$. For $d=3$, $\lim _{\lambda \rightarrow 0} (2\pi )^{-1/2} \lambda ^2\langle \Psi , D_{T_c^0(\lambda )} \Psi \rangle = \int _{{\mathbb {R}}^4} V(r) j_3(z_1,{\tilde{r}};\mu )^2 {\textrm{d}}r {\textrm{d}}z_1$.

For $\lambda \rightarrow 0$, by Lemma 3.2, $\ln (\mu /T_c^0(\lambda ))$ is of order $1/\lambda$, hence the last term in (4.60) diverges for $d=1,2$. For $d=3$ we get the desired constant by Lemma 4.11.$\square$

#### Proof of Lemma 4.10

Assume that $T/\mu <1/2$. We treat the different dimensions *d* separately.

*Dimension one:* Note that

4.63 $$\begin{aligned} |\langle \psi , D_T \psi \rangle |= &   |\widehat{V}(0)| \int _{\mathbb {R}}B_{T} (p,0)^2 \vert \widehat{|V|^{1/2} \psi }(p) \vert ^2 {\textrm{d}}p \nonumber \\\le &   \Vert V \Vert _1^2 \int _{\mathbb {R}}B_{T} (p,0)^2 {\textrm{d}}p \Vert \psi \Vert _2^2, \end{aligned}$$

where we used Lemma 3.7. Recall from (2.3) that $B_{T} (p,0) \le \min \left\{ \frac{1}{\vert p^2-\mu \vert }, \frac{1}{2T}\right\}$. We estimate the integral

4.64 $$\begin{aligned}  &   \int _{\mathbb {R}}B_{T} (p,0)^2 {\textrm{d}}p\nonumber \\  &   \quad \le \int _{\sqrt{\mu }-\frac{T}{\sqrt{\mu }}< \vert p \vert<\sqrt{\mu }+\frac{T}{\sqrt{\mu }} }\frac{1}{4T^2} {\textrm{d}}p+\int _{{\mathbb {R}}} \frac{\chi _{\vert p \vert< \sqrt{\mu }-\frac{T}{\sqrt{\mu }}}+\chi _{ \sqrt{\mu }+\frac{T}{\sqrt{\mu }}<p<2\sqrt{\mu }}}{\mu (\vert p \vert -\sqrt{\mu } )^2} {\textrm{d}}p \nonumber \\  &   \qquad + \int _{ p>2\sqrt{\mu }} \frac{1}{( p^2-\mu )^2} {\textrm{d}}p. \end{aligned}$$

The first term equals $(\sqrt{\mu }T)^{-1}$. The last term is a finite constant independent of *T*. In the second term we substitute $\vert \vert p \vert -\sqrt{\mu }\vert$ by *x* and get the bound

4.65 $$\begin{aligned} 2 \int _{\frac{T}{\sqrt{\mu }}}^{\sqrt{\mu }} \frac{1}{\mu x^2 } {\textrm{d}}x = \frac{2}{\sqrt{\mu }}(1/T-1/\mu ). \end{aligned}$$

*Dimension two:* Using the Schwarz inequality we have

4.66 $$\begin{aligned} \langle \psi , D_T \psi \rangle \le C \Vert V\Vert _1^2 \int _{{\mathbb {R}}^{3}} B_{T,\mu } (p,0) B_{T,\mu }((p_1,{\tilde{q}}),0){\textrm{d}}p {\textrm{d}}{\tilde{q}} \Vert \psi \Vert _2^2. \end{aligned}$$

The integral can be rewritten as

4.67 $$\begin{aligned} \int _{\mathbb {R}}\left( \int _{\mathbb {R}}B_{T, \mu -p_1^2}(p_2, 0) {\textrm{d}}p_2 \right) ^2 {\textrm{d}}p_1, \end{aligned}$$

where $B_{T,\mu }$ here is understood as the function on ${\mathbb {R}}\times {\mathbb {R}}$ instead of ${\mathbb {R}}^2\times {\mathbb {R}}^2$. Similarly,

4.68 $$\begin{aligned} \vert \langle \psi , (D_T-D_T^\delta )\psi \rangle \vert\le &   C \Vert V\Vert _1^2 \int _{{\mathbb {R}}^{3}}(1-\chi _{\mu -\delta<p_1^2<\mu } \chi _{p_2^2<2\delta }\chi _{p_2'^2<2\delta })\nonumber \\  &   \times B_{T,\mu } (p,0) B_{T,\mu }((p_1,{\tilde{q}}),0){\textrm{d}}p {\textrm{d}}{\tilde{q}} \Vert \psi \Vert _2^2. \end{aligned}$$

We prove that (4.67) and (4.68) are of order $O(\ln (\mu /T)^3)$ and $O(\ln (\mu /T)^2)$ for $T\rightarrow 0$, respectively. To bound the integrals we consider three regimes, $p_1^2<\mu -T$, $\mu -T<p_1^2<\mu +T$, and $\mu +T<p_1^2$. Corresponding to these regimes, we need to understand $\int _{\mathbb {R}}B_{T, \mu }(p, 0) {\textrm{d}}p$ for $T/\mu <1$, $-1<\mu /T <1$, and $\mu /T<-1$.

In the first regime, there is a constant $C_1$, such that, for all $T/\mu <1$,

4.69 $$\begin{aligned} \left| \sqrt{\mu } \int _{{\mathbb {R}}} B_{T,\mu }(p,0)\chi _{p^2<2\mu } {\textrm{d}}p - 2 \ln \frac{\mu }{T} \right| +\left| \sqrt{\mu } \int _{{\mathbb {R}}} B_{T,\mu }(p,0)\chi _{p^2>2\mu } {\textrm{d}}p \right| \le C_1.\nonumber \\ \end{aligned}$$

This follows from rescaling $\sqrt{\mu } \int _{\mathbb {R}}B_{T,\mu }(p,0) {\textrm{d}}p = \int _{\mathbb {R}}B_{T/\mu ,1} (p,0) {\textrm{d}}p$ and applying [6, Lemma 3.5]. For the second regime, we rewrite

4.70 $$\begin{aligned} \int _{\mathbb {R}}B_{T,\mu }(p,0) {\textrm{d}}p = \frac{1}{\sqrt{T}}\int _{\mathbb {R}}\frac{\tanh ((p^2-\mu /T)/2)}{p^2-\mu /T} {\textrm{d}}p. \end{aligned}$$

Since $\tanh (x)/x \le \min \{1,1/\vert x\vert \}$, the latter integral is uniformly bounded for $\vert \mu /T \vert <1$:

4.71 $$\begin{aligned} \int _{\mathbb {R}}B_{T,\mu }(p,0) {\textrm{d}}p \le \frac{C_2}{\sqrt{T}}. \end{aligned}$$

For the third regime, it follows from (4.70) that

4.72 $$\begin{aligned} \int _{\mathbb {R}}B_{T,\mu }(p,0) {\textrm{d}}p\le \frac{1}{\sqrt{T}}\int _{\mathbb {R}}\frac{1}{p^2-\mu /T} {\textrm{d}}p= \frac{1}{\sqrt{-\mu }} \int _{\mathbb {R}}\frac{1}{p^2+1} {\textrm{d}}p=:\frac{C_3}{\sqrt{-\mu }}. \nonumber \\ \end{aligned}$$

Combining the bounds in the three regimes, we bound (4.67) from above by

4.73 $$\begin{aligned}  &   \int _{\vert p_1 \vert<\sqrt{\mu -T}} \frac{\left( 2 \ln \left( \frac{\mu -p_1^2}{T}\right) +C_1\right) ^2}{\mu -p_1^2} {\textrm{d}}p_1\nonumber \\  &   \quad +\int _{\sqrt{\mu -T}<\vert p_1 \vert<\sqrt{\mu +T}} \frac{C_2^2}{T} {\textrm{d}}p_1+\int _{\sqrt{\mu +T}<\vert p_1 \vert } \frac{C_3^2}{p_1^2-\mu } {\textrm{d}}p_1. \end{aligned}$$

The first integral is bounded above by

4.74 $$\begin{aligned} \left( 2 \ln \left( \frac{\mu }{T}\right) +C_1\right) ^2 \int _{\vert p_1 \vert <\sqrt{\mu -T}} \frac{1}{\mu -p_1^2} {\textrm{d}}p_1. \end{aligned}$$

Since

4.75 $$\begin{aligned} \int _{\vert p_1 \vert <\sqrt{\mu -T}} \frac{1}{\mu -p_1^2} {\textrm{d}}p_1=\frac{1}{\sqrt{\mu }} \ln \left( \frac{2\mu -T+\sqrt{\mu (\mu -T)}}{T}\right) =O(\ln (\mu /T)),\nonumber \\ \end{aligned}$$

the first integral in (4.73) is of order $O(\ln (\mu /T)^3)$. In the second integral, the size of the integration domain is $2T/\sqrt{\mu }+O(T^2)$, so the integral is bounded as $T\rightarrow 0$. The third integral equals

4.76 $$\begin{aligned} \frac{C_3^2}{\sqrt{\mu }} \ln \left( \frac{2\mu +T+\sqrt{\mu (\mu +T)}}{T}\right) =O(\ln \mu /T). \end{aligned}$$

In total (4.67) is of order $O(\ln (\mu /T)^3)$.

For the integral in (4.68) we obtain the upper bound similar to (4.73). The main difference is that in the regime $\sqrt{\mu -\delta }<\vert p_1 \vert <\sqrt{\mu -T}$, at least one of the variables $p_2,p_2'$ is constrained to absolute values larger than $\sqrt{2\delta }\ge \sqrt{2(\mu -p_1^2})$, and thus for the integration over this variable there will be no $\ln \left( \frac{\mu -p_1^2}{T}\right)$ contribution from (4.69). The upper bound for (4.68) is

4.77 $$\begin{aligned}  &   \int _{\vert p_1 \vert<\sqrt{\mu -\delta }} \frac{\left( 2 \ln \left( \frac{\mu -p_1^2}{T}\right) +C_1\right) ^2}{\mu -p_1^2} {\textrm{d}}p_1 \nonumber \\  &   \quad +\int _{\sqrt{\mu -\delta }<\vert p_1 \vert<\sqrt{\mu -T}} \frac{2\left( 2 \ln \left( \frac{\mu -p_1^2}{T}\right) +C_1\right) C_1}{\mu -p_1^2} {\textrm{d}}p_1\nonumber \\  &   \quad +\int _{\sqrt{\mu -T}<\vert p_1 \vert<\sqrt{\mu +T}} \frac{C_2^2}{T} {\textrm{d}}p_1 +\int _{\sqrt{\mu +T}<\vert p_1 \vert } \frac{C_3^2}{p_1^2-\mu } {\textrm{d}}p_1. \end{aligned}$$

We have already seen above that the last two integrals are of order *O*(1) and $O(\ln \mu /T)$, respectively. The first integral in (4.77) is bounded above by $\left( 2 \ln \left( \frac{\mu }{T}\right) +\right. \left. C_1\right) ^2 \int _{\vert p_1 \vert <\sqrt{\mu -\delta }} \frac{1}{\mu -p_1^2} {\textrm{d}}p_1=O(\ln (\mu /T)^2).$ Similarly, the second integral in (4.77) is of order $O(\ln (\mu /T)^2)$ by (4.75).

*Dimension three:* For $d=3$, we first prove that $\Vert D_T^<\Vert =O(\ln (\mu /T)^2)$. We bound (4.61) using the Schwarz inequality

4.78 $$\begin{aligned} \langle \psi , D_T^< \psi \rangle\le &   \Vert V \Vert ^2_1 \Vert \psi \Vert _2^2 \int _{{\mathbb {R}}^{5}}\chi _{|p|^2<2\mu , |(p_1,{\tilde{q}})|<2\mu , p_1^2<\mu } B_{T,\mu } (p,0)\nonumber \\  &   \times B_{T,\mu }((p_1,{\tilde{q}}),0){\textrm{d}}p {\textrm{d}}{\tilde{q}}. \end{aligned}$$

The integral can be rewritten as

4.79 $$\begin{aligned} 4\pi ^2 \int _0^{\sqrt{\mu }} \left( \int _{0}^{\sqrt{2\mu -p_1^2}} B_{T, \mu -p_1^2}(t, 0) t {\textrm{d}}t \right) ^2 {\textrm{d}}p_1. \end{aligned}$$

Substituting $$s=(t^2+p_1^2-\mu )/T$$ gives

4.80 $$\begin{aligned} \begin{aligned} \pi ^2 \int _0^{\sqrt{\mu }} \left( \int _{-(\mu -p_1^2)/T}^{\mu /T} \frac{\tanh (s)}{s} {\text {d}}s \right) ^2 {\text {d}}p_1 \le \sqrt{\mu }\pi ^2 \left( \int _{-\mu /T}^{\mu /T} \frac{\tanh (s)}{s} {\text {d}}s \right) ^2, \end{aligned}\nonumber \\ \end{aligned}$$

since $\tanh (x)/x \le \min \{1,1/\vert x\vert \}$, this is bounded by

4.81 $$\begin{aligned} \sqrt{\mu }4\pi ^2 \left( 1+\ln (\mu /T) \right) ^2. \end{aligned}$$

To bound $\Vert D_T-D_T^<\Vert$, we distinguish the cases were $p^2$ and $(p_1,{\tilde{q}})^2$ are larger or smaller than $2\mu$. Using the bound on $B_T$ given in (2.3), we estimate that

4.82 $$\begin{aligned}  &   \vert \langle \psi , (D_T-D_T^< )\psi \rangle \vert \nonumber \\  &   \quad \le \Vert V \Vert ^2_1 \Vert \psi \Vert _2^2 \int _{{\mathbb {R}}^{5}}\chi _{|p|^2<2\mu , |(p_1,{\tilde{q}})|<2\mu ,p_1^2>\mu } B_{T,\mu } (p,0) B_{T,\mu }((p_1,{\tilde{q}}),0){\textrm{d}}p {\textrm{d}}{\tilde{q}}\nonumber \\  &   \qquad +2\Vert V \Vert _1 \Vert \psi \Vert _2^2 \int _{{\mathbb {R}}^{5}} \frac{C}{{\tilde{p}}^2+1}\vert {\widehat{V}}(0,{\tilde{p}}-{\tilde{q}})\vert B_{T,\mu }((p_1,{\tilde{q}}),0)\chi _{|(p_1,{\tilde{q}})|^2<2\mu }{\textrm{d}}p {\textrm{d}}{\tilde{q}}\nonumber \\  &   \qquad +\int _{{\mathbb {R}}^{5}} \overline{\widehat{|V|^{1/2} \psi } (p)} \frac{C}{p^2+1}\vert {\widehat{V}}(0,{\tilde{p}}-{\tilde{q}}) \vert \frac{C }{p_1^2+{\tilde{q}}^2+1}\widehat{|V|^{1/2} \psi }(p_1,{\tilde{q}}) {\textrm{d}}p {\textrm{d}}{\tilde{q}},\nonumber \\ \end{aligned}$$

where *C* is a constant independent of *T*. For the first term, proceeding similarly to (4.79)–(4.81), the integral equals

4.83 $$\begin{aligned} \pi ^2 \int _{\sqrt{\mu }}^{\sqrt{2\mu }} \left( \int _{(p_1^2-\mu )/T}^{\mu /T} \frac{\tanh (s)}{s} {\textrm{d}}s \right) ^2 {\textrm{d}}p_1 \le \pi ^2 \int _{\sqrt{\mu }}^{\sqrt{2\mu }} \ln \left( \frac{\mu }{p_1^2-\mu }\right) ^2 {\textrm{d}}p_1<\infty \nonumber \\ \end{aligned}$$

For the second term in (4.82) we apply Young’s inequality to bound the integral by

4.84 $$\begin{aligned} C \left\| \frac{1}{1+|\cdot |^2}\right\| _{L^{3/2}({\mathbb {R}}^2)} \Vert {\widehat{V}}(0,\cdot ) \Vert _{L^3({\mathbb {R}}^2)} |{\mathbb {S}}^2| m_\mu (T), \end{aligned}$$

which is $O(\ln \mu /T)$. The third term in (4.82) is bounded by $C \Vert \psi \Vert _2^2$ by Lemma 4.6. $\square$

#### Proof of Lemma 4.11

By assumption, $0<e_\mu = \frac{1}{(2\pi )^{d/2}}\int _{{\mathbb {S}}^{d-1}} {\widehat{V}}(p- \sqrt{\mu } \omega ) {\textrm{d}}\Omega (\omega )=\widehat{V j_d}(\vert p\vert =\sqrt{\mu })$. By continuity of $\widehat{Vj_d}(p)$ in *p*, there is an $\epsilon >0$ such that $\widehat{V j_d}(p)>\frac{1}{2}\widehat{V j_d}(\vert p\vert =\sqrt{\mu })>0$ for all $\sqrt{\mu }-\epsilon< \vert p\vert < \sqrt{\mu }+\epsilon$. In that follows we treat the different dimensions separately.

*Dimension one:* Suppose $T<\epsilon$. Since $\widehat{V}(0) >0$,

4.85 $$\begin{aligned} \begin{aligned}&\langle V^{1/2} j_1, D_T V^{1/2} j_1\rangle = \widehat{V}(0) \int _{\mathbb {R}}B_{T} (p,0)^2 \vert \widehat{V j_1}(p) \vert ^2 {\text {d}}p \\  &\quad \ge \frac{1}{4}\widehat{V}(0) \vert \widehat{V j_1}(\sqrt{\mu }) \vert ^2 \int _{\sqrt{\mu }+T}^{\sqrt{\mu }+\epsilon } B_{T} (p,0)^2 {\text {d}}p \end{aligned}\end{aligned}$$

For $p\in [\sqrt{\mu }+T,\sqrt{\mu }+\epsilon ]$, $B_{T} (p,0)\ge \frac{\tanh (\sqrt{\mu })}{p^2-\mu } \ge \frac{\tanh (\sqrt{\mu })}{(2\sqrt{\mu }+\epsilon )(p-\sqrt{\mu })}$. Since $\int _{\sqrt{\mu }+T}^{\sqrt{\mu }+\epsilon } \frac{1}{(p-\sqrt{\mu })^2}{\textrm{d}}p = 1/T-1/\epsilon$, we obtain the lower bound

4.86 $$\begin{aligned} \langle V^{1/2} j_1, D_T V^{1/2} j_1\rangle \ge \frac{1}{4}\widehat{V}(0) \vert \widehat{V j_1}(\sqrt{\mu }) \vert ^2 \frac{\tanh (\sqrt{\mu })^2}{(2\sqrt{\mu }+\epsilon )^2} \left( \frac{1}{T}-\frac{1}{\epsilon }\right) , \end{aligned}$$

and the claim follows.

**Dimension two:** Since $\widehat{V}(0)> 0$, by continuity also $\widehat{V}(p)>0$ for small |*p*|. Therefore, there are constants $0<\delta <\mu$ and $C>0$ such that for all $\sqrt{\mu -\delta }<p_1\le \sqrt{\mu }$ and $|p_2|,|q_2|<(2\delta )^{1/2}$

4.87 $$\begin{aligned} \overline{\widehat{V j_2} (p_1,p_2)} {\widehat{V}}(0,p_2-q_2) \widehat{V j_2}(p_1,q_2) >C. \end{aligned}$$

By Lemma 4.10, we have

$$\langle V^{1/2} j_2, D_T V^{1/2} j_2 \rangle = \langle V^{1/2} j_2, D_T^\delta V^{1/2} j_2 \rangle +O((\ln \mu /T)^2).$$

It hence suffices to show that $\langle V^{1/2} j_2, D_T^\delta V^{1/2} j_2 \rangle$ grows like $(\ln \mu /T)^3$. Let $A:=\{(p_1,p_2,q_2)\in {\mathbb {R}}^3 | \sqrt{\mu -\delta }<p_1<\sqrt{\mu }, 0<p_2,q_2<\delta ^{1/2}, p_1^2+p_2^2>\mu +T,p_1^2+q_2^2>\mu +T\}$. This is a subset of the support in $D_T^\delta$. Using that all terms in the integrand of $\langle V^{1/2} j_2, D_T^\delta V^{1/2} j_2\rangle$ are positive, we estimate

4.88 $$\begin{aligned} \langle V^{1/2} j_2, D_T^\delta V^{1/2} j_2 \rangle \ge C \int _{A} B_{T} (p,0) B_{T}((p_1, q_2),0) {\textrm{d}}p {\textrm{d}}q_2. \end{aligned}$$

For $(p_1,p_2,q_2)\in A$ we have $p_1^2+p_2^2-\mu >T$, and thus,

4.89 $$\begin{aligned} B_{T} (p,0)\ge \frac{\tanh \left( \frac{1}{2}\right) }{ p_1^2+p_2^2-\mu }. \end{aligned}$$

For $$p_1^2>\mu +T-\delta $$

4.90 $$\begin{aligned} \begin{aligned}&\int _{\sqrt{\mu +T-p_1^2}}^{\delta ^{1/2}} \frac{1}{p_1^2+p_2^2-\mu }{\text {d}}p_2 =\frac{1}{\sqrt{\mu -p_1^2}} \\  &\quad \times \left[ {{\,\text {artanh}\,}}\left( \sqrt{1-\frac{T}{\mu +T-p_1^2}}\right) -{{\,\text {artanh}\,}}\left( \sqrt{\frac{\mu -p_1^2}{\delta }}\right) \right] . \end{aligned} \end{aligned}$$

Hence, the integral in (4.88) is bounded below by

4.91 $$\begin{aligned} \begin{aligned} \tanh \left( \frac{1}{2}\right) ^2&\int _{\sqrt{\mu +T-\delta }}^{\sqrt{\mu }} \frac{1}{\mu -p_1^2} \left[ {{\,\text {artanh}\,}}\left( \sqrt{1-\frac{T}{\mu +T-p_1^2}}\right) \right. \\  &\quad \left. -{{\,\text {artanh}\,}}\left( \sqrt{\frac{\mu -p_1^2}{\delta }}\right) \right] ^2 {\text {d}}p_1 \end{aligned} \end{aligned}$$

Assume that $T<\delta /2$. For a lower bound, we further restrict the $p_1$-integration to the interval $\left( \sqrt{\mu -\delta /2}, \sqrt{\mu -\mu ^{1/2}T^{1/2}}\right) .$ For these values of $p_1$, we have

4.92 $$\begin{aligned} {{\,\textrm{artanh}\,}}\left( \sqrt{\frac{\mu -p_1^2}{\delta }}\right)\le &   {{\,\textrm{artanh}\,}}\left( \frac{1}{\sqrt{2}}\right) \le {{\,\textrm{artanh}\,}}\left( \sqrt{1-\frac{T^{1/2}}{\mu ^{1/2}}}\right) \nonumber \\\le &   {{\,\textrm{artanh}\,}}\left( \sqrt{1-\frac{T}{\mu +T-p_1^2}}\right) . \end{aligned}$$

Furthermore,

4.93 $$\begin{aligned} \int _{\sqrt{\mu -\delta /2}}^{\sqrt{\mu -\mu ^{1/2}T^{1/2}}} \frac{1}{\mu -p_1^2} {\textrm{d}}p_1 = \frac{1}{\sqrt{\mu }}{{\,\textrm{artanh}\,}}\left( 1-\frac{(\sqrt{\mu }/a+1)(1-b/\sqrt{\mu })}{\sqrt{\mu }/a-b/\sqrt{\mu }}\right) ,\nonumber \\ \end{aligned}$$

where $a=\sqrt{\mu -\delta /2}$ and $b=\sqrt{\mu -\mu ^{1/2}T^{1/2}} \le \sqrt{\mu }$. This is bounded below by

4.94 $$\begin{aligned} \frac{1}{\sqrt{\mu }}{{\,\textrm{artanh}\,}}\left( 1-\frac{(\sqrt{\mu }/a+1)(1-b/\sqrt{\mu })}{\sqrt{\mu }/a-1}\right) . \end{aligned}$$

In total, (4.91) is bounded from below by

4.95 $$\begin{aligned}  &   \frac{1}{\sqrt{\mu }}\tanh \left( \frac{1}{2}\right) ^2 \left( {{\,\textrm{artanh}\,}}\left( \sqrt{1-\frac{T^{1/2}}{\mu ^{1/2}}}\right) -{{\,\textrm{artanh}\,}}\left( \frac{1}{\sqrt{2}}\right) \right) ^2\nonumber \\  &   \quad \times {{\,\textrm{artanh}\,}}\left( 1-\frac{(\sqrt{\mu }/a+1)(1-\sqrt{1-(T/\mu )^{1/2}})}{\sqrt{\mu }/a-1}\right) . \end{aligned}$$

With ${{\,\textrm{artanh}\,}}(1-x) = \frac{1}{2} \ln 2/x+o(1)$ as $x\rightarrow 0$, we obtain that, for $T\rightarrow 0$,

4.96 $$\begin{aligned} {{\,\textrm{artanh}\,}}\left( \sqrt{1-\frac{T^{1/2}}{\mu ^{1/2}}}\right) =\frac{1}{4}\ln \left( 16\frac{\mu }{T}\right) +o(1) \end{aligned}$$

and

4.97 $$\begin{aligned}  &   {{\,\textrm{artanh}\,}}\left( 1-\frac{(\sqrt{\mu }/a+1)(1-\sqrt{1-(T/\mu )^{1/2}})}{\sqrt{\mu }/a-1}\right) \nonumber \\  &   \quad =\frac{1}{4}\ln \left( 16\left( \frac{\sqrt{\mu }/a-1}{\sqrt{\mu }/a+1}\right) ^2\frac{\mu }{T}\right) +o(1). \end{aligned}$$

In particular, we obtain

4.98 $$\begin{aligned} \langle V^{1/2} j_2, D_T V^{1/2} j_2 \rangle \ge \frac{C }{\sqrt{\mu }} \ln \left( \frac{\mu }{T}\right) ^3+O\left( \ln \left( \frac{\mu }{T}\right) ^2\right) \end{aligned}$$

for some $C>0$, which implies the claim.

*Dimension three:* Using that $\Vert D_T-D_T^<\Vert \le C \ln \mu /T$ according to Lemma 4.10 and that $\ln \mu /T_c^0(\lambda )\sim 1/\lambda$ by Lemma 3.2,

4.99 $$\begin{aligned} \lim _{\lambda \rightarrow 0} \lambda ^2\langle V^{1/2}j_3, D_{T_c^0(\lambda )} V^{1/2}j_3 \rangle = \lim _{\lambda \rightarrow 0}\lambda ^2\langle V^{1/2}j_3, D_{T_c^0(\lambda )}^< V^{1/2}j_3 \rangle . \end{aligned}$$

By integrating out the angular variables

$$\begin{aligned} \int _{{\mathbb {R}}^{3}} V(r) j_3(r;\mu ) \frac{e^{i \sqrt{\mu }r \cdot p/\vert p \vert }}{(2\pi )^{3/2}} {\text {d}}r = \frac{1}{|{\mathbb {S}}^2|}\int _{{\mathbb {R}}^3} V(r) j_3(r;\mu )^2{\text {d}}r=e_\mu . \end{aligned}$$

Therefore, we can write

4.100 $$\begin{aligned} \begin{aligned}&\langle V^{1/2}j_3, D_{T_c^0(\lambda )}^< V^{1/2}j_3 \rangle \\  &\quad =\frac{1}{(2\pi )^{3}}\int _{{\mathbb {R}}^{11}; {\tilde{p}}^2, {\tilde{q}}^2<2\mu -p_1^2, p_1^2<\mu } \Big [ V j_3(r;\mu )(e^{i r \cdot p}-e^{i \sqrt{\mu }r \cdot p/\vert p \vert })B_{T_c^0(\lambda )} (p,0) \\  &\qquad \times {\widehat{V}}(0,{\tilde{p}}-{\tilde{q}}) B_{T_c^0(\lambda )}((p_1,{\tilde{q}}),0)e^{-i p \cdot r'} V j_3(r';\mu ) \\  &\qquad + V j_3(r;\mu )e^{i \sqrt{\mu }r \cdot p/\vert p \vert } B_{T_c^0(\lambda )} (p,0) {\widehat{V}}(0,{\tilde{p}}-{\tilde{q}}) B_{T_c^0(\lambda )}((p_1,{\tilde{q}}),0)\\  &\qquad \times (e^{-i p \cdot r'}-e^{-i \sqrt{\mu }r' \cdot p/\vert p \vert }) V j_3(r';\mu ) \Big ]{\text {d}}p {\text {d}}{\tilde{q}} {\text {d}}r {\text {d}}r' \\  &\qquad +e_\mu ^2 \int _{{\mathbb {R}}^{8}; {\tilde{p}}^2, {\tilde{q}}^2<2\mu -p_1^2, p_1^2<\mu }\! B_{T_c^0(\lambda )} (p,0) \frac{e^{i ({\tilde{p}}-{\tilde{q}}){\tilde{r}}}}{(2\pi )^{3/2}}V(r) \\  &\qquad \times B_{T_c^0(\lambda )}((p_1,{\tilde{q}}),0){\text {d}}p {\text {d}}{\tilde{q}} {\text {d}}r \\ \end{aligned}\end{aligned}$$

By [8, Proof of Lemma 3.1]

4.101 $$\begin{aligned} \left| \int _{{\mathbb {S}}^2} e^{i \vert r \vert w \cdot p}-e^{i \sqrt{\mu } |r| w \cdot p/\vert p \vert }{\textrm{d}}w \right| \le C \frac{|p|-\sqrt{\mu }}{|p|+\sqrt{\mu }}. \end{aligned}$$

Furthermore, note that $B_{T} (p,0) \frac{|p|-\sqrt{\mu }}{|p|+\sqrt{\mu }} \le \frac{1}{\mu }$. Hence, the first integral in (4.100) is bounded by

4.102 $$\begin{aligned}  &   \frac{C}{\mu }\Vert V j_3 \Vert _1^2 \Vert {\widehat{V}} \Vert _\infty \int _{p_1^2+{\tilde{q}}^2<2\mu , {\tilde{p}}^2<2\mu } B_{T_c^0(\lambda )}((p_1,{\tilde{q}}),0) {\textrm{d}}p_1 {\textrm{d}}{\tilde{p}} {\textrm{d}}{\tilde{q}}\nonumber \\  &   \quad \le C\Vert V j_3 \Vert _1^2 \Vert {\widehat{V}} \Vert _\infty m_\mu (T_c^0(\lambda )), \end{aligned}$$

which is of order $1/\lambda$ by Lemma 3.2.

Changing to angular coordinates for the ${\tilde{p}}$ and ${\tilde{q}}$ integration, the integral on the last line of (4.100) can be rewritten as

4.103 $$\begin{aligned} \begin{aligned}&2 \int _{{\mathbb {R}}^3} {\text {d}}r\int _0^{\sqrt{\mu }} {\text {d}}p_1 \int _0^{\sqrt{2\mu -p_1^2}} {\text {d}}t \int _0^{\sqrt{2\mu -p_1^2}} {\text {d}}s \int _{{\mathbb {S}}^1}\! {\text {d}}w \int _{{\mathbb {S}}^1}\! {\text {d}}w' \frac{e^{i (t w-s w')\cdot {\tilde{r}}}}{(2\pi )^{3/2}} \\  &\qquad \times B_{T_c^0(\lambda )}(\sqrt{p_1^2+t^2},0) t V(r)B_{T_c^0(\lambda )}(\sqrt{p_1^2+s^2},0) s \\  &\quad =2 \int _{{\mathbb {R}}^3} {\text {d}}r \int _0^{\sqrt{\mu }} {\text {d}}p_1 \int _{p_1}^{\sqrt{2\mu }} {\text {d}}x \int _{p_1}^{\sqrt{2\mu }} {\text {d}}y \int _{{\mathbb {S}}^1} {\text {d}}w \int _{{\mathbb {S}}^1} {\text {d}}w' B_{T_c^0(\lambda )} (x,0) x \\  &\qquad \times \frac{e^{i (\sqrt{x^2-p_1^2} w-\sqrt{y^2-p_1^2} w')\cdot {\tilde{r}}}}{(2\pi )^{3/2}} V(r) B_{T_c^0(\lambda )}(y,0) y. \end{aligned}\end{aligned}$$

where we substituted $x=\sqrt{p_1^2+t^2}, y=\sqrt{p_1^2+s^2}$. Next, we want to replace the $x^2$ and $y^2$ in the exponent by $\mu$. We rewrite (4.103) as

4.104 $$\begin{aligned}  &   2\int B_{T_c^0(\lambda )} (x,0) x \frac{\left( e^{i \sqrt{x^2-p_1^2} w\cdot {\tilde{r}}}-e^{i \sqrt{\mu -p_1^2} w\cdot {\tilde{r}}}\right) }{(2\pi )^{3/2}}V(r)e^{-i \sqrt{y^2-p_1^2} w'\cdot {\tilde{r}}}\nonumber \\  &   \quad \times B_{T_c^0(\lambda )}(y,0) y{\textrm{d}}p_1{\textrm{d}}r {\textrm{d}}x {\textrm{d}}y {\textrm{d}}w{\textrm{d}}w'+2 \int B_{T_c^0(\lambda )} (x,0) x e^{i \sqrt{\mu -p_1^2} w\cdot {\tilde{r}}}\nonumber \\  &   \quad \times V(r)\frac{\left( e^{-i \sqrt{y^2-p_1^2} w'\cdot {\tilde{r}}}-e^{i \sqrt{\mu -p_1^2} w'\cdot {\tilde{r}}}\right) }{(2\pi )^{3/2}} B_{T_c^0(\lambda )}(y,0) y{\textrm{d}}p_1{\textrm{d}}r {\textrm{d}}x {\textrm{d}}y {\textrm{d}}w{\textrm{d}}w'\nonumber \\  &   \quad +2 \int B_{T_c^0(\lambda )} (x,0) x \frac{e^{i \sqrt{\mu -p_1^2} (w-w')\cdot {\tilde{r}}}}{(2\pi )^{3/2}}V(r) B_{T_c^0(\lambda )}(y,0) y{\textrm{d}}p_1{\textrm{d}}r {\textrm{d}}x {\textrm{d}}y {\textrm{d}}w{\textrm{d}}w'.\nonumber \\ \end{aligned}$$

By [9, Proof of Lemma 3.4]

4.105 $$\begin{aligned} \left| \int _{{\mathbb {S}}^1} \frac{e^{i \sqrt{x-p_1^2} w \cdot {\tilde{r}}}-e^{i \sqrt{\mu -p_1^2} w \cdot {\tilde{r}}}}{(2\pi )^{2}} {\textrm{d}}w \right|\le &   C \left| \sqrt{x^2-p_1^2}-\sqrt{\mu -p_1^2}\right| ^{1/3}\nonumber \\  &   \times \left| (x^2-p_1^2)^{-1/6}+(\mu -p_1^2)^{-1/6}\right| .\nonumber \\ \end{aligned}$$

We bound this further by $C \left| x^2-\mu \right| ^{1/3}\left( (x^2-p_1^2)^{-1/3}+(\mu -p_1^2)^{-1/3}\right)$. Using that $B_{T_c^0(\lambda )}(x,0)\le 1/|x^2-\mu |$ by (2.3) and recalling the definition of $m_\mu$ in (3.2) we bound the first two lines in (4.104) by

4.106 $$\begin{aligned} \begin{aligned}&C \Vert V \Vert _1 m_{\mu }^{d=2}(T_c^0(\lambda )) \int _{0}^{\sqrt{\mu }} {\text {d}}p_1 \int _{p_1}^{\sqrt{2\mu }} {\text {d}}x \frac{1}{\vert x-\sqrt{\mu } \vert ^{2/3} (x+\sqrt{\mu })^{2/3}} \\  &\quad \times \left( \frac{1}{(x^2-p_1^2)^{1/3}}+\frac{1}{(\mu -p_1^2)^{1/3}}\right) . \end{aligned}\end{aligned}$$

The integral is bounded by

4.107 $$\begin{aligned} \sqrt{\mu } \int _{0}^{\sqrt{2}} {\textrm{d}}x \int _{0}^{x} {\textrm{d}}p_1 \frac{1}{\vert x-1 \vert ^{2/3}}\left( \frac{1}{x^{1/3}(x-p_1)^{1/3}}+\frac{1}{(1-p_1)^{1/3}}\right) <\infty .\nonumber \\ \end{aligned}$$

Hence, the first two lines in (4.104) are of order $O(1/\lambda )$ by Lemma 3.2. For the third line we carry out the *r*-integration and obtain

4.108 $$\begin{aligned} 2 \int _0^{\sqrt{\mu }}\left( \int _{p_1}^{\sqrt{2\mu }} B_{T_c^0(\lambda )} (x,0) x {\textrm{d}}x \right) ^2 \left( \int _{{\mathbb {S}}^1} \int _{{\mathbb {S}}^1}{\widehat{V}}\left( 0,\sqrt{\mu -p_1^2} (w-w')\right) {\textrm{d}}w{\textrm{d}}w'\right) {\textrm{d}}p_1. \end{aligned}$$

Note that $\int _{p_1}^{\sqrt{2\mu }} B_{T_c^0(\lambda )} (x,0) x {\textrm{d}}x =m_\mu ^{d=2}(T_c^0(\lambda )) -\int _{0}^{p_1} B_{T_c^0(\lambda )} (x,0) x {\textrm{d}}x$ and

4.109 $$\begin{aligned} \int _{0}^{p_1} B_{T_c^0(\lambda )} (x,0) x {\textrm{d}}x= \frac{1}{2} \int _{(\mu -p_1^2)/T_c^0(\lambda )}^{\mu /T_c^0(\lambda )} \frac{\tanh s}{s } {\textrm{d}}s \le \frac{1}{2} \ln \frac{\mu }{\mu -p_1^2},\nonumber \\ \end{aligned}$$

where we substituted $s=(\mu -x^2)/T_c^0(\lambda )$. In particular,

4.110 $$\begin{aligned}  &   \Bigg \vert 2 \int _0^{\sqrt{\mu }}\left[ \left( \int _{p_1}^{\sqrt{2\mu }} B_{T_c^0(\lambda )} (x,0) x {\textrm{d}}x \right) ^2 -m_\mu ^{d=2}(T_c^0(\lambda )) ^2\right] \nonumber \\  &   \qquad \times \left( \int _{{\mathbb {S}}^1} \int _{{\mathbb {S}}^1}{\widehat{V}}\left( 0,\sqrt{\mu -p_1^2} (w-w')\right) {\textrm{d}}w{\textrm{d}}w'\right) {\textrm{d}}p_1 \Bigg \vert \nonumber \\  &   \quad \le 2|{\mathbb {S}}^1|^2 \Vert {\widehat{V}} \Vert _\infty \int _0^{\sqrt{\mu }}\left( \frac{1}{4} \left( \ln \frac{\mu }{\mu -p_1^2}\right) ^2+\ln \frac{\mu }{\mu -p_1^2} m_\mu ^{d=2}(T_c^0(\lambda ))\right) {\textrm{d}}p_1\nonumber \\  &   \quad \le C(1+m_\mu ^{d=2}(T_c^0(\lambda ))), \end{aligned}$$

which is of order $O(1/\lambda )$ by Lemma 3.2. In total, we thus obtain

4.111 $$\begin{aligned}&\lim _{\lambda \rightarrow 0} \lambda ^2\langle V^{1/2}j_3, D_{T_c^0(\lambda )} V^{1/2}j_3 \rangle \nonumber \\&\quad =\lim _{\lambda \rightarrow 0}2\lambda ^2 m_\mu ^{d=2}(T_c^0) ^2 \sqrt{\mu }e_\mu ^2 \int _0^{1}\left( \int _{{\mathbb {S}}^1} \int _{{\mathbb {S}}^1}{\widehat{V}}\left( 0,\sqrt{\mu }\sqrt{1-p_1^2} (w-w')\right) {\textrm{d}}w{\textrm{d}}w'\right) {\textrm{d}}p_1. \end{aligned}$$

By writing out the definition of $j_3$ and then switching to spherical coordinates and carrying out the *r* integration, we have

4.112 $$\begin{aligned}&\int _{{\mathbb {R}}^4} V(r) j_3(z_1,{\tilde{r}};\mu )^2 {\textrm{d}}r {\textrm{d}}z_1\nonumber \\&\quad =\int _{{\mathbb {S}}^2} {\textrm{d}}u \int _{{\mathbb {S}}^2} {\textrm{d}}v \int _{{\mathbb {R}}^7} {\textrm{d}}p {\textrm{d}}r {\textrm{d}}z_1\frac{e^{i p \cdot r}\widehat{V}(p)}{(2\pi )^{3/2}} \frac{e^{i\sqrt{\mu } (z_1,{\tilde{r}})\cdot (u-v)}}{(2\pi )^3}\nonumber \\&\quad = \frac{1}{(2\pi )^{3/2}}\int _{\mathbb {R}}\left( \int _0^\pi \sin \theta {\textrm{d}}\theta \int _0^\pi \sin \theta ' {\textrm{d}}\theta ' \int _{{\mathbb {S}}^1} {\textrm{d}}w \int _{{\mathbb {S}}^1} {\textrm{d}}w'\right. \nonumber \\&\qquad \times \left. \widehat{V}(0,\sqrt{\mu } (\sin \theta w-\sin \theta ' w') e^{i \sqrt{\mu } z_1(\cos \theta -\cos \theta ')}\right) {\textrm{d}}z_1\nonumber \\&\quad =\frac{1}{\sqrt{\mu }(2\pi )^{1/2}} \int _{-1}^1 {\textrm{d}}t \int _{-1}^1{\textrm{d}}s \int _{{\mathbb {S}}^1} {\textrm{d}}w \int _{{\mathbb {S}}^1} {\textrm{d}}w'\nonumber \\&\qquad \times \widehat{V}(0,\sqrt{\mu } (\sqrt{1-t^2} w-\sqrt{1-s^2} w')\delta (s-t), \end{aligned}$$

where in the last step we substituted $t=\cos \theta , s=\cos \theta '$ and carried out the $z_1$ integration. Furthermore, according to Lemma 3.2, $\lim _{\lambda \rightarrow 0} \lambda m_\mu ^{d=2}(T_c^0) e_\mu = \frac{1}{\sqrt{\mu }}$. This gives the desired

4.113 $$\begin{aligned} \lim _{\lambda \rightarrow 0} \lambda ^2\langle V^{1/2}j_3, D_{T_c^0(\lambda )} V^{1/2}j_3 \rangle = (2\pi )^{1/2}\int _{{\mathbb {R}}^4} V(r) j_3(z_1,{\tilde{r}};\mu )^2 {\textrm{d}}r {\textrm{d}}z_1.\nonumber \\ \end{aligned}$$

$$\square $$

## Boundary Superconductivity in 3d

In this section we shall prove Theorem 1.4, which provides sufficient conditions for (1.7) to hold. Due to rotation invariance, we consider the spherical average of ${\tilde{m}}^{D/N}_3$ (defined in (1.6)). With

5.1 $$\begin{aligned} m^{D/N}_3(\vert r\vert ;\mu ):=\frac{1}{4\pi }\int _{{\mathbb {S}}^2} {\tilde{m}}^{D/N}_3(\vert r\vert \omega ;\mu ) {\textrm{d}}\omega , \end{aligned}$$

we have $\int _{{\mathbb {R}}^3} V(r) {\tilde{m}}^{D/N}_3(r;\mu ){\textrm{d}}r =\int _{{\mathbb {R}}^3} V(r) m^{D/N}_3(\vert r\vert ;\mu ) {\textrm{d}}r$. Furthermore, we have the scaling property

5.2 $$\begin{aligned} m_3^{D/N}(\vert r\vert ;\mu )=\frac{1}{\sqrt{\mu }} m_3^{D/N}(\sqrt{\mu }\vert r\vert ;1). \end{aligned}$$

We shall derive the following, more explicit, expression for $m^{D/N}_3$ in Sect. 5.1:

### Lemma 5.1

For $x\ge 0$ we can write $m^{D}_3(x;1)=\sum _{j=1}^4 t_j(x)$ and $m^{N}_3(x;1)=\sum _{j=1}^2 t_j(x)-\sum _{j=3}^4t_j(x)$, where

$$\begin{aligned} t_1(x)&=\frac{4}{\pi x} \int _1^\infty \frac{\sin ^2(x k)}{k} {{\,\textrm{arcoth}\,}}(k) {\textrm{d}}k\\ t_2(x)&=- \frac{2}{\pi } \frac{\sin ^2(x)}{x}\\ t_3(x)&=- 2\frac{\sin ^2( x)}{x^2}\\ t_4(x)&= \frac{4\sin x}{ \pi x^2}\left( \sin x{{\,\textrm{Si}\,}}2 x-\cos x {{\,\textrm{Cin}\,}}2 x\right) \\&= \frac{\sin x}{2\pi ^{3} x}\int _{{\mathbb {S}}^2}\int _{{\mathbb {S}}^{2}} \frac{ \sin (x \omega _1 \vert \omega '_1 \vert ) e^{-i x \tilde{\omega }\cdot \tilde{\omega }' }}{\omega _1} {\textrm{d}}\omega {\textrm{d}}\omega ', \end{aligned}$$

with $${{\,\textrm{Cin}\,}}(x)=\int _0^x \frac{1-\cos t}{t} {\textrm{d}}t $$ and $${{\,\textrm{Si}\,}}(x)=\int _0^x \frac{\sin t}{t} {\textrm{d}}t$$.

**Fig. 2:**
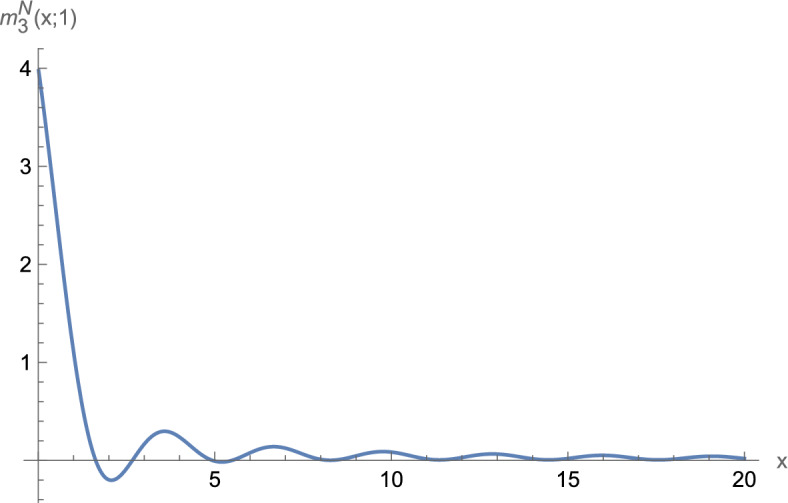
Plot of $m_3^N$ for $\mu =1$, created using [17]

To determine for which interactions $\int _{{\mathbb {R}}^3} V(r) m^{D/N}_3(\vert r\vert ;\mu ){\textrm{d}}r >0$ holds, we need to understand $m^{D/N}_3(\vert r\vert ;\mu )$. In Figs. 1 and 2 we plot $m_3^D$ and $m_3^N$ for $\mu =1$, respectively.

The function $m_3^D$ seems to be nonnegative. If one could prove that $m_3^D\ge 0$, then Theorem 1.3 would apply to all $V\ge 0$ satisfying Assumption 1.1. Unfortunately, this is beyond our reach. On the other hand, the function $m_3^N$ changes sign, but is positive in a neighborhood of zero.

### Remark 5.2

To create the plots, it is computationally more efficient to use the first expression for $t_4$, whereas for the following analytic computations the second expression is more convenient.

Intuitively, if we let $\mu \rightarrow 0$, due to the scaling (5.2) the sign of $\int _{{\mathbb {R}}^3} V(r) m^{D/N}_3(\vert r\vert ;\mu ){\textrm{d}}r$ is determined by the values of $m_3^{D/N}(\vert r\vert ;1)$ for *r* in the vicinity of zero. To obtain Theorem 1.4, we prove that both functions $m_3^{D/N}(\vert r\vert ;1)$ are non-negative in a neighborhood of zero.

The following is proven in Sect. 5.2:

### Lemma 5.3

The functions $t_j$ for $j=1,2,3,4$ are bounded and twice continuously differentiable. The values of the functions and their derivatives at zero are listed in Table 1.

**Table 1:** Values of the functions $t_j$ and $m^{D/N}_3$ and their derivatives at zero. The missing entries are not needed

*f*	$t_1$	$t_2$	$t_3$	$t_4$	$m^D_3(\cdot ;1)$	$m^N_3(\cdot ;1)$
*f*(0)	2	0	$-2$	0	0	4
$f' (0)$	$- 2/\pi$	$- 2/\pi$	0	$4/\pi$	0	
$f'' (0)$	$-8/9$	0	4/3	0	4/9	

### Proof of Theorem 1.4

We start with the case of Neumann boundary condition. By (5.2), it suffices to prove that $\lim _{\mu \rightarrow 0} \int _{{\mathbb {R}}^3} V(r) m_3^{N}(\sqrt{\mu }\vert r\vert ;1){\textrm{d}}r>0$. With $V\in L^1$ and Lemma 5.3 it follows by dominated convergence that

$$\begin{aligned} \lim _{\mu \rightarrow 0} \int _{{\mathbb {R}}^3} V(r) m_3^{N}(\sqrt{\mu }\vert r\vert ;1){\textrm{d}}r=m_3^{N}(0;1) \int _{{\mathbb {R}}^3} V(r) {\textrm{d}}r=4 \int _{{\mathbb {R}}^3} V(r) {\textrm{d}}r. \end{aligned}$$

Since ${\widehat{V}}(0)>0$ by assumption, this is positive.

For Dirichlet boundary conditions, according to Lemma 5.3, $m_3^{D}(0;1)$ and its first derivative vanish. Thus, we consider $I(\sqrt{\mu }):=\frac{1}{\mu }\int _{{\mathbb {R}}^3} m_3^D(\sqrt{\mu } \vert r\vert ;1) V(r) {\textrm{d}}r$. Since $m_3^D(\cdot ;1)$ is bounded, *I* is continuous away from 0. It suffices to prove that $\lim _{\mu \rightarrow 0} I(\sqrt{\mu })>0$. According to Lemma 5.3 and Taylor’s theorem, we have $m_3^D(x;1) =\frac{1}{2} (m_3^D)''(0;1) x^2+R(x)$, where *R* is continuous with $\lim _{x\rightarrow 0}\frac{\vert R(x)\vert }{x^2} =0$. Let $\epsilon >0$ and $c:=\sup _{0\le x< \epsilon } \frac{\vert R(x)\vert }{x^2}<\infty$. One can bound

5.3 $$\begin{aligned} \left| \frac{1}{\mu }m_3^D(\sqrt{\mu } \vert r\vert ;1) V(r) \right|&\le \chi _{\sqrt{\mu } \vert r \vert <\epsilon } \Big (\frac{1}{2} (m_3^D)''(0;1)+c\Big ) \vert |r|^2 V(r) \vert \nonumber \\&\quad + \chi _{\sqrt{\mu } \vert r \vert >\epsilon } \frac{\Vert m_3^D \Vert _\infty }{\epsilon ^2} \vert |r|^2 V(r) \vert \nonumber \\&\le \Big (\frac{1}{2} (m_3^D) ''(0;1)+c+\frac{\Vert m_3^D \Vert _\infty }{\epsilon ^2} \Big ) \vert |r|^2 V(r) \vert , \end{aligned}$$

which is integrable by the assumptions on *V*. By dominated convergence

5.4 $$\begin{aligned} \lim _{\mu \rightarrow 0} I(\sqrt{\mu })= &   \int _{{\mathbb {R}}^3} \lim _{\mu \rightarrow 0} \frac{m_3^D(\sqrt{\mu } \vert r\vert ;1) }{\mu \vert r\vert ^2} V(r) \vert r \vert ^2 {\textrm{d}}r\nonumber \\= &   \frac{1}{2}\int _{{\mathbb {R}}^3} (m_3^D)''(0;1) V(r) \vert r \vert ^2 {\textrm{d}}r\nonumber \\= &   \frac{2}{9}\int _{{\mathbb {R}}^3} V(r) \vert r \vert ^2 {\textrm{d}}r, \end{aligned}$$

which is positive by assumption. $\square$

### Proof of Lemma 5.1

#### Proof of Lemma 5.1

With

$$\begin{aligned} {\tilde{t}}_1(r)&=\int _{{\mathbb {R}}} j_3(z_1,r_2,r_3;1)^2 \chi _{|z_1|>|r_1|} {\textrm{d}}z_1 \\ {\tilde{t}}_2(r)&=- j_3(r;1)^2 \int _{{\mathbb {R}}} \chi _{|z_1|<|r_1|}{\textrm{d}}z_1 \\ {\tilde{t}}_3(r)&= \mp \pi j_3(r;1)^2\\ {\tilde{t}}_4(r)&=\pm 2 j_3(r;1) \int _{{\mathbb {R}}} j_3(z_1,r_2,r_3;1)\chi _{|z_1|<|r_1|}{\textrm{d}}z_1, \end{aligned}$$

one can write ${\widetilde{m}}^{D}_3(r;1)=\sum _{j=1}^4 {\tilde{t}}_j(r)$ and ${\widetilde{m}}^{N}_3(r;1)=\sum _{j=1}^2 {\tilde{t}}_j(r)-\sum _{j=3}^4 {\tilde{t}}_j(r)$. Let $t_j(\vert r\vert )=\frac{1}{4\pi }\int _{{\mathbb {S}}^2} {\tilde{t}}_j^{D/N}(\vert r\vert \omega ;\mu ) {\textrm{d}}\omega .$ The following explicit computations show that the $t_j$ agree with the claimed expressions.

Recall that $j_3(r;1)=\sqrt{\frac{2}{\pi }} \frac{\sin \vert r \vert }{ \vert r \vert }$. For $t_1$ we write out the integral in spherical coordinates and substitute $z_1=x y$ and $s=\cos \theta$

5.5 $$\begin{aligned} t_1(x)= &   \frac{1}{\pi } \frac{2\pi }{4\pi } \int _{0}^\pi \int _{{\mathbb {R}}} \frac{\sin ^2 \sqrt{z_1^2+(x \sin \theta )^2}}{ z_1^2+(x \sin \theta )^2 } \chi _{|z_1|>x| \cos \theta |} \sin \theta {\textrm{d}}z_1 {\textrm{d}}\theta \nonumber \\= &   \frac{1}{\pi x} \int _{-1}^1 \int _{{\mathbb {R}}} \frac{\sin ^2 x \sqrt{y^2+1-s^2}}{y^2+1-s^2} \chi _{|y|>\vert s\vert } {\textrm{d}}y {\textrm{d}}s. \end{aligned}$$

Next, we use the reflection symmetry of the integrand in *s* and *y*, substitute *y* by $k=\sqrt{y^2+1-s^2}$ and then carry out the *s* integration to obtain

5.6 $$\begin{aligned} t_1(x)= \frac{4}{\pi x} \int _{0}^1 \int _{1}^\infty \frac{\sin ^2 x k}{k \sqrt{k^2+s^2-1}} {\textrm{d}}k {\textrm{d}}s = \frac{4}{\pi x} \int _{1}^\infty \frac{\sin ^2 x k}{k } {{\,\textrm{arcoth}\,}}(k) {\textrm{d}}k.\nonumber \\ \end{aligned}$$

For $t_2$, we have

5.7 $$\begin{aligned} t_2(x)=- \frac{2}{\pi } \frac{\sin ^2 x }{ x^2} \frac{1}{4\pi } \int _{{\mathbb {S}}^2} 2 x \vert \omega _1 \vert {\textrm{d}}\omega =- \frac{2}{\pi } \frac{\sin ^2 x}{ x}. \end{aligned}$$

Since ${\tilde{t}}_3$ is radial, we have $t_3={\tilde{t}}_3$. For $t_4$ we want to derive two expressions. For the first, we perform the same substitutions as for $t_1$

5.8 $$\begin{aligned} t_4(x)= &   \frac{4}{\pi } \frac{\sin x}{ x}\frac{2\pi }{4\pi } \int _{0}^\pi \int _{{\mathbb {R}}} \frac{\sin \sqrt{z_1^2+(x \sin \theta )^2}}{ \sqrt{ z_1^2+(x \sin \theta )^2 }} \chi _{|z_1|<x| \cos \theta |} \sin \theta {\textrm{d}}z_1 {\textrm{d}}\theta \nonumber \\= &   \frac{2}{\pi } \frac{\sin x}{ x} \int _{-1}^1 \int _{{\mathbb {R}}} \frac{\sin x \sqrt{y^2+1-s^2}}{ \sqrt{y^2+1-s^2}} \chi _{|y|<| s|} {\textrm{d}}y {\textrm{d}}s \nonumber \\= &   \frac{8}{\pi } \frac{\sin x}{ x} \int _{0}^1 \int _{0}^1 \frac{\sin x k}{\sqrt{k^2+s^2-1}} \chi _{k^2+s^2>1}{\textrm{d}}k {\textrm{d}}s\nonumber \\= &   \frac{8}{\pi } \frac{\sin x}{ x} \int _{0}^1\sin x k {{\,\textrm{artanh}\,}}k {\textrm{d}}k \nonumber \\= &   \frac{4\sin x}{ \pi x^2}\left( \sin x {{\,\textrm{Si}\,}}2 x-\cos x {{\,\textrm{Cin}\,}}2 x \right) . \end{aligned}$$

To obtain the second expression for $t_4$, note that $\int _{\mathbb {R}}e^{-i \omega _1 z_1} \chi _{\vert z_1 \vert <\vert r_1 \vert } {\textrm{d}}z_1=\frac{2 \sin \omega _1 |r_1|}{ \omega _1}$. Therefore,

5.9 $$\begin{aligned} t_4(x)= &   2 \sqrt{\frac{2}{\pi }} \frac{\sin x}{ x} \frac{1}{4\pi }\int _{{\mathbb {S}}^2} \int _{\mathbb {R}}\int _{{\mathbb {S}}^2}\frac{e^{-i \omega \cdot (z_1,x \tilde{\omega }' )}}{(2\pi )^{3/2}} \chi _{\vert z_1 \vert <x \vert \omega _1' \vert }{\textrm{d}}\omega {\textrm{d}}z_1 {\textrm{d}}\omega ' \nonumber \\= &   \frac{1}{2\pi ^{3}} \frac{\sin x}{ x}\int _{{\mathbb {S}}^2} \int _{{\mathbb {S}}^2}\frac{ \sin x \omega _1 \vert \omega _1'\vert }{ \omega _1} e^{-i x \tilde{\omega }\cdot \tilde{\omega }' }{\textrm{d}}\omega {\textrm{d}}\omega '. \end{aligned}$$

$$\square $$

### Proof of Lemma 5.3

#### Proof of Lemma 5.3

Since $\sin (x)/x$ is a bounded and smooth function, also $t_2$ and $t_3$ are bounded and smooth. Elementary computations give the entries in Table 1.

For $t_4$ use the second expression in Lemma 5.1. Since the integrand is bounded and smooth and the domain of integration is compact, the integral is bounded and we can exchange integration and taking limits and derivatives. In particular, $t_4$ is bounded and smooth and it is then an elementary computation to verify the entries in Table 1. For instance,

5.10 $$\begin{aligned} t_4'(0)=\frac{1}{2 \pi ^3} \int _{{\mathbb {S}}^2}\int _{{\mathbb {S}}^{2}} \vert \omega _1' \vert {\textrm{d}}\omega {\textrm{d}}\omega '= \frac{4}{\pi }. \end{aligned}$$

To study $t_1$ we define auxiliary functions $f(x)=\frac{4}{\pi x} {{\,\textrm{artanh}\,}}(x)$ and $g(x)=\frac{\sin (x)^2}{x^2}$. Note that *f*(*x*) diverges logarithmically for $x\rightarrow 1$ and is continuous otherwise with $f(0)=\frac{4}{\pi }$. Furthermore, *f*(*x*) is increasing on [0, 1) and for every $0<\epsilon <1$, $\sup _{0\le x<\epsilon } \frac{f'(x)}{x}=\frac{f'(\epsilon )}{\epsilon }<\infty$ since all coefficients in the Taylor series of ${{\,\textrm{artanh}\,}}(x)$ are positive.

We can write

5.11 $$\begin{aligned} t_1(x)=\int _1^\infty x g(x k) f(1/k) {\textrm{d}}k =\int _1^c x g(x k) f(1/k) {\textrm{d}}k +\int _{c x}^\infty g(k) f(x/k) {\textrm{d}}k\nonumber \\ \end{aligned}$$

for any constant $c>1$. The first integrand is bounded by $Cx {{\,\textrm{arcoth}\,}}(k)$, the second one by $C\frac{1}{k^2}$ (since *f* is bounded on the integration domain). By dominated convergence we obtain that $t_1$ is continuous and $t_1(0)=\frac{4}{\pi } \int _0^\infty g(k) {\textrm{d}}k =2$.

For $x>0$, we compute the derivative

5.12 $$\begin{aligned} t_1'(x)&=\int _1^c (g(x k)+x k g'(x k)) f(1/k) {\textrm{d}}k -cg(cx)f(1/c)+\int _{c x}^\infty g(k) f'(x/k) \frac{1}{k}{\textrm{d}}k\nonumber \\&=\int _1^c (g(x k) +x k g'(xk))f(1/k) {\textrm{d}}k -cg(cx)f(1/c)+\int _{c}^\infty g(k x) f'(1/k) \frac{1}{k}{\textrm{d}}k, \end{aligned}$$

where we could apply the Leibnitz integral rule since $$f'(1/k)$$ decays like 1/*k* for $$k\rightarrow \infty $$. By dominated convergence, $$ t_1'$$ is continuous for $$x>0$$. By continuity of $$t_1$$ and the mean value theorem, $$t_1'(0)=\lim _{x\rightarrow 0}\frac{t_1(x)-t_1(0)}{x}=\lim _{x\rightarrow 0}\lim _{y\rightarrow 0}\frac{t_1(x)-t_1(y)}{x-y}=\lim _{x\rightarrow 0}t_1'(x)$$. We evaluate

5.13 $$\begin{aligned} t_1'(0)= &   \int _1^c f(1/k) {\textrm{d}}k -c f(1/c)+\int _{c}^\infty f'(1/k) \frac{1}{k}{\textrm{d}}k\nonumber \\= &   \int _1^c \left( f(1/k) -f(1/c)\right) {\textrm{d}}k-f(1/c)+\int _{c}^\infty f'(1/k) \frac{1}{k}{\textrm{d}}k. \end{aligned}$$

This is a number independent of *c*. To compute the number, we let $c\rightarrow \infty$, and by monotone convergence

5.14 $$\begin{aligned} t_1'(0)=\int _1^\infty \left( f(1/k) -f(0)\right) {\textrm{d}}k-f(0)=\frac{2}{\pi }-\frac{4}{\pi }=-\frac{2}{\pi }. \end{aligned}$$

Note that $g'(k)=2(\cos k -\frac{\sin k}{k})\frac{\sin k}{k^2}$ has a zero of order one at $k=0$. Therefore, $\left| g'(k x) f'(1/k)\right| <\frac{C}{x^2 k^3}$ and for $x>0$ the second derivative is

5.15 $$\begin{aligned} t_1''(x)= &   \int _1^c (2x g'(x k)+x k^2 g''(x k)) f(1/k) {\textrm{d}}k -c^2 g'(cx)f(1/c)\nonumber \\  &   +\int _{c}^\infty g'(k x) f'(1/k){\textrm{d}}k\nonumber \\= &   \int _1^c (2 x g'(x k)+x k^2 g''(x k)) f(1/k) {\textrm{d}}k -c^2 g'(cx)f(1/c)\nonumber \\  &   +\int _{cx}^\infty \frac{g'(y)}{y} \frac{f'(x/y)}{x/y}{\textrm{d}}y. \end{aligned}$$

We can bound $\frac{g'(y)}{y}\le \frac{C}{1+y^3}$ and $\sup _y |\frac{f'(x/y)}{x/y}\chi _{y>cx}|=c f'(1/c)<\infty$. By dominated convergence, the function above is continuous (also at zero). We have

5.16 $$\begin{aligned} t_1''(0)= \int _{0}^\infty \frac{g'(y)}{y} {\textrm{d}}y \lim _{x\rightarrow 0} \frac{f'(x)}{x}. \end{aligned}$$

Since $\int _{0}^\infty \frac{g'(y)}{y} {\textrm{d}}y =-\frac{\pi }{3}$ and $\lim _{x\rightarrow 0} \frac{f'(x)}{x}=\frac{8}{3\pi }$ we obtain

5.17 $$\begin{aligned} t_1''(0)=-\frac{8}{9}. \end{aligned}$$

$$\square $$

## Relative Temperature Shift

In this section we shall prove Theorem 1.7, which states that the relative temperature shift vanishes in the weak coupling limit. We proceed similarly to the $\delta$-interaction case in one dimension analyzed in [6]. For this, we switch to the Birman–Schwinger formulation. Let $\tilde{\Omega }_1=\{(r,z)\in {\mathbb {R}}^{2d}\vert \vert r_1 \vert < z_1 \}$. Define the operator $A_T^1$ on $\psi \in L_\textrm{s}^2(\tilde{\Omega }_1)=\{\psi \in L^2(\tilde{\Omega }_1) \vert \psi (r,z)=\psi (-r,z)\}$ via

6.1 $$\begin{aligned} \begin{aligned} \langle \psi , A_{T}^1 \psi \rangle&=\int _{{\mathbb {R}}^{4d+2(d-1)}} {\text {d}}r {\text {d}}r' {\text {d}}p {\text {d}}q {\text {d}}{\tilde{z}} {\text {d}}{\tilde{z}}' \int _{\vert r_1 \vert<z_1} {\text {d}}z_1\int _{\vert r_1 '\vert <z_1'} {\text {d}}z'_1 \frac{1}{(2\pi )^{2d}} \\  &\quad \times \overline{\psi (r,z)} V(r)^{1/2} e^{i(p \cdot z+q \cdot r)} B_{T}\left( p,q\right) \\  &\quad \times \Bigg (e^{-i(p_1 z'_1+q_1 r'_1)}+e^{i(p_1 z'_1+q_1 r'_1)}\mp e^{-i(q_1 z'_1+p_1 r'_1)}\mp e^{i(q_1 z'_1+p_1 r'_1)}\Bigg ) \\  &\quad \times e^{-i({\tilde{p}} \cdot {\tilde{z}}'+{\tilde{q}} \cdot {\tilde{r}}')}\vert V(r')\vert ^{1/2}\psi (r',z'), \end{aligned} \end{aligned}$$

where the upper signs correspond to Dirichlet and the lower signs to Neumann boundary conditions, respectively. It follows from a computation analogous to [6, Lemma 2.4] that the operator $A_T^1$ is the Birman–Schwinger operator corresponding to $H_T^{\Omega _1}$ in relative and center of mass variables. The Birman–Schwinger principle implies that $\textrm{sgn}\inf \sigma (H_T^{\Omega _1}) = \textrm{sgn}(1/\lambda -\sup \sigma (A_T^{1}))$, where we use the convention that $\textrm{sgn}\, 0 =0$.

Recall the Birman-Schwinger operator $A_T^0$ corresponding to $H_T^0$ from (3.1). Similarly, the Birman-Schwinger operator $A_{T}^{\Omega _0}$ corresponding to $H_T^{\Omega _0}$ in relative and center of mass variables is defined on $\psi (r,z)\in L^2({\mathbb {R}}^d\times {\mathbb {R}}^d)$ with $\psi (r,z)=\psi (-r,z)$ and satisfies

6.2 $$\begin{aligned} \langle \psi , A_{T}^{\Omega _0} \psi \rangle= &   \int _{{\mathbb {R}}^{6d}} {\textrm{d}}r {\textrm{d}}r' {\textrm{d}}p {\textrm{d}}q {\textrm{d}}z {\textrm{d}}z' \overline{\psi (r,z)} V(r)^{1/2} \frac{e^{i(p \cdot (z-z')+q \cdot (r-r'))}}{(2\pi )^{2d}}\nonumber \\  &   \times B_{T}\left( p,q\right) \vert V(r')\vert ^{1/2}\psi (r',z'). \end{aligned}$$

Let $a_T^j=\sup \sigma (A_T^j)$. Let us first observe that there is a $T_0>0$ such that $a_T^{\Omega _0}=a_T^0$ for $T<T_0$. Let $\lambda _0>0$ such that $T_c^{\Omega _0}(\lambda )=T_c^0(\lambda )$ for $\lambda \le \lambda _0$, see Remark 2.5. Choose $T_0=T_c^{\Omega _0}(\lambda _0)=T_c^0(\lambda _0)$ and let $T<T_0$. Due to strict monotonicity of $H_T^0$ in *T*, $T=T_c^0(\lambda )$ for some $\lambda <\lambda _0$. By choice of $\lambda _0$ also $T_c^{\Omega _0}(\lambda )=T$. The Birman-Schwinger principle implies $a_T^{\Omega _0}=\lambda =a_T^0$.

For $T\rightarrow 0$, the asymptotics of $a_T^{\Omega _0}$ thus agrees with the asymptotics of $a_T^0$, i.e. $a_T^{\Omega _0}=e_\mu \mu ^{d/2-1}\ln (\mu /T) +O(1)$ [8, Theorem 3.3] and [9, Theorem 2.5]. One can reformulate the claim of Theorem 1.7 in terms of the Birman-Schwinger operators. Then

6.3 $$\begin{aligned} \lim _{\lambda \rightarrow 0} \frac{T_c^{\Omega _1}(\lambda )-T_c^{\Omega _0}(\lambda )}{T_c^{\Omega _0}(\lambda )} =0 \Leftrightarrow \lim _{T\rightarrow 0} \left( a_T^{\Omega _0}-a_T^1\right) =0. \end{aligned}$$

This is a straightforward generalization of [6, Lemma 4.1] and we refer to [6, Lemma 4.1] for its proof.

### Proof of Theorem 1.7

First we will argue that $a_T^{\Omega _0}\le a_T^1$. If $\inf \sigma (K_T^{\Omega _0}-\lambda V)<2T$, then $\inf \sigma (K_T^{\Omega _0}-\lambda ' V)<\inf \sigma (K_T^{\Omega _0}-\lambda V)$ for all $\lambda '>\lambda$. Furthermore, $\inf \sigma (K_T^{\Omega _0}-(a_T^{\Omega _0})^{-1}V)=0=\inf \sigma (K_T^{\Omega _1}-(a_T^1)^{-1}V)\le \inf \sigma (K_T^{\Omega _0}-(a_T^1)^{-1}V)$, where we used Lemma 2.3 in the last step. In particular, $a_T^{\Omega _0}\le a_T^1$.

It remains to show that $\lim _{T\rightarrow 0} \left( a_T^{\Omega _0}-a_T^1\right) \ge 0$. Let $\iota : L^2(\tilde{\Omega }_1)\rightarrow L^2({\mathbb {R}}^{2d})$ be the isometry

6.4 $$\begin{aligned} \begin{aligned} \iota \psi (r_1,{\tilde{r}},z_1,{\tilde{z}})=&\frac{1}{\sqrt{2}} (\psi (r_1,{\tilde{r}},z_1,{\tilde{z}}) \chi _{\tilde{\Omega }_1}(r,z) \\  &+\psi (-r_1,{\tilde{r}},-z_1,{\tilde{z}}) \chi _{\tilde{\Omega }_1}(-r_1,{\tilde{r}},-z_1,{\tilde{z}})). \end{aligned} \end{aligned}$$

Let $F_2$ denote the Fourier transform in the second variable $F_2 \psi (r, q)=\frac{1}{(2\pi )^{d/2}} \int _{{\mathbb {R}}^d} e^{-iq\cdot z} \psi (r,z) {\textrm{d}}z$ and $F_1$ the Fourier transform in the first variable $F_1 \psi (p,q)=\frac{1}{(2\pi )^{d/2}} \int _{{\mathbb {R}}^d} e^{-ip\cdot r} \psi (r,q) {\textrm{d}}r.$ Recall that by assumption $V\ge 0$ and for functions $\psi \in L^2({\mathbb {R}}^d\times {\mathbb {R}}^d)$ we have $V^{1/2}\psi (r,q)=V^{1/2}(r)\psi (r,q)$. We define self-adjoint operators ${\tilde{E}}_T$ and $G_T$ on $L^2({\mathbb {R}}^{2d})$ through

6.5 $$\begin{aligned} \langle \psi , {\tilde{E}}_{T} \psi \rangle = a_T^{\Omega _0}\Vert \psi \Vert _2^2 -\int _{{\mathbb {R}}^{2d}} B_{T}(p,q) |F_1 V^{1/2}\psi (p,q)|^2{\textrm{d}}p {\textrm{d}}q \end{aligned}$$

and

6.6 $$\begin{aligned} \langle \psi , G_{T} \psi \rangle =\int _{{\mathbb {R}}^{2d}} \overline{F_1 V^{1/2}\psi ((q_1,{\tilde{p}}),(p_1,{\tilde{q}}))} B_{T}(p,q) F_1 V^{1/2}\psi (p,q) {\textrm{d}}p {\textrm{d}}q.\nonumber \\ \end{aligned}$$

With this notation, we have $a_T^{\Omega _0}{\mathbb {I}}-A_T^1=\iota ^\dagger F_2^\dagger ({\tilde{E}}_T\pm G_T) F_2 \iota$, where ${\mathbb {I}}$ denotes the identity operator on $L_\textrm{s}^2(\tilde{\Omega }_1)$. In particular,

6.7 $$\begin{aligned} a_T^{\Omega _0}-a_T^1= &   \inf _{\psi \in L^2_\textrm{s}(\tilde{\Omega }_1), \Vert \psi \Vert _2=1} \langle F_2\iota \psi ,({\tilde{E}}_{T}\pm G_{T} )F_2\iota \psi \rangle \nonumber \\\ge &   \inf _{ \psi \in L^2_\textrm{s}({\mathbb {R}}^{2d}), \Vert \psi \Vert _2=1} \langle \psi ,({\tilde{E}}_{T}\pm G_{T}) \psi \rangle , \end{aligned}$$

where we used that $\Vert F_2 \iota \psi \Vert _2= \Vert \psi \Vert _2$. Define the function

6.8 $$\begin{aligned} E_{T}(q)= a_T^{\Omega _0}-\Vert V^{1/2}B_{T}(\cdot ,q) V^{1/2} \Vert _{\text {s}}, \end{aligned}$$

where $$\Vert \cdot \Vert _{\text {s}}$$ denotes the operator norm of the operator restricted to even functions. Since $$a_T^{\Omega _0}=\sup _q \Vert V^{1/2}B_{T}(\cdot ,q) V^{1/2} \Vert _{\text {s}}$$, we have $$E_T(q)\ge 0$$ for all *T*. Let $$ E_{T}$$ act on $$L^2({\mathbb {R}}^{2d})$$ as $$ E_{T} \psi (r,q)= E_{T}(q)\psi (r,q)$$. Then

6.9 $$\begin{aligned} a_T^{\Omega _0}-a_T^1\ge \inf _{ \psi \in L^2_\textrm{s}({\mathbb {R}}^{2d}), \Vert \psi \Vert _2=1} \langle \psi ,( E_{T}\pm G_{T} ) \psi \rangle . \end{aligned}$$

It thus suffices to prove that $\lim _{T\rightarrow 0} \inf \sigma ( E_{T}\pm G_{T})\ge 0$. With the next three Lemmas, which are proved in the next sections, the claim follows completely analogously to the proof of [6, Theorem 1.2 (ii)]. For completeness, we provide a sketch of the argument in [6, Theorem 1.2 (ii)] after the statement of the Lemmas.

### Lemma 6.1

Let $\mu >0$, $d\in \{1,2,3\}$ and let $V\ge 0$ satisfy Assumption 1.1(i). Then $\sup _{T>0} \Vert G_{T} \Vert <\infty$.

### Lemma 6.2

Let $\mu >0$, $d\in \{1,2,3\}$ and let $V\ge 0$ satisfy Assumption 1.1(i). Let ${\mathbb {I}}_{\le \epsilon }$ act on $L^2({\mathbb {R}}^{2d})$ as ${\mathbb {I}}_{\le \epsilon } \psi (r,p)=\psi (r,p)\chi _{\vert p\vert \le \epsilon }$. Then

$$\begin{aligned} \lim _{\epsilon \rightarrow 0} \sup _{T>0} \Vert {\mathbb {I}}_{\le \epsilon }G_{T} {\mathbb {I}}_{\le \epsilon } \Vert =0. \end{aligned}$$

### Lemma 6.3

Let $\mu >0$, $d\in \{1,2,3\}$ and let $V\ge 0$ satisfy Assumption 1.1. Let $0<\epsilon <\sqrt{\mu }$. There are constants $c_1,c_2,T_1>0$ such that for $0<T<T_1$ and $|q|>\epsilon$ we have $E_{T}(q)>c_1 \vert \ln (c_2/T)\vert$.

Since $E_{T}(q)\ge 0$, we can write

6.10 $$\begin{aligned} E_{T}\pm G_{T}+\delta =\sqrt{E_{T}+\delta }\left( {\mathbb {I}}\pm \frac{1}{\sqrt{E_{T}+\delta }}G_{T}\frac{1}{\sqrt{E_{T}+\delta }}\right) \sqrt{E_{T}+\delta }\nonumber \\ \end{aligned}$$

for any $\delta >0$. It suffices to prove that, for all $\delta >0$,

6.11 $$\begin{aligned} \lim _{T\rightarrow 0} \left\Vert \frac{1}{\sqrt{E_{T}+\delta }}G_{T}\frac{1}{\sqrt{E_{T}+\delta }}\right\Vert =0 . \end{aligned}$$

To prove (6.11), with the notation introduced in Lemma 6.2 we have, for all $0<\epsilon <\sqrt{\mu }$,

6.12 $$\begin{aligned} \left\Vert \frac{1}{\sqrt{E_{T}+\delta }}G_{T}\frac{1}{\sqrt{E_{T}+\delta }}\right\Vert\le &   \left\Vert {\mathbb {I}}_{\le \epsilon } \frac{1}{\sqrt{E_{T}+\delta }}G_{T}\frac{1}{\sqrt{E_{T}+\delta }}{\mathbb {I}}_{\le \epsilon }\right\Vert \nonumber \\  &   +\left\Vert {\mathbb {I}}_{\le \epsilon } \frac{1}{\sqrt{E_{T}+\delta }}G_{T}\frac{1}{\sqrt{E_{T}+\delta }}{\mathbb {I}}_{>\epsilon }\right\Vert \nonumber \\  &   +\left\Vert {\mathbb {I}}_{>\epsilon } \frac{1}{\sqrt{E_{T}+\delta }}G_{T}\frac{1}{\sqrt{E_{T}+\delta }}\right\Vert . \end{aligned}$$

With $E_{T}\ge 0$ and Lemma 6.3 we obtain

6.13 $$\begin{aligned} \lim _{T\rightarrow 0}\left\Vert \frac{1}{\sqrt{E_{T}+\delta }}G_{T}\frac{1}{\sqrt{E_{T}+\delta }}\right\Vert\le &   \sup _{T>0} \frac{1}{\delta }\left\Vert {\mathbb {I}}_{\le \epsilon } G_{T} {\mathbb {I}}_{\le \epsilon }\right\Vert \nonumber \\  &   +\lim _{T\rightarrow 0} \frac{2}{(\delta c_1 \vert \ln (c_2/T)\vert )^{1/2}} \Vert G_{T} \Vert .\qquad \end{aligned}$$

The second term vanishes by Lemma 6.1 and the first term can be made arbitrarily small by Lemma 6.2. Hence (6.11) follows. $\square$

### Remark 6.4

The variational argument above relies on $A_T^1$ being self-adjoint. This is why we assume $V\ge 0$ in Theorem 1.7.

### Proof of Lemma 6.1

#### Proof of Lemma 6.1

We have $\Vert G_T \Vert \le \Vert G_T^< \Vert +\Vert G_T^> \Vert$, where, for $d\in \{2,3\}$,

6.14 $$\begin{aligned} \begin{aligned} \langle \psi , G_{T}^< \psi \rangle =&\int _{{\mathbb {R}}^{2d}} \overline{F_1 V^{1/2}\psi ((q_1,{\tilde{p}}),(p_1,{\tilde{q}}))} B_{T}(p,q)\chi _{|{\tilde{p}}|<2\sqrt{\mu }}\\  &\quad \times F_1 V^{1/2}\psi (p,q) {\text {d}}p {\text {d}}q, \end{aligned} \end{aligned}$$

and for $$ G_{T}^>$$ change $$\chi _{|{\tilde{p}}|<2\sqrt{\mu }}$$ to $$\chi _{|{\tilde{p}}|>2\sqrt{\mu }}$$. For $$d=1$$ set $$G_T^{<}=G_T$$ and $$G_T^>=0$$. We will prove that $$G_T^<$$ and $$G_T^>$$ are bounded uniformly in *T*.

To bound $G_T^>$ in $d=2,3$ we use the Schwarz inequality in $p_1,q_1$ to obtain

6.15 $$\begin{aligned} \Vert G_T^>\Vert \le \sup _{ \psi \in L^2({\mathbb {R}}^{2d}), \Vert \psi \Vert =1} \int _{{\mathbb {R}}^{2d}} B_{T}\left( p,q\right) \chi _{\vert {\tilde{p}}\vert >2 \sqrt{\mu }}\vert F_1 V^{1/2}\psi (p,q) \vert ^2 {\textrm{d}}q {\textrm{d}}p.\nonumber \\ \end{aligned}$$

The right hand side defines a multiplication operator in *q*. By (2.3) there is a constant $C>0$ independent of *T* such that $\Vert G_T^>\Vert \le C \Vert M\Vert$, where $M:= V^{1/2} \frac{1}{1-\Delta } V^{1/2}$ on $L^2({\mathbb {R}}^d)$. It follows from the Hardy-Littlewood-Sobolev and the Hölder inequalities that *M* is a bounded operator [8, 9, 11].

To bound $G_T^<$ note that for fixed *q*, $\Vert F_1 V^{1/2}\psi (\cdot , q )\Vert _\infty \le C \Vert V \Vert _1^{1/2} \Vert \psi (\cdot , q ) \Vert _2$ by Lemma 3.7(iii). Therefore, we estimate

6.16 $$\begin{aligned} \begin{aligned} \Vert G_T^< \Vert \le&C^2 \Vert V \Vert _1 \sup _{ \psi \in L^2({\mathbb {R}}^{2d}), \Vert \psi \Vert =1} \int _{{\mathbb {R}}^{2d}} \Vert \psi (\cdot , (p_1,{\tilde{q}}) ) \Vert _2 B_{T}(p,q)\\  &\times \chi _{{\tilde{p}}^2<2\mu } \Vert \psi (\cdot , q ) \Vert _2{\text {d}}p {\text {d}}q. \end{aligned} \end{aligned}$$

Since the right hand side defines a multiplication operator in ${\tilde{q}}$, we obtain

6.17 $$\begin{aligned} \Vert G_T^< \Vert \le C^2 \Vert V \Vert _1 \sup _{{\tilde{q}} \in {\mathbb {R}}^{d-1}} \sup _{ \psi \in L^2({\mathbb {R}}), \Vert \psi \Vert =1} \int _{{\mathbb {R}}^{d+1}} \overline{ \psi (p_1)} B_{T}(p,q)\chi _{{\tilde{p}}^2<2\mu } \psi (q_1 ) {\textrm{d}}p {\textrm{d}}q_1, \end{aligned}$$

where for $d=1$ the supremum over ${\tilde{q}}$ is absent. For $d=1$, the operator with integral kernel $B_{T}(p,q)$ is bounded uniformly in *T* according to [6, Lemma 4.2], and thus the claim follows. For $d\in \{2,3\}$ we need to prove that the operators with integral kernel $\int _{{\mathbb {R}}^{d-1}} B_{T}(p,q) \chi _{\vert {\tilde{p}} \vert <2 \sqrt{\mu }} {\textrm{d}}{\tilde{p}}$ are bounded uniformly in ${\tilde{q}}$ and *T*. We apply the bound [6, Lemma 4.6]

6.18 $$\begin{aligned} B_{T}(p,q)\le \frac{2}{\vert (p+q)^2-\mu \vert + \vert (p-q)^2-\mu \vert }. \end{aligned}$$

Then, we scale out $\mu$ and estimate the expression by pulling the supremum over $\psi$ into the ${\tilde{p}}$-integral

6.19 $$\begin{aligned}  &   \sup _{{\tilde{q}}\in {\mathbb {R}}^{d-1}} \sup _{\psi \in L^2({\mathbb {R}}), \Vert \psi \Vert =1} \int _{{\mathbb {R}}^{d+1}} \frac{2\chi _{\vert {\tilde{p}} \vert<2 \sqrt{\mu }}\overline{\psi (p_1)}\psi (q_1)}{\vert (p+q)^2-\mu \vert + \vert (p-q)^2 -\mu \vert } \ {\textrm{d}}p{\textrm{d}}q_1 \nonumber \\  &   \quad =\mu ^{d/2-1}\sup _{{\tilde{q}}\in {\mathbb {R}}^{d-1}} \sup _{\psi \in L^2({\mathbb {R}}), \Vert \psi \Vert =1} \int _{{\mathbb {R}}^{d+1}} \frac{2\chi _{\vert {\tilde{p}} \vert<2 }\overline{\psi (p_1)}\psi (q_1)}{\vert (p+q)^2-1\vert + \vert (p-q)^2 -1 \vert } \ {\textrm{d}}p {\textrm{d}}q_1 \nonumber \\  &   \quad \le \mu ^{d/2-1}\sup _{{\tilde{q}}\in {\mathbb {R}}^{d-1}} \int _{{\mathbb {R}}^{d-1}} \chi _{\vert {\tilde{p}} \vert <2 } \nonumber \\  &   \qquad \times \left[ \sup _{\psi \in L^2({\mathbb {R}}), \Vert \psi \Vert =1} \int _{{\mathbb {R}}^2}\frac{2\overline{\psi (p_1)}\psi (q_1)}{\vert (p+q)^2-1\vert + \vert (p-q)^2 -1 \vert } \ {\textrm{d}}p_1 {\textrm{d}}q_1\right] {\textrm{d}}{\tilde{p}}.\nonumber \\ \end{aligned}$$

Let $\mu _1=1-({\tilde{p}}+{\tilde{q}})^2$ and $\mu _2=1-({\tilde{p}}-{\tilde{q}})^2$. For fixed $\mu _1,\mu _2$ we need to bound the operator with integral kernel

6.20 $$\begin{aligned} D_{\mu _1,\mu _2}(p_1,q_1)=\frac{2}{\vert (p_1+q_1)^2-\mu _1\vert + \vert (p_1-q_1)^2 -\mu _2\vert }. \end{aligned}$$

#### Lemma 6.5

Let $\mu _1,\mu _2\le 1$ with $\min \{\mu _1,\mu _2\}\ne 0$. The operator $D_{\mu _1,\mu _2}$ on $L^2({\mathbb {R}})$ with integral kernel given by (6.20) satisfies

6.21 $$\begin{aligned} \Vert D_{\mu _1,\mu _2} \Vert \le C(1+d(\mu _1,\mu _2)\vert \min \{\mu _1,\mu _2\} \vert ^{-1/2}) \end{aligned}$$

for some finite *C* independent of $\mu _1,\mu _2$, where

6.22 $$\begin{aligned} d(\mu _1,\mu _2)=\left\{ \begin{matrix} 1+\ln \left( 1+\frac{\max \{\mu _1,\mu _2\} }{|\min \{\mu _1,\mu _2\}|}\right) &  \textrm{if}\quad \min \{\mu _1,\mu _2\}<0\le \max \{\mu _1,\mu _2\}, \\ 1 &  \mathrm{otherwise.} \end{matrix}\right. \nonumber \\ \end{aligned}$$

This is a generalization of [6, Lemma 4.2]. The proof of Lemma 6.5 is based on the Schur test and can be found in Sect. 7.1. Since $\max \{\mu _1,\mu _2\} \le 1$, it follows from Lemma 6.5 that for any $\alpha >1/2$ one has $\Vert D_{\mu _1,\mu _2} \Vert \le C\left( 1+ \vert \min \{\mu _1,\mu _2\} \vert ^{-\alpha } \right)$ for a constant *C* independent of $\mu _1,\mu _2$. The following Lemma concludes the proof of $\sup _{T>0} \Vert G_T^<\Vert <\infty$.$\square$

#### Lemma 6.6

Let $d\in \{2,3\}$ and $0\le \alpha <1$. Let $\mu _1=1-({\tilde{p}}+{\tilde{q}})^2$ and $\mu _2=1-({\tilde{p}}-{\tilde{q}})^2$. Then

6.23 $$\begin{aligned} \sup _{{\tilde{q}} \in {\mathbb {R}}^{d-1}} \int _{{\mathbb {R}}^{d-1}} \frac{ \chi _{\vert {\tilde{p}} \vert<2 }}{\vert \min \{\mu _1,\mu _2\}\vert ^{\alpha }}{\textrm{d}}{\tilde{p}}<\infty . \end{aligned}$$

Lemma 6.6 follows from elementary computations carried out in Sect. 7.2.

### Proof of Lemma 6.2

#### Proof of Lemma 6.2

With the notation introduced in the proof of Lemma 6.1 we have $\Vert {\mathbb {I}}_{\le \epsilon } G_T {\mathbb {I}}_{\le \epsilon } \Vert \le \Vert {\mathbb {I}}_{\le \epsilon } G_T^< {\mathbb {I}}_{\le \epsilon } \Vert +\Vert {\mathbb {I}}_{\le \epsilon } G_T^> {\mathbb {I}}_{\le \epsilon } \Vert$.

For $d=2,3$ we have, analogously to (6.15),

6.24 $$\begin{aligned} \begin{aligned} \Vert {\mathbb {I}}_{\le \epsilon } G_T^> {\mathbb {I}}_{\le \epsilon } \Vert \le \sup _{ \psi \in L^2({\mathbb {R}}^{2d}), \Vert \psi \Vert =1} \int _{{\mathbb {R}}^{2d}}&\chi _{|q|<\epsilon }\chi _{|(p_1,{\tilde{q}})|<\epsilon }B_{T}\left( p,q\right) \chi _{\vert {\tilde{p}}\vert >2 \sqrt{\mu }} \\  &\times \vert F_1 V^{1/2}\psi (p,q) \vert ^2{\text {d}}q {\text {d}}p. \end{aligned} \end{aligned}$$

Let $1<t<\infty$ such that $V\in L^t({\mathbb {R}}^d)$. According to Lemma 3.7(ii), for fixed *q* we have

6.25 $$\begin{aligned} \Vert F_1 V^{1/2}\psi (\cdot , q) \Vert _{L^s({\mathbb {R}}^d)}\le C \Vert V\Vert _t^{1/2} \Vert \psi (\cdot , q) \Vert _{L^2({\mathbb {R}}^d)}, \end{aligned}$$

where $2\le s=2t/(t-1)<\infty$. By (2.3) and Hölder’s inequality in *p*, there is a constant *C* independent of *T* such that

6.26 $$\begin{aligned} \Vert {\mathbb {I}}_{\le \epsilon } G_T^> {\mathbb {I}}_{\le \epsilon } \Vert\le &   C\sup _{ \psi \in L^2({\mathbb {R}}^{2d}), \Vert \psi \Vert =1} \int _{{\mathbb {R}}^{2d}} \frac{\chi _{|p_1|<\epsilon }}{1+{\tilde{p}}^2}\vert F_1 V^{1/2}\psi (p,q) \vert ^2 {\textrm{d}}p {\textrm{d}}q\nonumber \\\le &   C \Vert V\Vert _t \left( \int _{{\mathbb {R}}^d} \frac{\chi _{|p_1|<\epsilon }}{(1+{\tilde{p}}^2)^t} {\textrm{d}}p\right) ^{1/t}. \end{aligned}$$

In particular, the remaining integral is of order $O(\epsilon ^{1/t})$ and vanishes as $\epsilon \rightarrow 0$.

To estimate $\Vert {\mathbb {I}}_{\le \epsilon } G_T^< {\mathbb {I}}_{\le \epsilon } \Vert$ we proceed as in the derivation of the bound on $\Vert G_T^<\Vert$ from (6.16) until the first line of (6.19) and obtain

6.27 $$\begin{aligned} \begin{aligned} \Vert {\mathbb {I}}_{\le \epsilon }&G_T^< {\mathbb {I}}_{\le \epsilon } \Vert \le C \Vert V \Vert _1 \times \\  &\sup _{|{\tilde{q}}|<\epsilon } \sup _{\psi \in L^2({\mathbb {R}}), \Vert \psi \Vert =1} \int _{{\mathbb {R}}^{d+1}} \frac{2\chi _{|p_1|, |q_1|<\epsilon }\chi _{\vert {\tilde{p}} \vert <2 \sqrt{\mu }}\overline{\psi (p_1)}\psi (q_1)}{\vert (p+q)^2-\mu \vert + \vert (p-q)^2 -\mu \vert } \ {\text {d}}p{\text {d}}q_1. \end{aligned}\nonumber \\ \end{aligned}$$

Hence, we need that the norm of the operator on $L^2({\mathbb {R}})$ with integral kernel

6.28 $$\begin{aligned} \int _{{\mathbb {R}}^{d-1}} \frac{2\chi _{|p_1|, |q_1|<\epsilon }\chi _{\vert {\tilde{p}} \vert <2 \sqrt{\mu }}}{\vert (p+q)^2-\mu \vert + \vert (p-q)^2 -\mu \vert } {\textrm{d}}{\tilde{p}} \end{aligned}$$

vanishes uniformly in ${\tilde{q}}$ as $\epsilon \rightarrow 0$. In $d=1$, the Hilbert–Schmidt norm clearly vanishes as $\epsilon \rightarrow 0$. Similarly, for $d=2,3$, the following Lemma implies that the Hilbert–Schmidt norm vanishes uniformly in ${\tilde{q}}$ as $\epsilon \rightarrow 0$. $\square$

#### Lemma 6.7

Let $$d\in \{2,3\}$$. Then

6.29 $$\begin{aligned} \lim _{\epsilon \rightarrow 0} \sup _{|{\tilde{q}}|<\epsilon }\int _{{\mathbb {R}}^2} \chi _{|p_1|, |q_1|<\epsilon } \left[ \int _{{\mathbb {R}}^{d-1}} \frac{2\chi _{{\tilde{p}}^2<2}}{\vert (p+q)^2-1 \vert + \vert (p-q)^2-1 \vert } {\textrm{d}}{\tilde{p}} \right] ^2 {\textrm{d}}p_1 {\textrm{d}}q_1=0 \end{aligned}$$

The proof can be found in Sect. 7.3. We give the proof for $d=2$ only; the one for $d=3$ works analogously and is left to the reader.

### Proof of Lemma 6.3

#### Proof of Lemma 6.3

Since $a_T^{\Omega _0}$ diverges like $e_\mu \mu ^{d/2-1}\ln (\mu /T)$ as $T\rightarrow 0$, the claim follows if we prove that $\sup _{T>0} \sup _{|q|>\epsilon }\Vert V^{1/2} B_{T}(\cdot , q) V^{1/2}\Vert <\infty$. For $d=1$ we have

6.30 $$\begin{aligned} \begin{aligned} \Vert V^{1/2} B_{T}(\cdot , q) V^{1/2}\Vert ^2\le&\Vert V^{1/2} B_{T}(\cdot , q) V^{1/2} \Vert _\mathrm {{HS}}^2 \\=&\int _{{\mathbb {R}}^2} V(r) V(r') \left( \int _{\mathbb {R}}B_{T}(p,q) \frac{e^{i p(r- r')} }{2\pi }{\text {d}}p\right) ^2 {\text {d}}r {\text {d}}r' \\\le&\frac{1}{(2\pi )^2}\Vert V\Vert _1^2 \left( \int _{\mathbb {R}}B_{T}(p,q){\text {d}}p\right) ^2. \end{aligned}\nonumber \\ \end{aligned}$$

It was shown in the proof of [6, Lemma 4.4] that $\sup _{T>0,|q|>\epsilon } \int _{\mathbb {R}}B_{T}(p,q){\textrm{d}}p<\infty$.

For $d\in \{2,3\}$, the claim follows from the following Lemma which is proved below.

#### Lemma 6.8

Let $d\in \{2,3\}$ and $\mu >0$. Let *V* satisfy Assumption 1.1 and $V\ge 0$. Recall that $O_\mu {=}V^{1/2} {\mathcal {F}}^\dagger {\mathcal {F}}V^{1/2}$ (defined above (3.2)). Let $f(x){=}\chi _{(0,1/2)}(x)\ln (1/x)$. There is a constant $C(d,\mu ,V)$ such that for all $T>0$, $q\in {\mathbb {R}}^d$, and $\psi \in L^2({\mathbb {R}}^d)$ with $\Vert \psi \Vert _2=1$

6.31 $$\begin{aligned} \langle \psi , V^{1/2} B_{T}(\cdot , q) V^{1/2} \psi \rangle\le &   \mu ^{d/2-1} \langle \psi , O_\mu \psi \rangle f(\max \{T/\mu , \vert q \vert /\sqrt{\mu }\})\nonumber \\  &   + C(d,\mu ,V). \end{aligned}$$

This concludes the proof.$\square$

#### Proof of Lemma 6.8

**Fig. 3:**
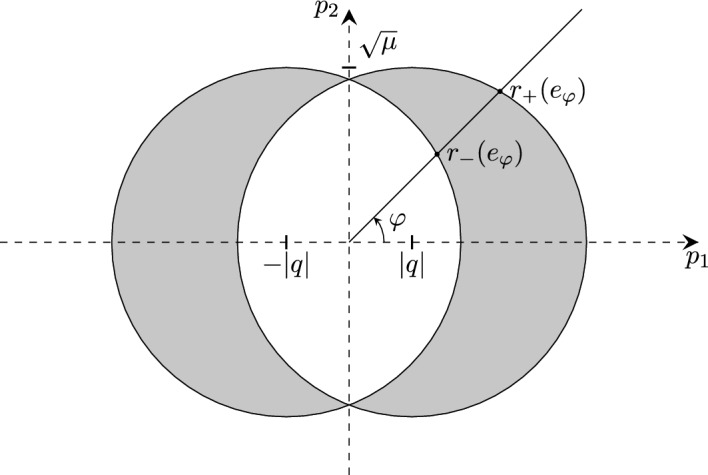
Two circles of radius $\sqrt{\mu }$, centered at $(-\vert q \vert ,0)$ and $(\vert q \vert ,0)$. In $d=2$ the function $B_T(p,(\vert q \vert ,0))$ diverges on the two circles as $T\rightarrow 0$ and approaches zero in the shaded area. Given an angle $\varphi$, the numbers $r_\pm (e_\varphi )$ are the distances between zero and the intersections of the circles with the ray tilted by an angle $\varphi$ with respect to the $p_1$-axis

Note that if we set $q=0$, and optimize over $\psi$, the left hand side would have the asymptotics $a_{T,\mu }^0\sim e_\mu \mu ^{d/2-1} \ln (1/T)$ as $T \rightarrow 0$. Intuitively, keeping *q* away from 0 on a scale larger than *T* will slow down the divergence. In the case $q=0$, divergence comes from the singularity on the set $\vert p \vert =\sqrt{\mu }$. For $\vert q\vert >0$, there will be two relevant sets, $(p+q)^2=\mu$ and $(p-q)^2=\mu$. These sets are circles or spheres in 2d and 3d, respectively. The function $B_{T}$ is very small on the region which lies inside exactly one of the disks or balls (see the shaded area in Fig. 3). The part lying inside or outside both disks (the white area in Fig. 3) will be relevant for the asymptotics. Define the family of operators $Q_{T}(q): L^1({\mathbb {R}}^d)\rightarrow L^\infty ({\mathbb {R}}^d)$ for $q\in {\mathbb {R}}^d$ through

6.32 $$\begin{aligned} \begin{aligned} \langle \psi , Q_{T}(q) \psi \rangle = \chi _{\max \left\{ \frac{T}{\mu },\frac{\vert q \vert }{\sqrt{\mu }}\right\}< \frac{1}{2}}&\int _{{\mathbb {R}}^d}\left| \widehat{\psi }\left( \sqrt{\mu }p/\vert p \vert \right) \right| ^2 B_{T}(p,q) \\  &\times \chi _{((p+q)^2-\mu )((p-q)^2-\mu )>0} \chi _{p^2 < 3\mu } {\text {d}}p. \end{aligned}\end{aligned}$$

We claim that $Q_{T}$ captures the divergence of $B_{T}$.

#### Lemma 6.9

Let $d\in \{2,3\}$ and $\mu >0$. Let *V* satisfy Assumption 1.1. Then

6.33 $$\begin{aligned} \sup _{T>0} \sup _{q \in {\mathbb {R}}^d} \Vert V^{1/2} B_{T}(\cdot , q) |V|^{1/2}- V^{1/2} Q_{T}(q) |V|^{1/2}\Vert <\infty . \end{aligned}$$

The proof of Lemma 6.9 can be found in Sect. 7.4. It now suffices to prove that there is a constant *C* such that for all $T> 0$ and $q\in {\mathbb {R}}^d$

6.34 $$\begin{aligned} \langle \psi , Q_{T}(q) \psi \rangle \le \mu ^{d/2-1}\langle \psi , \mathcal {F^\dagger }\mathcal {F} \psi \rangle f(\max \{T/\mu , \vert q \vert /\sqrt{\mu }\}) + C \Vert \psi \Vert _1^2.\nonumber \\ \end{aligned}$$

Then for all $\psi \in L^2({\mathbb {R}}^d)$ with $\Vert \psi \Vert _2=1$

6.35 $$\begin{aligned} \langle \psi , V^{1/2} Q_{T}(q) V^{1/2} \psi \rangle \le \mu ^{d/2-1}\langle \psi , O_\mu \psi \rangle f(\max \{T/\mu , \vert q \vert /\sqrt{\mu }\}) + C \Vert V \Vert _1,\nonumber \\ \end{aligned}$$

and the claim follows with Lemma 6.9.

We are left with proving (6.34). By the definition of $Q_T$, it suffices to restrict to $\vert q \vert<\sqrt{\mu }/2, T<\mu /2$. Let *R* be the rotation in ${\mathbb {R}}^d$ around the origin such that $q=R(\vert q \vert , {\tilde{0}})$. For $d=2$ the condition $((p+(|q|,0))^2-\mu )((p-(|q|,0))^2-\mu )>0$ holds exactly in the white region sketched in Fig. 3. The inner white region is characterized by $(\vert p_1\vert +\vert q\vert )^2+{\tilde{p}}^2<\mu$, and the outer region by $(\vert p_1\vert -\vert q\vert )^2+{\tilde{p}}^2>\mu$. Thus,

6.36 $$\begin{aligned} \begin{aligned} \langle \psi , Q_{T}(q) \psi \rangle = \int _{{\mathbb {R}}^d}&\left| \widehat{\psi }\left( \sqrt{\mu }R p/\vert p \vert \right) \right| ^2 \left[ \chi _{(\vert p_1\vert +\vert q\vert )^2+{\tilde{p}}^2<\mu }+\chi _{(\vert p_1\vert -\vert q\vert )^2+{\tilde{p}}^2>\mu }\right] \\  &\times B_{T}(p,(\vert q\vert ,{\tilde{0}}))\chi _{p^2 < 3\mu } {\text {d}}p, \end{aligned} \end{aligned}$$

where we substituted *p* by *Rp*.

Let us use the notation $r_{\pm }(e)=\pm \vert e_1 \vert \vert q \vert +\sqrt{\mu -e_2^2 \vert q\vert ^2}$ and $e_\varphi =(\cos \varphi ,\sin \varphi )$, where the choice of $r_\pm$ is motivated in Fig. 3. For $d=2$ rewriting the integral (6.36) in angular coordinates gives

6.37 $$\begin{aligned} \int _{0}^{2\pi }\!\! \left| \widehat{\psi }\left( \sqrt{\mu }R e_\varphi \vert \right) \right| ^2 \left[ \int _0^{r_-(e_\varphi )} B_{T}(r e_\varphi ,(\vert q\vert ,0)) r{\textrm{d}}r +\int _{r_+(e_\varphi )}^{\sqrt{3\mu }} B_{T}(r e_\varphi ,(\vert q\vert ,0)) r{\textrm{d}}r \right] {\textrm{d}}\varphi . \end{aligned}$$

For $d=3$ with the notation $e_{\varphi ,\theta } =(\cos \varphi , \sin \varphi \cos \theta , \sin \varphi \sin \theta )$ and using that $B_{T}(r e_{\varphi ,\theta },(\vert q\vert ,0,0))= B_{T}(r e_\varphi ,(\vert q\vert ,0))$, (6.36) equals

6.38 $$\begin{aligned} \begin{aligned} \int _{0}^{\pi }\left( \int _0^{2\pi } \left| \widehat{\psi }\left( \sqrt{\mu }r e_{\varphi ,\theta } \vert \right) \right| ^2 {\text {d}}\theta \right)&\left[ \int _0^{r_-(e_\varphi )} B_{T}(r e_\varphi ,(\vert q\vert ,0)) r^2{\text {d}}r \right. \\  &\left. +\int _{r_+(e_\varphi )}^{\sqrt{3\mu }} B_{T}(r e_\varphi ,(\vert q\vert ,0)) r^2{\text {d}}r \right] \sin \varphi {\text {d}}\varphi . \end{aligned} \end{aligned}$$

We distinguish two cases depending on whether *r* is within distance $T/\sqrt{\mu }$ to $r_\pm$ or not. Note that $r_{-}(e) \ge -\vert q\vert +\sqrt{\mu }\ge \frac{\sqrt{\mu }}{2} \ge \frac{T}{\sqrt{\mu }}$ and $r_+(e)+\frac{T}{\sqrt{\mu }}\le \vert q\vert +\sqrt{\mu }+T\le 2\sqrt{\mu }$. If *r* is close to $r_\pm$ we use that $B_{T}(p,q)\le 1/2T$. Otherwise we use (6.18). The expressions in the square brackets in (6.37) and (6.38) are thus bounded by

6.39 $$\begin{aligned}  &   \int _0^{r_-(e_\varphi )-\frac{T}{\sqrt{\mu }}} \frac{r^{d-1}}{\mu -r^2-q^2}{\textrm{d}}r + \int _{r_-(e_\varphi )-\frac{T}{\sqrt{\mu }}}^{r_-(e_\varphi )} \frac{r^{d-1}}{2T}{\textrm{d}}r \nonumber \\  &   \quad + \int _{r_+(e_\varphi )}^{r_+(e_\varphi )+\frac{T}{\sqrt{\mu }}} \frac{r^{d-1}}{2T}{\textrm{d}}r+\int _{r_+(e_\varphi )+\frac{T}{\sqrt{\mu }}}^{\sqrt{3\mu }} \frac{r^{d-1}}{r^2+q^2-\mu }{\textrm{d}}r. \end{aligned}$$

The second and third term are clearly bounded for $T<\mu /2$. Since $\Vert \widehat{\psi }\Vert _\infty \le (2\pi )^{-d/2} \Vert \psi \Vert _1$, they contribute $C \Vert \psi \Vert _1$ to the upper bound on $\langle \psi , Q_{T}(q) \psi \rangle$.

To bound the contributions of the first and the last term in (6.39) we treat $d=2$ and $d=3$ separately.

**Case** $d=2$: The sum of the two integrals equals

6.40 $$\begin{aligned} \ln \sqrt{\frac{(\mu -q^2)(2\mu +q^2)}{(\mu -q^2-(r_-(e_\varphi )-\frac{T}{\sqrt{\mu }})^2)((r_+(e_\varphi )+\frac{T}{\sqrt{\mu }})^2+q^2-\mu )}}. \end{aligned}$$

To bound this expression, we first make a few observations. Note that

6.41 $$\begin{aligned} \mu -q^2-\left( r_-(e_\varphi )-\frac{T}{\sqrt{\mu }}\right) ^2= &   2 \vert e_1 \vert \vert q \vert (\sqrt{\mu -e_2^2 \vert q \vert ^2}-\vert e_1\vert \vert q\vert )\nonumber \\  &   + \frac{T}{\sqrt{\mu }}\left( 2r_-(e_\varphi ) -\frac{T}{\sqrt{\mu }}\right) \nonumber \\\ge &   (\sqrt{3}-1) \sqrt{\mu }\vert e_1 \vert \vert q \vert + \frac{T}{2}, \end{aligned}$$

where we used that $r_-(e_\varphi )\ge \sqrt{\mu }-\vert q\vert$ and $\vert q\vert , T/\sqrt{\mu }\le \sqrt{\mu }/2$. Similarly,

6.42 $$\begin{aligned} \left( r_+(e_\varphi )+\frac{T}{\sqrt{\mu }}\right) ^2+q^2-\mu= &   2 \vert e_1 \vert \vert q \vert (\sqrt{\mu -e_2^2 \vert q \vert ^2}+\vert e_1\vert \vert q\vert )\nonumber \\  &   + \frac{T}{\sqrt{\mu }}\left( 2r_+(e_\varphi ) +\frac{T}{\sqrt{\mu }}\right) \nonumber \\\ge &   \sqrt{3} \sqrt{\mu } \vert e_1 \vert \vert q \vert + \sqrt{3} T. \end{aligned}$$

Furthermore, note that $2\mu +q^2 \le \frac{5\mu }{4}$. The expression under the square root in (6.40) is therefore bounded above by

6.43 $$\begin{aligned} \frac{ 5\mu ^2}{4( (\sqrt{3}-1) \sqrt{\mu }\vert e_1 \vert \vert q \vert + \frac{T}{2})(\sqrt{3} \sqrt{\mu } \vert e_1 \vert \vert q \vert + \sqrt{3} T)}. \end{aligned}$$

We now bound this from above in two ways. First we drop the *T* terms in the denominator, and second we drop the other terms in the denominator, which gives $\frac{ 5\mu }{4\sqrt{3} (\sqrt{3}-1) \vert e_1 \vert ^2 \vert q \vert ^2}$ and $\frac{5\mu ^2 }{2\sqrt{3}T^2}$, respectively. Thus, (6.40) is bounded above by $f(\max \{T/\mu , \vert q \vert /\sqrt{\mu }\}) + \ln (1/\vert e_1 \vert )+C$. The contribution to the upper bound on $\langle \psi , Q_{T}(q) \psi \rangle$ is

6.44 $$\begin{aligned}  &   \int _{0}^{2\pi } \left| \widehat{\psi }\left( \sqrt{\mu } e_\varphi \vert \right) \right| ^2 f(\max \{T/\mu , \vert q \vert /\sqrt{\mu }\}) {\textrm{d}}\varphi \nonumber \\  &   \quad +(2\pi )^{-2}\Vert \psi \Vert _1^2 \int _{0}^{2\pi }\left( \ln \left( 1/\vert \cos \varphi \vert \right) +C \right) {\textrm{d}}\varphi , \end{aligned}$$

where for the second term we used that $\vert \widehat{\psi }\left( \sqrt{\mu } e_\varphi \vert \right) \vert ^2 \le (2\pi )^{-2}\Vert \psi \Vert _1^2$. Note that the first summand equals $\langle \psi , \mathcal {F^\dagger }\mathcal {F} \psi \rangle f(\max \{T/\mu , \vert q \vert /\sqrt{\mu }\})$ and that the integral in the second summand is finite. In total, we have obtained (6.34) for $d=2$.

**Case** $d=3$: Note that $\frac{{\textrm{d}}}{{\textrm{d}}r} (-r + a {{\,\textrm{artanh}\,}}(r/a))= r^2/(a^2-r^2)$ and $\frac{{\textrm{d}}}{{\textrm{d}}r} (r - a {{\,\textrm{arcoth}\,}}(r/a))= r^2/(r^2-a^2)$. The sum of the first and the last integral in (6.39) hence equals

6.45 $$\begin{aligned}  &   \sqrt{3\mu }-r_+(e_\varphi )-r_-(e_\varphi )-\frac{\sqrt{\mu -q^2}}{2}\ln \left( \frac{(\sqrt{\mu -q^2}+\sqrt{3\mu })}{(\sqrt{3\mu }-\sqrt{\mu -q^2})}\right) \nonumber \\  &   \quad +\frac{\sqrt{\mu -q^2}}{2}\ln \left( \frac{(\sqrt{\mu -q^2}+r_-(e_\varphi )-\frac{T}{\sqrt{\mu }})}{(\sqrt{\mu -q^2}-r_-(e_\varphi )+\frac{T}{\sqrt{\mu }})}\frac{(\sqrt{\mu -q^2}+r_+(e_\varphi )+\frac{T}{\sqrt{\mu }})}{(r_+(e_\varphi )+\frac{T}{\sqrt{\mu }}-\sqrt{\mu -q^2})}\right) .\nonumber \\ \end{aligned}$$

The terms in the first line are bounded. The argument of the logarithm in the second line equals

6.46 $$\begin{aligned}  &   \frac{(\sqrt{\mu -q^2}+r_-(e_\varphi )-\frac{T}{\sqrt{\mu }})^2}{(\mu -q^2-(r_-(e_\varphi )-\frac{T}{\sqrt{\mu }})^2)}\frac{(\sqrt{\mu -q^2}+r_+(e_\varphi )+\frac{T}{\sqrt{\mu }})^2}{((r_+(e_\varphi )+\frac{T}{\sqrt{\mu }})^2-\mu +q^2)}\nonumber \\  &   \quad \le \frac{C\mu ^2}{( (\sqrt{3}-1) \sqrt{\mu }\vert e_1 \vert \vert q \vert + \frac{T}{2})(\sqrt{3} \sqrt{\mu } \vert e_1 \vert \vert q \vert + \sqrt{3} T))}, \end{aligned}$$

where we used (6.41) and (6.42). Analogously to the case $d=2$ the contribution to the upper bound on $\langle \psi , Q_{T}(q) \psi \rangle$ is

6.47 $$\begin{aligned}  &   \sqrt{\mu }\int _{0}^{\pi }\left( \int _0^{2\pi } \left| \widehat{\psi }\left( \sqrt{\mu } e_{\varphi ,\theta } \vert \right) \right| ^2{\textrm{d}}\theta \right) f(\max \{T/\mu , \vert q \vert /\sqrt{\mu }\})\sin \varphi {\textrm{d}}\varphi \nonumber \\  &   \quad +(2\pi )^{-2}\sqrt{\mu }\Vert \psi \Vert _1^2 \int _{0}^{\pi }\left( \ln \left( 1/\vert \cos \varphi \vert \right) +C \right) \sin \varphi {\textrm{d}}\varphi , \end{aligned}$$

and (6.34) follows.$$\square $$

## Proofs of Auxiliary Lemmas

### Proof of Lemma 6.5

#### Proof of Lemma 6.5

If we write $D_{\mu _1,\mu _2}$ as a sum $D_{\mu _1,\mu _2}=\sum _{j=1}^n D_{\mu _1,\mu _2}^j$ a.e. for some integral kernels $D_{\mu _1,\mu _2}^j$, then $\Vert D_{\mu _1,\mu _2} \Vert \le \sum _{j=1}^n \Vert D_{\mu _1,\mu _2}^j \Vert$. We will choose the $D_{\mu _1,\mu _2}^j$ as localized versions of $D_{\mu _1,\mu _2}$ in different regions (by multiplying $D_{\mu _1,\mu _2}$ by characteristic functions).

Let $D_{\mu _1,\mu _2}^1=D_{\mu _1,\mu _2}\chi _{\max \{\vert p_1 \vert , \vert q_1\vert \}>2}$ and $D_{\mu _1,\mu _2}^2=D_{\mu _1,\mu _2}\chi _{\max \{\vert p_1 \vert , \vert q_1\vert \}<2}$. We first prove that the Hilbert–Schmidt norm of $D_{\mu _1,\mu _2}^1$ is bounded uniformly in $\mu _1, \mu _2$. Note that if $\max \{\vert p_1 \vert , \vert q_1\vert \}>2$, we have $\max \{(p_1\pm q_1)^2\}=(\vert p_1 \vert +\vert q_1 \vert )^2>4$ and $\mu _1,\mu _2\le 1$. Hence,

7.1 $$\begin{aligned} D_{\mu _1,\mu _2}^1(p_1,q_1)\le \frac{2\chi _{\max \{\vert p_1 \vert , \vert q_1\vert \}>2}}{(\vert p_1 \vert +\vert q_1 \vert )^2-1}\le \frac{2\chi _{\max \{\vert p_1 \vert , \vert q_1\vert \}>2}}{p_1^2+q_1^2-1}. \end{aligned}$$

For the Hilbert–Schmidt norm we obtain that

7.2 $$\begin{aligned} \Vert D_{\mu _1,\mu _2}^1 \Vert _{ \mathrm HS}^2\le 4 \int _{{\mathbb {R}}^2} \frac{\chi _{\max \{\vert p_1 \vert , \vert q_1\vert \}>2}}{(p_1^2+q_1^2-1)^2} {\textrm{d}}p_1 {\textrm{d}}q_1\le 8 \pi \int _2^\infty \frac{r}{(r^2-1)^2} {\textrm{d}}r =\frac{4\pi }{3},\nonumber \\ \end{aligned}$$

and therefore $\Vert D_{\mu _1,\mu _2}^1 \Vert$ is indeed bounded uniformly in $\mu _1,\mu _2$.

For $D_{\mu _1,\mu _2}^2$ we first observe that $\Vert D_{\mu _2,\mu _1}^2 \Vert =\Vert D_{\mu _1,\mu _2}^2 \Vert$ since $D_{\mu _1,\mu _2}^2(p_1,q_1)=D_{\mu _2,\mu _1}^2(p_1,-q_1)$. Hence, without loss of generality we may assume $\mu _1\le \mu _2$ from now on. To bound the norm of $D_{\mu _1,\mu _2}^2$ we distinguish the cases $\mu _1<0$ and $\mu _1>0$ and continue localizing.

**Case** $\mu _1<0$: We localize in the regions $\vert p_1-q_1 \vert ^2<\mu _2$ and $\vert p_1-q_1 \vert ^2>\mu _2$, where the first one only occurs if $\mu _2>0$. Let $D_{\mu _1,\mu _2}^3=D_{\mu _1,\mu _2}^2 \chi _{\vert p_1-q_1 \vert ^2<\mu _2}$ and $D_{\mu _1,\mu _2}^4=D_{\mu _1,\mu _2}^2 \chi _{\vert p_1-q_1 \vert ^2>\mu _2}$.

For $D_{\mu _1,\mu _2}^3$ we do a Schur test with test function $h(p_1)=\vert p_1\vert ^{1/2}$. Using the symmetry of the integrand under $(p_1,q_1)\rightarrow -(p_1,q_1)$, we have

7.3 $$\begin{aligned} \Vert D_{\mu _1,\mu _2}^3 \Vert \le \sup _{-2<p_1<2} \vert p_1\vert ^{1/2} \int _{-2}^2 \frac{1}{2}\frac{\chi _{\vert p_1-q_1 \vert ^2<\mu _2}}{p_1q_1+(\mu _2-\mu _1)/4}\frac{1}{\vert q_1\vert ^{1/2}}{\textrm{d}}q_1\nonumber \\ = \chi _{0<\mu _2}\sup _{0\le p_1<2} \vert p_1\vert ^{1/2} \int _{p_1-\sqrt{\mu _2}}^{p_1+\sqrt{\mu _2}} \frac{1}{2} \frac{1}{p_1q_1+(\mu _2-\mu _1)/4}\frac{1}{\vert q_1\vert ^{1/2}}{\textrm{d}}q_1. \end{aligned}$$

For $\mu _2>0$, carrying out the integration we obtain

7.4 $$\begin{aligned} \Vert D_{\mu _1,\mu _2}^3 \Vert\le &   \sup _{0\le p_1<2} \frac{2}{\sqrt{\mu _2-\mu _1}}\left[ \arctan \left( \sqrt{\frac{4p_1 (p_1+\sqrt{\mu _2})}{\mu _2-\mu _1}}\right) \right. \nonumber \\  &   \left. -\chi _{p_1>\sqrt{\mu _2}}\arctan \left( \sqrt{\frac{4p_1 (p_1-\sqrt{\mu _2})}{\mu _2-\mu _1}}\right) \right. \nonumber \\  &   \left. +\chi _{p_1<\sqrt{\mu _2}}{{\,\textrm{artanh}\,}}\left( \sqrt{\frac{4p_1 (\sqrt{\mu _2}-p_1)}{\mu _2-\mu _1}}\right) \right] \nonumber \\  &   \le \frac{2}{\sqrt{\mu _2-\mu _1}}\left[ \frac{\pi }{2}+{{\,\textrm{artanh}\,}}\left( \sqrt{\frac{\mu _2}{\mu _2-\mu _1}}\right) \right] , \end{aligned}$$

where we used the monotonicity of ${{\,\textrm{artanh}\,}}$. Note that for, $x\ge 0$,

7.5 $$\begin{aligned} {{\,\textrm{artanh}\,}}\left( \sqrt{\frac{1}{1+x}}\right)= &   \ln \left( \sqrt{\frac{1}{x}+1}+\sqrt{\frac{1}{x}}\right) \le \ln \left( 2 \sqrt{\frac{1}{x}+1}\right) \nonumber \\= &   \ln (2)+\frac{1}{2}\ln \left( 1+\frac{1}{x}\right) . \end{aligned}$$

In total, we obtain

7.6 $$\begin{aligned} \Vert D_{\mu _1,\mu _2}^3\Vert \le \frac{C}{\sqrt{-\mu _1}}\left( 1+\ln \left( 1+\frac{\mu _2}{-\mu _1}\right) \right) \end{aligned}$$

for some constant *C*.

The Hilbert–Schmidt norm of $D_{\mu _1,\mu _2}^4$ is given by

7.7 $$\begin{aligned} \Vert D_{\mu _1,\mu _2}^4 \Vert _\textrm{HS}= \left( \int _{(-2,2)^2} \frac{\chi _{\vert p_1-q_1 \vert ^2>\mu _2}}{(p_1^2+q_1^2-\frac{\mu _1+\mu _2}{2})^2} {\textrm{d}}p_1 {\textrm{d}}q_1\right) ^{1/2}. \end{aligned}$$

For $\mu _2<0$, we clearly have $\Vert D_{\mu _1,\mu _2}^4 \Vert _\textrm{HS}\le \Vert D_{\mu _1,0}^4 \Vert _\textrm{HS}$. For $\mu _2\ge 0$ observe that the constraint $\vert p_1-q_1 \vert ^2>\mu _2$ implies $p_1^2+q_1^2 >\frac{\mu _2}{2}$. Hence,

7.8 $$\begin{aligned} \Vert D_{\mu _1,\mu _2}^4 \Vert _\textrm{HS}\le \left( 2\pi \int _{\sqrt{\frac{\mu _2}{2}}}^\infty \frac{r}{(r^2-\frac{\mu _1+\mu _2}{2})^2} {\textrm{d}}r\right) ^{1/2}= \left( \frac{2\pi }{-\mu _1}\right) ^{1/2}. \end{aligned}$$

**Fig. 4:**
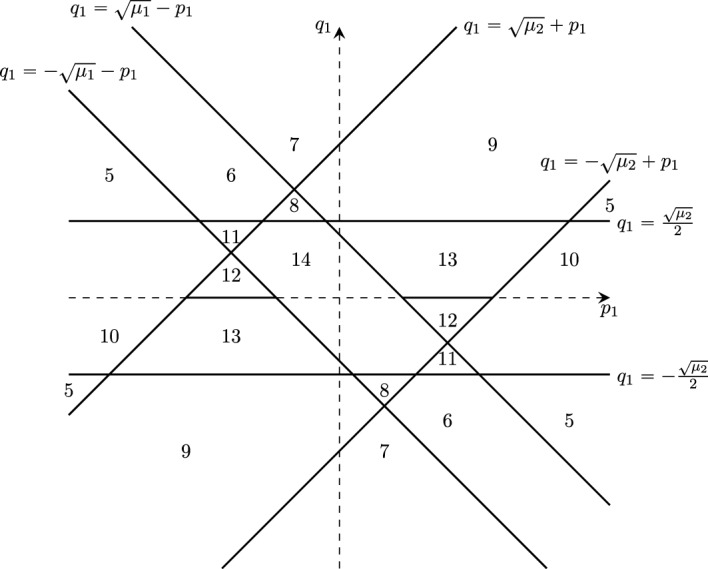
In the proof of Lemma 6.5, in the case $0<\mu _1\le \mu _2$ we split the domain of $p_1,q_1$ into ten different regions. The solid lines indicate the boundaries between these regions

**Case** $\mu _1>0$: We are left with estimating $D_{\mu _1,\mu _2}^2$ in the case that $\mu _1>0$. First we sketch the location of the singularities of $D_{\mu _1,\mu _2}^2(p_1,q_1)$. On each of the diagonal lines in Fig. 4, one of the two terms $|(p_1+q_1)^2-\mu _1|,|(p_1-q_1)^2-\mu _2|$ in the denominator of $D_{\mu _1,\mu _2}^2(p_1,q_1)$ vanishes. The function $D_{\mu _1,\mu _2}^2(p_1,q_1)$ thus has four singularities located at the crossings of the diagonal lines in Fig. 4. The coordinates of the singularities are $(p_1,q_1)\in \{(s_1,-s_2),(s_2,-s_1),(-s_1,s_2),(-s_2,s_1)\}$, where $s_1=\frac{\sqrt{\mu _1}+\sqrt{\mu _2}}{2}$, $s_2=\frac{\sqrt{\mu _2}-\sqrt{\mu _1}}{2}$. Note that $s_1^2+s_2^2=\frac{\mu _1+\mu _2}{2}$ and $s_1 s_2=\frac{\mu _2-\mu _1}{4}$.

To bound $\Vert D_{\mu _1,\mu _2}^2\Vert$, the idea is to perform a Schur test with test function $h(p_1)=\min \{\vert \vert p_1 \vert -s_1 \vert ^{1/2}, \vert \vert p_1 \vert -s_2 \vert ^{1/2}\}$. Since the behavior of $D_{\mu _1,\mu _2}^2(p_1,q_1)$ strongly depends on whether $|p_1+q_1|\gtrless \sqrt{\mu _1},|p_1-q_1|\gtrless \sqrt{\mu _2}$ and which singularity of $D_{\mu _1,\mu _2}^2$ is close to $p_1,q_1$, we distinguish the ten different regions sketched in Fig. 4. For $5\le j \le 14$, we define the operator $D_{\mu _1,\mu _2}^j$ to be localized in region *j*, $D_{\mu _1,\mu _2}^j=D_{\mu _1,\mu _2}^2 \chi _j$. According to the Schur test,

7.9 $$\begin{aligned} \Vert D_{\mu _1,\mu _2}^j\Vert \le \sup _{|p_1|<2}h(p_1)^{-1}\int _{-2}^2 D^j_{\mu _1,\mu _2}(p_1,q_1) h(q_1) {\textrm{d}}q_1. \end{aligned}$$

The bounds on $\Vert D_{\mu _1,\mu _2}^j\Vert$ we obtain from the Schur test are listed in Table 2. In the following we prove all the bounds:

**Table 2:** Overview of the estimates used in the proof of Lemma 6.5

Operator	Upper bound	Proof
$D^5$	$\frac{16}{\mu _1^{1/2}}$	(7.10)–(7.12)
$D^6$	$\frac{6}{\mu _1^{1/2}}$	(7.13)–(7.18)
$D^7$	$\frac{(6+2\sqrt{2})^{1/2}}{\mu _1^{1/2}}$	(7.19)–(7.21)
$D^8$	$\frac{2^{1/2} 4}{\mu _1^{1/2}}$	(7.22)–(7.26)
$D^9$	$\frac{8}{\mu _1^{1/2}}$	(7.27)–(7.29)
$D^{10}$	$\frac{4}{\mu _1^{1/2}}$	(7.30)–(7.33)
$D^{11}$	$\frac{4}{\mu _1^{1/2}}$	(7.34)–(7.37)
$D^{12}$	$\frac{2}{\mu _1^{1/2}}$	(7.38)–(7.40)
$D^{13}$	$\frac{4({{\,\textrm{artanh}\,}}(1/\sqrt{2})+\pi )}{\mu _1^{1/2}}$	(7.41)–(7.49)
$D^{14}$	$\frac{4(\sqrt{3}+1)}{\mu _1^{1/2}}$	(7.50)–(7.54)

**Region 5:** By the symmetry of the integrand under $(p_1,q_1)\rightarrow -(p_1,q_1)$, we have

7.10 $$\begin{aligned} \begin{aligned} \Vert D_{\mu _1,\mu _2}^{5} \Vert&\le \sup _{-2<p_1<2} h(p_1)\int _{-2}^2 \frac{\chi _{5}}{p_1^2+q_1^2-s_1^2-s_2^2} \frac{1}{h(q_1)} {\text {d}}q_1 \\  &= \sup _{\sqrt{\mu _1}+\frac{\sqrt{\mu _2}}{2}<p_1<2} \vert p_1-s_1 \vert ^{1/2}\Bigg (\int _{\sqrt{\mu _1}-p_1}^{-\frac{\sqrt{\mu _2}}{2}} \frac{1}{p_1^2+q_1^2-s_1^2-s_2^2} \frac{1}{\vert q_1+s_1 \vert ^{1/2}} {\text {d}}q_1 \\  &\qquad +\int _{\sqrt{\mu _2}/2}^{p_1-\sqrt{\mu _2}} \frac{1}{p_1^2+q_1^2-s_1^2-s_2^2} \frac{1}{\vert q_1-s_1 \vert ^{1/2}} {\text {d}}q_1 \Bigg )\\  &\le 2 \sup _{\sqrt{\mu _1}+\frac{\sqrt{\mu _2}}{2}<p_1<2}\Bigg [ \vert p_1-s_1 \vert ^{1/2} \\  &\qquad \times \int _{\sqrt{\mu _2}/2}^{p_1-\sqrt{\mu _1}} \frac{1}{p_1^2+q_1^2-s_1^2-s_2^2} \frac{1}{\vert q_1-s_1 \vert ^{1/2}} {\text {d}}q_1\Bigg ]\\  &\le 2\sup _{\sqrt{\mu _1}+\frac{\sqrt{\mu _2}}{2}<p_1<2} \frac{\vert p_1-s_1 \vert ^{1/2}}{p_1^2+\frac{\mu _2}{4}-s_1^2-s_2^2}\int _{\sqrt{\mu _2}/2}^{p_1-\sqrt{\mu _1}} \frac{1}{\vert q_1-s_1 \vert ^{1/2}} {\text {d}}q_1. \end{aligned}\end{aligned}$$

Note that $p_1^2+\frac{\mu _2}{4}-s_1^2-s_2^2=p_1^2-\frac{\mu _1}{2}-\frac{\mu _2}{4} \ge \frac{\sqrt{\mu _2}}{2} (p_1-\sqrt{\frac{\mu _1}{2}+\frac{\mu _2}{4}})$. Carrying out the integration, (7.10) is bounded above by

7.11 $$\begin{aligned}  &   \frac{8}{\sqrt{\mu _2}} \sup _{\sqrt{\mu _1}+\frac{\sqrt{\mu _2}}{2}<p_1<2} \frac{\vert p_1-s_1 \vert ^{1/2}}{p_1-\sqrt{\frac{\mu _1}{2}+\frac{\mu _2}{4}}} \nonumber \\  &   \quad \times \Bigg (\left( \frac{\sqrt{\mu _1}}{2}\right) ^{1/2}+\chi _{p_1>s_1+\sqrt{\mu _1}}\vert p_1-s_1-\sqrt{\mu _1} \vert ^{1/2}\Bigg ). \end{aligned}$$

Note that $s_1>\sqrt{\frac{\mu _1}{2}+\frac{\mu _2}{4}}$. Using that for $x\ge a\ge b$, $(x-a)/(x-b)\le 1$ we bound (7.11) above by

7.12 $$\begin{aligned}  &   \frac{8}{\sqrt{\mu _2}} \Bigg (\frac{ \left( \frac{\sqrt{\mu _1}}{2}\right) ^{1/2}}{|\sqrt{\mu _1}+\frac{\sqrt{\mu _2}}{2}-\sqrt{\frac{\mu _1}{2}+\frac{\mu _2}{4}}|^{1/2}} +1\Bigg ) \nonumber \\  &   \quad \le \frac{8}{\sqrt{\mu _2}}\left( \frac{ \sqrt{\mu _1}+\frac{\sqrt{\mu _2}}{2}+\sqrt{\frac{\mu _1}{2}+\frac{\mu _2}{4}}}{\sqrt{\mu _1}+2\sqrt{\mu _2}}\right) ^{1/2} +\frac{8}{\sqrt{\mu _1}} \le \frac{16}{\sqrt{\mu _1}}. \end{aligned}$$

**Region 6:** By symmetry under $(p_1,q_1)\rightarrow -(p_1,q_1)$, we obtain

7.13 $$\begin{aligned} \begin{aligned}&\Vert D_{\mu _1,\mu _2}^{6} \Vert \\  &\le \sup _{-2<p_1<2} h(p_1)\int _{-2}^2 \frac{1}{2}\frac{\chi _{6}}{-p_1q_1-s_1s_2} \frac{1}{h(q_1)} {\text {d}}q_1 \\  &\le \sup _{-2<p_1<-s_2} \Bigg [ h(p_1) \\  &\qquad \times \int _{\max \{-\sqrt{\mu _1}-p_1,\frac{\sqrt{\mu _2}}{2},\sqrt{\mu _2}+p_1\}}^{\min \{-p_1+\sqrt{\mu _1},2\}} \frac{1}{-p_1q_1-s_1s_2}\frac{1}{\vert q_1-s_1 \vert ^{1/2}} {\text {d}}q_1\Bigg ]. \end{aligned} \end{aligned}$$

We split the integral into the sum of the integral over $q_1>s_1$ and $q_1<s_1$. For $p_1<-s_2$ and $q_1>s_1$ we have $-p_1q_1-s_1s_2>-(p_1+s_2)s_1$. Hence,

7.14 $$\begin{aligned}  &   \sup _{-2<p_1<-s_2} h(p_1) \int _{s_1}^{\min \{-p_1+\sqrt{\mu _1},2\}} \frac{1}{-p_1q_1-s_1s_2} \frac{1}{\vert q_1-s_1 \vert ^{1/2}} {\textrm{d}}q_1\nonumber \\  &   \quad \le \sup _{-2<p_1<-s_2} \frac{1}{\vert p_1+s_2 \vert ^{1/2}s_1}\int _{s_1}^{-p_1+\sqrt{\mu _1}} \frac{1}{\vert q_1-s_1 \vert ^{1/2}} {\textrm{d}}q_1= \frac{2}{s_1} \le \frac{2}{\sqrt{\mu _1}}.\nonumber \\ \end{aligned}$$

The case $q_1<s_1$ only occurs for $p_1>-s_1-\sqrt{\mu _1}$. For $-\frac{\sqrt{\mu _2}}{2}<p_1<-s_2$ and $\sqrt{\mu _2}+p_1<q_1<s_1$ note that $-p_1q_1-s_1s_2\ge -p_1 (\sqrt{\mu _2}+p_1)-s_1s_2=\vert p_1+s_2\vert (p_1+s_1)\ge \vert p_1+s_2\vert \frac{\sqrt{\mu _1}}{2}$. Hence,

7.15 $$\begin{aligned}  &   \sup _{-\frac{\sqrt{\mu _2}}{2}<p_1<-s_2} h(p_1) \int _{\sqrt{\mu _2}+p_1}^{s_1} \frac{1}{-p_1q_1-s_1s_2} \frac{1}{\vert q_1-s_1 \vert ^{1/2}} {\textrm{d}}q_1\nonumber \\  &   \quad \le \sup _{-\frac{\sqrt{\mu _2}}{2}<p_1<-s_2}\frac{2}{\sqrt{\mu _1}\vert p_1+s_2\vert ^{1/2}} \int _{\sqrt{\mu _2}+p_1}^{s_1} \frac{1}{\vert q_1-s_1 \vert ^{1/2}} {\textrm{d}}q_1= \frac{4}{\sqrt{\mu _1}}.\nonumber \\ \end{aligned}$$

For $-s_1-\frac{\sqrt{\mu _1}}{2}<p_1< -\frac{\sqrt{\mu _2}}{2}$ and $\frac{\sqrt{\mu _2}}{2}<q_1<s_1$, we have $-p_1q_1-s_1s_2\ge \frac{\mu _2}{4}-s_1 s_2 =\frac{\mu _1}{4}$. Therefore,

7.16 $$\begin{aligned}  &   \sup _{-s_1-\frac{\sqrt{\mu _1}}{2}<p_1<-\frac{\sqrt{\mu _2}}{2}} h(p_1) \int _{\frac{\sqrt{\mu _2}}{2}}^{s_1} \frac{1}{-p_1q_1-s_1s_2} \frac{1}{\vert q_1-s_1 \vert ^{1/2}} {\textrm{d}}q_1\nonumber \\  &   \quad \le \sup _{-s_1-\frac{\sqrt{\mu _1}}{2}<p_1<-\frac{\sqrt{\mu _2}}{2}} \frac{4\vert p_1+s_1 \vert ^{1/2}}{\mu _1} \int _{\frac{\sqrt{\mu _2}}{2}}^{s_1} \frac{1}{\vert q_1-s_1 \vert ^{1/2}} {\textrm{d}}q_1\nonumber \\  &   \quad \le \frac{8 \left( \frac{\sqrt{\mu _1}}{2}\right) ^{1/2} }{\mu _1} \left( \frac{\sqrt{\mu _1}}{2}\right) ^{1/2}=\frac{4}{\mu _1^{1/2}}. \end{aligned}$$

For $-s_1-\sqrt{\mu _1}<p_1<-s_1-\frac{\sqrt{\mu _1}}{2}$ and $-p_1-\sqrt{\mu _1}<q_1<s_1$, we have $-p_1q_1-s_1s_2\ge p_1(p_1+\sqrt{\mu _1})-s_1 s_2 =-(p_1+s_1)(s_2-p_1)$. Hence,

7.17 $$\begin{aligned}  &   \sup _{-s_1-\sqrt{\mu _1}<p_1<-s_1-\frac{\sqrt{\mu _1}}{2}} h(p_1) \int _{-p_1-\sqrt{\mu _1}}^{s_1} \frac{1}{-p_1q_1-s_1s_2} \frac{1}{\vert q_1-s_1 \vert ^{1/2}} {\textrm{d}}q_1\nonumber \\  &   \quad \le \sup _{-s_1-\sqrt{\mu _1}<p_1<-s_1-\frac{\sqrt{\mu _1}}{2}} \frac{2\vert p_1+\sqrt{\mu _1}+s_1 \vert ^{1/2}}{\vert p_1+s_1 \vert ^{1/2}(s_2-p_1)} = \frac{2}{s_2+s_1+\frac{\sqrt{\mu _1}}{2}}\le \frac{4}{\sqrt{\mu _1}}.\nonumber \\ \end{aligned}$$

In total, summing the contributions from $q_1>s_1$ and $q_1<s_1$ gives

7.18 $$\begin{aligned} \Vert D_{\mu _1,\mu _2}^{6} \Vert \le \frac{6}{\sqrt{\mu _1}}. \end{aligned}$$

**Region 7:** By symmetry of the two components of region 7 we have

7.19 $$\begin{aligned} \begin{aligned} \Vert D_{\mu _1,\mu _2}^7 \Vert&\le \sup _{-2<p_1<2} h(p_1)\int _{-2}^2 \frac{\chi _7}{p_1^2+q_1^2-s_1^2-s_2^2} \frac{1}{h(q_1)} {\text {d}}q_1 \\  &\le 2 \sup _{-2<p_1<2}\Bigg [ \vert \vert p_1\vert -s_2 \vert ^{1/2} \\  &\quad \times \int _{\max \{\sqrt{\mu _1}-p_1, \sqrt{\mu _2}+p_1\}}^2 \frac{1}{p_1^2+q_1^2-s_1^2-s_2^2}\frac{1}{\vert q_1-s_1 \vert ^{1/2}} {\text {d}}q_1 \Bigg ]\end{aligned} \end{aligned}$$

For $\vert p_1 \vert >s_2$, $q_1>s_1$ we observe $p_1^2+q_1^2-s_1^2-s_2^2 \ge (q_1+s_1)(q_1-s_1) \ge 2s_1 (q_1-s_1)$. Therefore,

7.20 $$\begin{aligned}  &   \sup _{s_2<\vert p_1 \vert<2} \vert \vert p_1\vert -s_2 \vert ^{1/2} \int _{\max \{\sqrt{\mu _1}-p_1, \sqrt{\mu _2}+p_1\}}^2 \frac{1}{p_1^2+q_1^2-s_1^2-s_2^2} \frac{1}{\vert q_1-s_1 \vert ^{1/2}} {\textrm{d}}q_1\nonumber \\  &   \quad \le \sup _{s_2<\vert p_1 \vert<2} \frac{\vert \vert p_1\vert -s_2 \vert ^{1/2}}{2s_1} \int _{\max \{\sqrt{\mu _1}-p_1, \sqrt{\mu _2}+p_1\}}^\infty \frac{1}{(q_1-s_1)^{3/2}} {\textrm{d}}q_1\nonumber \\  &   \quad =\sup _{s_2<\vert p_1 \vert <2} \frac{\vert \vert p_1\vert -s_2 \vert ^{1/2}}{s_1 (\max \{\sqrt{\mu _1}-p_1, \sqrt{\mu _2}+p_1\}-s_1)^{1/2}}=\frac{1}{s_1}\le \frac{1}{\sqrt{\mu _1}}. \end{aligned}$$

For $\vert p_1 \vert <s_2$, $q_1>s_1$ we have $(p_1^2+q_1^2-s_1^2-s_2^2)(q_1-s_1)^{1/2} \ge (q_1+\sqrt{s_1^2+s_2^2-p_1^2})(q_1-\sqrt{s_1^2+s_2^2-p_1^2})^{3/2} \ge 2s_1 (q_1-\sqrt{s_1^2+s_2^2-p_1^2})^{3/2}$. Hence,

7.21 $$\begin{aligned} \sup _{\vert p_1 \vert<s_2}&\vert \vert p_1\vert -s_2 \vert ^{1/2} \int _{\max \{\sqrt{\mu _1}-p_1, \sqrt{\mu _2}+p_1\}}^2 \frac{1}{p_1^2+q_1^2-s_1^2-s_2^2} \frac{1}{\vert q_1-s_1 \vert ^{1/2}} {\textrm{d}}q_1\nonumber \\&\le \sup _{\vert p_1 \vert<s_2} \frac{\vert p_1+s_2 \vert ^{1/2}}{2s_1} \int _{\sqrt{\mu _2}+p_1}^\infty \frac{1}{(q_1-\sqrt{s_1^2+s_2^2-p_1^2})^{3/2} }{\textrm{d}}q_1\nonumber \\&= \sup _{\vert p_1 \vert<s_2} \frac{\vert p_1+s_2 \vert ^{1/2}}{s_1} \frac{1}{(\sqrt{\mu _2}+p_1-\sqrt{s_1^2+s_2^2-p_1^2})^{1/2} }\nonumber \\&=\sup _{\vert p_1 \vert<s_2} \frac{1}{s_1} \frac{\vert p_1+s_2 \vert ^{1/2}(\sqrt{\mu _2}+p_1+\sqrt{s_1^2+s_2^2-p_1^2})^{1/2} }{(p_1+s_1)^{1/2}(p_1+s_2)^{1/2}}\nonumber \\&=\sup _{\vert p_1 \vert <s_2} \frac{1}{s_1} \frac{(\sqrt{\mu _2}+p_1+\sqrt{s_1^2+s_2^2-p_1^2})^{1/2} }{(p_1+s_1)^{1/2}}\nonumber \\&\le \frac{(\frac{3}{2}+\sqrt{2})^{1/2} s_1^{1/2}}{s_1 \mu _1^{1/4}}\le \frac{(\frac{3}{2}+\sqrt{2})^{1/2}}{\mu _1^{1/2}}. \end{aligned}$$

In total, we obtain $\Vert D^7 \Vert \le \frac{(6+2\sqrt{2})^{1/2}}{\sqrt{\mu _1}}$.

**Region 8:** Taking the supremum separately over the two symmetric components of region 8, we have

7.22 $$\begin{aligned} \begin{aligned}&\Vert D_{\mu _1,\mu _2}^{8} \Vert \\  &\le \sup _{-2<p_1<2} h(p_1)\int _{-2}^2 \frac{\chi _{8}}{s_1^2+s_2^2-p_1^2-q_1^2} \frac{1}{h(q_1)} {\text {d}}q_1\\  &\le 2 \sup _{- \frac{\sqrt{\mu _2}}{2}<p_1<\sqrt{\mu _1}-\frac{\sqrt{\mu _2}}{2}}\Bigg [ h(p_1) \\  &\qquad \times \int _{\sqrt{\mu _2}/2}^{\min \{\sqrt{\mu _2}+p_1,\sqrt{\mu _1}-p_1\}} \frac{1}{s_1^2+s_2^2-p_1^2-q_1^2}\frac{1}{\vert s_1-q_1 \vert ^{1/2}} {\text {d}}q_1\Bigg ]\\  &\le 2\sup _{- \frac{\sqrt{\mu _2}}{2}<p_1<\sqrt{\mu _1}-\frac{\sqrt{\mu _2}}{2}}\Bigg [\frac{ h(p_1)}{\sqrt{\mu _2}} \\  &\qquad \times \int _{\sqrt{\mu _2}/2}^{\min \{\sqrt{\mu _2}+p_1,\sqrt{\mu _1}-p_1\}} \frac{1}{\sqrt{s_1^2+s_2^2-p_1^2}-q_1} \frac{1}{\vert s_1-q_1 \vert ^{1/2}} {\text {d}}q_1\Bigg ], \end{aligned} \end{aligned}$$

since $\sqrt{s_1^2+s_2^2-p_1^2}+q_1> \sqrt{\frac{\mu _1}{2}+\frac{\mu _2}{2}-\frac{\mu _2}{4}}+\frac{\sqrt{\mu _2}}{2} \ge \sqrt{\mu _2}$. For $\vert p_1 \vert >s_2$, we have $s_1>\sqrt{s_1^2+s_2^2-p_1^2}$, whereas for $\vert p_1 \vert <s_2$, $s_1<\sqrt{s_1^2+s_2^2-p_1^2}$. For $p_1<-s_2$ we obtain

7.23 $$\begin{aligned}&\sup _{- \frac{\sqrt{\mu _2}}{2}<p_1<-s_2}\frac{2 h(p_1)}{\sqrt{\mu _2}} \int _{\sqrt{\mu _2}/2}^{\min \{\sqrt{\mu _2}+p_1,\sqrt{\mu _1}-p_1\}} \frac{1}{\sqrt{s_1^2+s_2^2-p_1^2}-q_1} \frac{1}{\vert s_1-q_1 \vert ^{1/2}} {\textrm{d}}q_1\nonumber \\&\quad \le \sup _{- \frac{\sqrt{\mu _2}}{2}<p_1<-s_2}\frac{2 \vert p_1+s_2 \vert ^{1/2}}{\sqrt{\mu _2}} \int _{-\infty }^{\sqrt{\mu _2}+p_1} \frac{1}{(\sqrt{s_1^2+s_2^2-p_1^2}-q_1)^{3/2} }{\textrm{d}}q_1\nonumber \\&\quad =\sup _{- \frac{\sqrt{\mu _2}}{2}<p_1<-s_2}\frac{4 \vert p_1+s_2 \vert ^{1/2}}{\sqrt{\mu _2}(\sqrt{s_1^2+s_2^2-p_1^2}-\sqrt{\mu _2}-p_1)^{1/2}}\nonumber \\&\quad \le \sup _{- \frac{\sqrt{\mu _2}}{2}<p_1<-s_2}\frac{4 (\sqrt{s_1^2+s_2^2-p_1^2}+\sqrt{\mu _2}+p_1)^{1/2}}{2^{1/2}\sqrt{\mu _2}(p_1+s_1)^{1/2}} \le \frac{2^{1/2} 4 s_1^{1/2}}{\sqrt{\mu _2}\mu _1^{1/4}}\le \frac{2^{1/2} 4}{\mu _1^{1/2}}. \end{aligned}$$

Similarly, for $p_1>s_2$ (which only occurs if $2\sqrt{\mu _2}<3\sqrt{\mu _1}$),

7.24 $$\begin{aligned}&\sup _{s_2<p_1<\sqrt{\mu _1}-\frac{\sqrt{\mu _2}}{2}}\frac{2 h(p_1)}{\sqrt{\mu _2}} \int _{\sqrt{\mu _2}/2}^{\min \{\sqrt{\mu _2}+p_1,\sqrt{\mu _1}-p_1\}} \frac{1}{\sqrt{s_1^2+s_2^2-p_1^2}-q_1} \frac{1}{\vert s_1-q_1 \vert ^{1/2}} {\textrm{d}}q_1\nonumber \\&\quad \le \sup _{s_2<p_1<\frac{\sqrt{\mu _2}}{2}}\frac{2 \vert p_1-s_2 \vert ^{1/2}}{\sqrt{\mu _2}} \int _{-\infty }^{\sqrt{\mu _2}-p_1} \frac{1}{(\sqrt{s_1^2+s_2^2-p_1^2}-q_1)^{3/2} }{\textrm{d}}q_1 \le \frac{2^{1/2} 4}{\mu _1^{1/2}}, \end{aligned}$$

by (7.23). For $$\vert p_1 \vert <s_2$$,

7.25 $$\begin{aligned}  &   \sup _{- s_2<p_1<s_2}\frac{2 h(p_1)}{\sqrt{\mu _2}} \int _{\sqrt{\mu _2}/2}^{\sqrt{\mu _1}-p_1} \frac{1}{\sqrt{s_1^2+s_2^2-p_1^2}-q_1} \frac{1}{\vert s_1-q_1 \vert ^{1/2}}{\textrm{d}}q_1\nonumber \\  &   \quad \le \sup _{- s_2<p_1<s_2}\frac{2 \vert \vert p_1\vert -s_2 \vert ^{1/2}}{\sqrt{\mu _2}} \int _{-\infty }^{\sqrt{\mu _1}-p_1} \frac{1}{\vert s_1-q_1 \vert ^{3/2}}{\textrm{d}}q_1\nonumber \\  &   \quad = \sup _{- s_2<p_1<s_2}\frac{4 \vert \vert p_1\vert -s_2 \vert ^{1/2}}{\sqrt{\mu _2}\vert s_2+p_1 \vert ^{1/2}}=\frac{4}{\sqrt{\mu _2}}. \end{aligned}$$

In total, we have

7.26 $$\begin{aligned} \Vert D_{\mu _1,\mu _2}^{8} \Vert \le \frac{2^{1/2} 4}{\mu _1^{1/2}}. \end{aligned}$$

**Region 9:** By taking the supremum separately over the two components of region 9 and using the symmetry in $(p_1,q_1)\rightarrow -(p_1,q_1)$, we obtain

7.27 $$\begin{aligned} \begin{aligned} \Vert D_{\mu _1,\mu _2}^{9} \Vert \le&\sup _{-2<p_1<2} h(p_1)\int _{-2}^2 \frac{1}{2}\frac{\chi _{9}}{p_1q_1+s_1s_2} \frac{1}{h(q_1)} {\text {d}}q_1 \\\le&\sup _{-s_2<p_1<2} \Bigg [h(p_1) \\  &\quad \times \int _{\max \{\sqrt{\mu _1}-p_1,\sqrt{\mu _2}/2,p_1-\sqrt{\mu _2}\}}^{\min \{p_1+\sqrt{\mu _2},2\}} \frac{1}{p_1q_1+s_1s_2}\frac{1}{\vert q_1-s_1 \vert ^{1/2}} {\text {d}}q_1\Bigg ]. \end{aligned} \end{aligned}$$

For $p_1>-s_2$ and $\max \{\sqrt{\mu _1}-p_1, \frac{\sqrt{\mu _2}}{2}\}<q_1<\sqrt{\mu _2}+p_1$ note that

7.28 $$\begin{aligned} p_1 q_1+s_1 s_2&\ge \left\{ \begin{matrix} p_1(\sqrt{\mu _2}+p_1)+s_1 s_2 = (p_1+s_2)(p_1+s_1) &  \textrm{if }\ p_1\le 0\\ p_1(\sqrt{\mu _1}-p_1)+s_1 s_2 = (p_1+s_2)(s_1-p_1) &  \textrm{if }\ \sqrt{\mu _1}-\frac{\sqrt{\mu _2}}{2}\ge p_1\ge 0\\ p_1\frac{\sqrt{\mu _2}}{2}+s_1s_2 &  \textrm{if }\ p_1\ge \max \{\sqrt{\mu _1}-\frac{\sqrt{\mu _2}}{2},0\}\end{matrix} \right\} \nonumber \\&\ge \frac{\sqrt{\mu _1}}{2}(p_1+s_2). \end{aligned}$$

Hence,

7.29 $$\begin{aligned} \Vert D_{\mu _1,\mu _2}^{9} \Vert \le \sup _{-s_2<p_1<2} \frac{2}{\sqrt{\mu _1}(p_1+s_2)^{1/2}} \int _{\sqrt{\mu _1}-p_1}^{p_1+\sqrt{\mu _2}} \frac{1}{\vert q_1-s_1 \vert ^{1/2}} {\textrm{d}}q_1=\frac{8}{\sqrt{\mu _1}}.\nonumber \\ \end{aligned}$$

**Region 10:** By symmetry in $p_1$, we have

7.30 $$\begin{aligned}&\Vert D_{\mu _1,\mu _2}^{10} \Vert \le \sup _{-2<p_1<2} h(p_1)\int _{-2}^2 \frac{\chi _{10}}{p_1^2+q_1^2-s_1^2-s_2^2} \frac{1}{h(q_1)} {\textrm{d}}q_1\nonumber \\&\quad = \sup _{s_1<p_1<2} \vert p_1-s_1 \vert ^{1/2} \int _{\max \{\sqrt{\mu _1}-p_1,-\frac{\sqrt{\mu _2}}{2}\}}^{\min \{p_1-\sqrt{\mu _2},\frac{\sqrt{\mu _2}}{2}\}} \frac{1}{p_1^2+q_1^2-s_1^2-s_2^2} \frac{1}{\vert \vert q_1\vert -s_2 \vert ^{1/2}} {\textrm{d}}q_1. \end{aligned}$$

If we mirror the part of region 10 with $p_1>0,q_1<0$ along $q_1=0$, its image contains the part of region 10 with $p_1>0,q_1>0$. Since the integrand is symmetric in $q_1$, we can thus bound

7.31 $$\begin{aligned} \begin{aligned} \Vert D_{\mu _1,\mu _2}^{10} \Vert \le&\sup _{s_1<p_1<2} \Bigg [ 2\vert p_1-s_1 \vert ^{1/2} \\  &\times \int _{\max \{\sqrt{\mu _2}-p_1,0\}}^{\min \{p_1-\sqrt{\mu _1},\frac{\sqrt{\mu _2}}{2}\}}\frac{1}{p_1^2+q_1^2-s_1^2-s_2^2} \frac{1}{\vert q_1-s_2 \vert ^{1/2}} {\text {d}}q_1\Bigg ]. \end{aligned} \end{aligned}$$

Note that for $q_1\ge \sqrt{\mu _2}-p_1$, $p_1>s_1$ we have

7.32 $$\begin{aligned} p_1^2+q_1^2-s_1^2-s_2^2= &   (p_1-s_1)^2+(q_1-s_2)^2+2s_1 (p_1-s_1)+2s_2(q_1-s_2) \nonumber \\\ge &   2s_1 (p_1-s_1)+2s_2(s_1-p_1) = 2\sqrt{\mu _1}(p_1-s_1). \end{aligned}$$

Therefore,

7.33 $$\begin{aligned} \Vert D_{\mu _1,\mu _2}^{10} \Vert \le \sup _{s_1<p_1<2} \frac{1}{\sqrt{\mu _1}\vert p_1-s_1 \vert ^{1/2}} \int _{\sqrt{\mu _2}-p_1}^{p_1-\sqrt{\mu _1}} \frac{1}{\vert q_1-s_2 \vert ^{1/2}} {\textrm{d}}q_1=\frac{4}{\sqrt{\mu _1}}.\nonumber \\ \end{aligned}$$

**Region 11:** By symmetry in $p_1$, we obtain

7.34 $$\begin{aligned} \begin{aligned} \Vert D_{\mu _1,\mu _2}^{11} \Vert \le&\sup _{-2<p_1<2} h(p_1)\int _{-2}^2 \frac{1}{2}\frac{\chi _{11}}{-p_1q_1-s_1s_2} \frac{1}{h(q_1)} {\text {d}}q_1 \\=&\sup _{-\mu _1-\frac{\sqrt{\mu _2}}{2}<p_1<-\frac{\sqrt{\mu _2}}{2}}\Bigg [\frac{1}{2} \vert p_1+s_1 \vert ^{1/2} \\  &\times \int _{\max \{-\sqrt{\mu _1}-p_1,\sqrt{\mu _2}+p_1\}}^{\frac{\sqrt{\mu _2}}{2}} \frac{1}{-p_1q_1-s_1s_2} \frac{1}{\vert q_1-s_2 \vert ^{1/2}} {\text {d}}q_1\Bigg ]. \\ \end{aligned}\end{aligned}$$

For $p_1<-s_1$ we have $-p_1q_1-s_1s_2>s_1(q_1-s_2)$. Hence,

7.35 $$\begin{aligned}  &   \sup _{-\mu _1-\frac{\sqrt{\mu _2}}{2}<p_1<-s_1} \frac{1}{2}\vert p_1+s_1 \vert ^{1/2} \int _{-\sqrt{\mu _1}-p_1}^{\frac{\sqrt{\mu _2}}{2}} \frac{1}{-p_1q_1-s_1s_2}\frac{1}{\vert q_1-s_2 \vert ^{1/2}} {\textrm{d}}q_1\nonumber \\  &   \quad \le \sup _{-\mu _1-\frac{\sqrt{\mu _2}}{2}<p_1<-s_1} \frac{\vert p_1+s_1 \vert ^{1/2}}{2s_1} \int _{-\sqrt{\mu _1}-p_1}^{\infty } \frac{1}{\vert q_1-s_2 \vert ^{3/2}} {\textrm{d}}q_1 =\frac{1}{s_1} \le \frac{1}{\sqrt{\mu _1}}.\nonumber \\ \end{aligned}$$

For $p_1>-s_1$, we carry out the integration

7.36 $$\begin{aligned}  &   \sup _{-s_1<p_1<-\frac{\sqrt{\mu _2}}{2}} \frac{1}{2}\vert p_1+s_1 \vert ^{1/2} \int _{\sqrt{\mu _2}+p_1}^{\frac{\sqrt{\mu _2}}{2}} \frac{1}{-p_1q_1-s_1s_2}\frac{1}{\vert q_1-s_2 \vert ^{1/2}} {\textrm{d}}q_1\nonumber \\  &   \quad \le \sup _{-s_1<p_1<-\frac{\sqrt{\mu _2}}{2}} \frac{1}{\vert p_1 \vert ^{1/2} s_2^{1/2}} {{\,\textrm{artanh}\,}}\left( \frac{ s_2^{1/2}}{\vert p_1 \vert ^{1/2}}\right) =\frac{2^{1/2}}{\mu _2^{1/4}s_2^{1/2}} {{\,\textrm{artanh}\,}}\left( \frac{2^{1/2} s_2^{1/2}}{\mu _2^{1/4}}\right) .\nonumber \\ \end{aligned}$$

With ${{\,\textrm{artanh}\,}}(x)\le \frac{x}{1-x}$, we obtain

7.37 $$\begin{aligned} \frac{2^{1/2}}{\mu _2^{1/4}s_2^{1/2}} {{\,\textrm{artanh}\,}}\left( \frac{2^{1/2} s_2^{1/2}}{\mu _2^{1/4}}\right) \le \frac{2^{1/2}}{\mu _2^{1/4}s_2^{1/2}} \frac{s_2^{1/2}}{\frac{\mu _2^{1/4}}{2^{1/2}}-s_2^{1/2}} =\frac{2^{1/2}}{\mu _2^{1/4}} \frac{\frac{\mu _2^{1/4}}{2^{1/2}}+s_2^{1/2}}{\frac{\mu _1^{1/2}}{2}}\le \frac{4}{\mu _1^{1/2}}. \end{aligned}$$

Therefore, $$\Vert D_{\mu _1,\mu _2}^{11} \Vert \le \frac{4}{\mu _1^{1/2}}$$.

**Region 12:** By symmetry in $p_1$, we obtain

7.38 $$\begin{aligned}&\Vert D_{\mu _1,\mu _2}^{12} \Vert \le \sup _{-2<p_1<2} h(p_1)\int _{-2}^2 \frac{1}{2}\frac{\chi _{12}}{p_1q_1+s_1s_2} \frac{1}{h(q_1)} {\textrm{d}}q_1\nonumber \\&\quad = \sup _{-\sqrt{\mu _2}<p_1<-\sqrt{\mu _1}} \frac{1}{2} h(p_1) \int _{0}^{\min \{p_1+\sqrt{\mu _2},-\sqrt{\mu _1}-p_1\}} \frac{1}{p_1q_1+s_1s_2} \frac{1}{\vert s_2-q_1 \vert ^{1/2}} {\textrm{d}}q_1. \end{aligned}$$

For $p_1\ge -s_1$ note that $p_1 q_1+s_1 s_2 \ge s_1(s_2-q_1) \ge \frac{\sqrt{\mu _1}}{2}( s_2-q_2)$. For $p_1\le -s_1$ and $q_1<p_1+\sqrt{\mu _2}$ observe that

7.39 $$\begin{aligned} p_1 q_1+s_1 s_2&= (-p_1-s_1)(s_2-q_1)+s_1(s_2-q_1)+s_2(p_1+s_1) \nonumber \\&\ge \frac{\sqrt{\mu _1}}{2}(s_2-q_1)+\frac{\sqrt{\mu _2}}{2}(s_2-q_1)+s_2(q_1-\sqrt{\mu _2}+s_1)\nonumber \\&=\frac{\sqrt{\mu _1}}{2}(s_2-q_1)+\frac{\sqrt{\mu _2}}{2}(s_2-q_1)-s_2(s_2-q_1)\nonumber \\&\ge \frac{\sqrt{\mu _1}}{2}(s_2-q_1). \end{aligned}$$

Therefore,

7.40 $$\begin{aligned} \Vert D_{\mu _1,\mu _2}^{12} \Vert\le &   \sup _{-\sqrt{\mu _2}<p_1<-\sqrt{\mu _1}} \frac{\vert p_1+s_1 \vert ^{1/2}}{\sqrt{\mu _1}}\nonumber \\  &   \times \int _{-\infty }^{\min \{p_1+\sqrt{\mu _2},-\sqrt{\mu _1}-p_1\}} \frac{1}{\vert s_2-q_1 \vert ^{3/2}} {\textrm{d}}q_1 =\frac{2}{\sqrt{\mu _1}}. \end{aligned}$$

**Region 13:** By symmetry under $(p_1,q_1)\rightarrow -(p_1,q_1)$, we obtain

7.41 $$\begin{aligned} \begin{aligned} \Vert D_{\mu _1,\mu _2}^{13} \Vert \le&\sup _{-2<p_1<2} h(p_1)\int _{-2}^2 \frac{1}{2}\frac{\chi _{13}}{p_1q_1+s_1s_2} \frac{1}{h(q_1)} {\text {d}}q_1\\=&\sup _{-\frac{\sqrt{\mu _2}}{2}+\sqrt{\mu _1}<p_1<\frac{3\sqrt{\mu _2}}{2}} \Bigg [ h(p_1)\times \\  &\qquad \int _{\max \{\sqrt{\mu _1}-p_1,0, -\sqrt{\mu _2}+p_1\}}^{\frac{\sqrt{\mu _2}}{2}} \frac{1}{p_1q_1+s_1s_2} \frac{1}{\vert s_2-q_1 \vert ^{1/2}} {\text {d}}q_1\Bigg ]. \end{aligned} \end{aligned}$$

For $p_1>\sqrt{\mu _1}, q_1>0$, we have $p_1q_1+s_1 s_2 \ge \sqrt{\mu _1}(q_1+s_2)$. Therefore,

7.42 $$\begin{aligned}  &   \sup _{\sqrt{\mu _1}<p_1<\sqrt{\mu _2}+s_2} h(p_1) \int _{\max \{\sqrt{\mu _1}-p_1,0, -\sqrt{\mu _2}+p_1\}}^{\frac{\sqrt{\mu _2}}{2}} \frac{1}{p_1q_1+s_1s_2} \frac{1}{\vert s_2-q_1 \vert ^{1/2}} {\textrm{d}}q_1\nonumber \\  &   \quad \le \sup _{\sqrt{\mu _1}<p_1<\sqrt{\mu _2}+s_2} \frac{ \vert p_1-s_1 \vert ^{1/2}}{\sqrt{\mu _1}} \int _{0}^{\infty } \frac{1}{q_1+s_2} \frac{1}{\vert s_2-q_1 \vert ^{1/2}} {\textrm{d}}q_1\nonumber \\  &   \quad =\frac{2^{1/2}}{\sqrt{\mu _1}} \int _{0}^{\infty } \frac{1}{q_1+1} \frac{1}{\vert 1-q_1 \vert ^{1/2}} {\textrm{d}}q_1\nonumber \\  &   \quad \le \frac{2^{1/2}}{\sqrt{\mu _1}}\left[ \int _{0}^{2}\frac{1}{\vert 1-q_1 \vert ^{1/2}} {\textrm{d}}q_1+\int _{2}^{\infty } \frac{1}{\vert q_1-1 \vert ^{3/2}} {\textrm{d}}q_1 \right] =\frac{2^{1/2}6}{\sqrt{\mu _1}} \end{aligned}$$

and

7.43 $$\begin{aligned}&\sup _{\sqrt{\mu _2}+s_2<p_1<\frac{3\sqrt{\mu _2}}{2}} h(p_1) \int _{\max \{\sqrt{\mu _1}-p_1,0, -\sqrt{\mu _2}+p_1\}}^{\frac{\sqrt{\mu _2}}{2}} \frac{1}{p_1q_1+s_1s_2} \frac{1}{\vert s_2-q_1 \vert ^{1/2}} {\textrm{d}}q_1\nonumber \\&\quad \le \sup _{\sqrt{\mu _2}+s_2<p_1<\frac{3\sqrt{\mu _2}}{2}} \frac{ \vert p_1-s_1 \vert ^{1/2}}{\sqrt{\mu _1}} \int _{-\sqrt{\mu _2}+p_1}^{\infty } \frac{1}{q_1+s_2} \frac{1}{\vert q_1-s_2 \vert ^{1/2}} {\textrm{d}}q_1\nonumber \\&\quad =\sup _{2s_2<x<\mu _2-\frac{\sqrt{\mu _1}}{2}} \frac{ \vert x \vert ^{1/2}}{\sqrt{\mu _1}} \int _{x}^{\infty } \frac{1}{y} \frac{1}{\vert y-2s_2 \vert ^{1/2}} {\textrm{d}}y\nonumber \\&\quad =\sup _{2s_2<x<\mu _2-\frac{\sqrt{\mu _1}}{2}} \frac{1}{\sqrt{\mu _1}} \int _{1}^{\infty } \frac{1}{y} \frac{1}{\vert y-\frac{2s_2}{x} \vert ^{1/2}} {\textrm{d}}y=\frac{1}{\sqrt{\mu _1}} \int _{1}^{\infty } \frac{1}{y} \frac{1}{\vert y-1 \vert ^{1/2}} {\textrm{d}}y\nonumber \\&\quad \le \frac{1}{\sqrt{\mu _1}}\left[ \int _{1}^{2}\frac{1}{\vert y-1 \vert ^{1/2}}{\textrm{d}}y+ \int _{2}^{\infty } \frac{1}{\vert y-1 \vert ^{3/2}}{\textrm{d}}y\right] =\frac{4}{\sqrt{\mu _1}}, \end{aligned}$$

where we substituted $x=p_1-s_1$ and $y=q_1+s_2$. Next, we consider the case $p_1<\frac{\sqrt{\mu _1}}{2}$. For $\frac{\sqrt{\mu _2}}{2}\ge q_1 \ge \sqrt{\mu _1}-p_1$ and $-s_2<p_1<\frac{\sqrt{\mu _1}}{2}$ we have

7.44 $$\begin{aligned} p_1 q_1+s_1 s_2\ge &   \left\{ \begin{array}{cc} \frac{\sqrt{\mu _1}}{2}(p_1+s_2)&  \textrm{if}\ p_1>0\\ (s_1-q_1)(p_1+s_2)-p_1(s_1-q_1)+q_1(p_1+s_2) &  \textrm{if}\ p_1<0 \end{array}\right. \nonumber \\\ge &   \left\{ \begin{array}{cc}\frac{\sqrt{\mu _1}}{2}(p_1+s_2) &  \textrm{if}\ p_1>0\\ (s_1-q_1)(p_1+s_2) &  \textrm{if}\ p_1<0 \end{array}\right. \ge \frac{\sqrt{\mu _1}}{2}(p_1+s_2). \end{aligned}$$

Therefore,

7.45 $$\begin{aligned}  &   \sup _{-\frac{\sqrt{\mu _2}}{2}+\sqrt{\mu _1}<p_1<\frac{\sqrt{\mu _1}}{2}} h(p_1) \int _{\max \{\sqrt{\mu _1}-p_1,0, -\sqrt{\mu _2}+p_1\}}^{\frac{\sqrt{\mu _2}}{2}} \frac{1}{p_1q_1+s_1s_2} \frac{1}{\vert s_2-q_1 \vert ^{1/2}} {\textrm{d}}q_1\nonumber \\  &   \quad \le \sup _{-\frac{\sqrt{\mu _2}}{2}+\sqrt{\mu _1}<p_1<\frac{\sqrt{\mu _1}}{2}} \frac{2 h(p_1)}{\sqrt{\mu _1}(p_1+s_2)} \int _{\sqrt{\mu _1}-p_1}^{\frac{\sqrt{\mu _2}}{2}} \frac{1}{\vert s_2-q_1 \vert ^{1/2}} {\textrm{d}}q_1\nonumber \\  &   \quad \le \sup _{-\frac{\sqrt{\mu _2}}{2}+\sqrt{\mu _1}<p_1<\frac{\sqrt{\mu _1}}{2}} \frac{4}{\sqrt{\mu _1}(p_1+s_2)^{1/2}}\nonumber \\  &   \qquad \times \left\{ \begin{matrix}\left( \frac{\sqrt{\mu _1}}{2}\right) ^{1/2} &  \textrm{if}\ \sqrt{\mu _1}-p_1>s_2\\ \left( \frac{\sqrt{\mu _1}}{2}\right) ^{1/2}+ (s_2-\sqrt{\mu _1}+p_1)^{1/2}&  \textrm{if}\ \sqrt{\mu _1}-p_1<s_2\end{matrix} \right. . \end{aligned}$$

Note that $\sup _{-\frac{\sqrt{\mu _2}}{2}+\sqrt{\mu _1}<p_1<\frac{\sqrt{\mu _1}}{2}} (p_1+s_2)^{-1/2} \left( \frac{\sqrt{\mu _1}}{2}\right) ^{1/2} =1$ and that for $p_1>\sqrt{\mu _1}-s_2$ we have $\left| \frac{s_2-\sqrt{\mu _1}+p_1}{p_1+s_2} \right| \le 1$. One can hence bound (7.45) above by $\frac{8}{\sqrt{\mu _1}}$.

For $q_1 \ge 0$ and $p_1>\frac{\sqrt{\mu _1}}{2}$ we have $p_1 q_1+s_1 s_2 \ge \frac{\sqrt{\mu _1}}{2}(q_1+s_2)$. Therefore,

7.46 $$\begin{aligned}  &   \sup _{\frac{\sqrt{\mu _1}}{2}<p_1<\sqrt{\mu _1}} h(p_1) \int _{\max \{\sqrt{\mu _1}-p_1,0, -\sqrt{\mu _2}+p_1\}}^{\frac{\sqrt{\mu _2}}{2}} \frac{1}{p_1q_1+s_1s_2} \frac{1}{\vert s_2-q_1 \vert ^{1/2}} {\textrm{d}}q_1\nonumber \\  &   \quad \le \sup _{\frac{\sqrt{\mu _1}}{2}<p_1<\sqrt{\mu _1}} \frac{2h(p_1)}{\sqrt{\mu _1}} \int _{\sqrt{\mu _1}-p_1}^{\infty } \frac{1}{(q_1+s_2)\vert s_2-q_1 \vert ^{1/2}} {\textrm{d}}q_1\nonumber \\  &   \quad =\sup _{\frac{\sqrt{\mu _1}}{2}<p_1<\sqrt{\mu _1}} \frac{4h(p_1)}{\sqrt{\mu _1} \sqrt{2s_2}} \left\{ \begin{matrix} {{\,\textrm{artanh}\,}}\left( \sqrt{\frac{s_2-\sqrt{\mu _1}+p_1}{2 s_2}}\right) +\pi & \textrm{if}\ s_2>\sqrt{\mu _1}-p_1\\ \arctan \left( \sqrt{\frac{2 s_2}{\sqrt{\mu _1}-p_1-s_2}}\right) &  \textrm{if}\ s_2<\sqrt{\mu _1}-p_1\end{matrix}\right. \nonumber \\ \end{aligned}$$

We estimate the two cases separately:

7.47 $$\begin{aligned}  &   \sup _{\sqrt{\mu _1}-s_2<p_1<\sqrt{\mu _1}} \frac{4h(p_1)}{\sqrt{\mu _1} \sqrt{2s_2}} \left[ {{\,\textrm{artanh}\,}}\left( \sqrt{\frac{s_2-\sqrt{\mu _1}+p_1}{2 s_2}}\right) +\pi \right] \nonumber \\  &   \quad \le \frac{4 \vert s_1-\sqrt{\mu _1}+s_2 \vert ^{1/2}}{\sqrt{\mu _1} \sqrt{2s_2}} \left[ {{\,\textrm{artanh}\,}}\left( \frac{1}{\sqrt{2}}\right) +\pi \right] =4\frac{{{\,\textrm{artanh}\,}}\left( \frac{1}{\sqrt{2}}\right) +\pi }{\sqrt{\mu _1}} \nonumber \\ \end{aligned}$$

and

7.48 $$\begin{aligned} \begin{aligned}&\sup _{\frac{\sqrt{\mu _1}}{2}<p_1<\sqrt{\mu _1}-s_2} \frac{4h(p_1)}{\sqrt{\mu _1} \sqrt{2s_2}} \arctan \left( \sqrt{\frac{2 s_2}{\sqrt{\mu _1}-p_1-s_2}}\right) \\  &\quad \le \frac{4}{\sqrt{\mu _1}}\sup _{\frac{\sqrt{\mu _1}}{2}<p_1<\sqrt{\mu _1}-s_2}\Bigg [ \frac{\vert s_1-p_1 \vert ^{1/2}-\vert \sqrt{\mu _1}-p_1-s_2 \vert ^{1/2}}{ \sqrt{2s_2}}\frac{\pi }{2} \\  &\qquad + \frac{\vert \sqrt{\mu _1}-p_1-s_2 \vert ^{1/2}}{\sqrt{2s_2}} \arctan \left( \sqrt{\frac{2 s_2}{\sqrt{\mu _1}-p_1-s_2}}\right) \Bigg ]\\  &\quad \le \frac{4}{\sqrt{\mu _1}}\sup _{\frac{\sqrt{\mu _1}}{2}<p_1<\sqrt{\mu _1}-s_2}\left[ \frac{\sqrt{2s_2}}{\vert s_1-p_1 \vert ^{1/2}+\vert \sqrt{\mu _1}-p_1-s_2 \vert ^{1/2}}\frac{\pi }{2} +1\right] \\  &\quad \le \frac{4 (\frac{\pi }{2}+1)}{\sqrt{\mu _1}}. \end{aligned} \end{aligned}$$

In total, we obtain

7.49 $$\begin{aligned} \Vert D_{\mu _1,\mu _2}^{13} \Vert\le &   \max \left\{ \frac{2^{1/2} 6}{\mu _1^{1/2}},\frac{4}{\mu _1^{1/2}},\frac{8}{\mu _1^{1/2}},\frac{4({{\,\textrm{artanh}\,}}(1/\sqrt{2})+\pi )}{\mu _1^{1/2}},\frac{4 (\pi /2+1)}{\mu _1^{1/2}}\right\} \nonumber \\= &   \frac{4({{\,\textrm{artanh}\,}}(1/\sqrt{2})+\pi )}{\mu _1^{1/2}}. \end{aligned}$$

**Region 14:** By symmetry in $p_1$, we have

7.50 $$\begin{aligned} \begin{aligned} \Vert D_{\mu _1,\mu _2}^{14} \Vert&\le \sup _{-2<p_1<2} h(p_1)\int _{-2}^2 \frac{\chi _{14}}{s_1^2+s_2^2-p_1^2-q_1^2} \frac{1}{h(q_1)} {\text {d}}q_1 \\  &= \sup _{0<p_1<s_1}h(p_1) \int _{\max \{-\sqrt{\mu _1}-p_1,-\sqrt{\mu _2}/2,-\sqrt{\mu _2}+p_1\}}^{\min \{\sqrt{\mu _1}-p_1,\frac{\sqrt{\mu _2}}{2}\}} \frac{1}{s_1^2+s_2^2-p_1^2-q_1^2} \\  &\quad \times \frac{1}{\vert \vert q_1\vert -s_2 \vert ^{1/2}} {\text {d}}q_1 \\  &\le \sup _{0<p_1<s_1}2 h(p_1) \int _{\max \{0,p_1-\sqrt{\mu _1}\}}^{\min \{\sqrt{\mu _1}+p_1,\frac{\sqrt{\mu _2}}{2},\sqrt{\mu _2}-p_1\}} \frac{1}{s_1^2+s_2^2-p_1^2-q_1^2} \\  &\quad \times \frac{1}{\vert \vert q_1\vert -s_2 \vert ^{1/2}} {\text {d}}q_1, \end{aligned} \end{aligned}$$

where in the last inequality we increased the domain to be symmetric in $q_1$ and used the symmetry of the integrand.

For $p_1 \le s_2$ and $\sqrt{\mu _1}+p_1>q_1$ we have $s_1^2+s_2^2-p_1^2-q_1^2 \ge s_1^2+s_2^2-p_1^2-(\sqrt{\mu _1}+p_1)^2=2 (s_2-p_1)(p_1+s_1)$. Hence,

7.51 $$\begin{aligned}  &   \sup _{0<p_1<\frac{\sqrt{\mu _2}}{2}-\sqrt{\mu _1}}2 h(p_1) \int _{0}^{\sqrt{\mu _1}+p_1} \frac{1}{s_1^2+s_2^2-p_1^2-q_1^2} \frac{1}{\vert \vert q_1\vert -s_2 \vert ^{1/2}} {\textrm{d}}q_1\nonumber \\  &   \quad \le \sup _{0<p_1<\frac{\sqrt{\mu _2}}{2}-\sqrt{\mu _1}} \frac{1}{(s_2-p_1)^{1/2}(p_1+s_1)} \int _{0}^{\sqrt{\mu _1}+p_1} \frac{1}{\vert \vert q_1\vert -s_2 \vert ^{1/2}} {\textrm{d}}q_1\nonumber \\  &   \quad =\sup _{0<p_1<\frac{\sqrt{\mu _2}}{2}-\sqrt{\mu _1}}\frac{2(s_2^{1/2}+(p_1+\sqrt{\mu _1}-s_2)^{1/2})}{(s_2-p_1)^{1/2}(p_1+s_1)} \le \frac{2(s_2^{1/2}+\left( \frac{\sqrt{\mu _1}}{2}\right) ^{1/2})}{\left( \frac{\sqrt{\mu _1}}{2}\right) ^{1/2}s_1}\nonumber \\  &   \quad \le \frac{4\left( \frac{\sqrt{\mu _2}}{2}\right) ^{1/2}}{\left( \frac{\sqrt{\mu _1}}{2}\right) ^{1/2}\frac{\sqrt{\mu _2}}{2}}\le \frac{8}{\sqrt{\mu _1}}. \end{aligned}$$

Similarly, for $p_1 \ge s_2$ and $\sqrt{\mu _2}-p_1>q_1$ we have $s_1^2+s_2^2-p_1^2-q_1^2 \ge s_1^2+s_2^2-p_1^2-(\sqrt{\mu _2}-p_1)^2=2 (s_1-p_1)(p_1-s_2)$. Therefore,

7.52 $$\begin{aligned}  &   \sup _{\frac{\sqrt{\mu _2}}{2}<p_1<s_1}2 h(p_1) \int _{p_1-\sqrt{\mu _1}}^{\sqrt{\mu _2}-p_1} \frac{1}{s_1^2+s_2^2-p_1^2-q_1^2} \frac{1}{\vert q_1-s_2 \vert ^{1/2}} {\textrm{d}}q_1 \nonumber \\  &   \quad \le \sup _{\frac{\sqrt{\mu _2}}{2}<p_1<s_1} \frac{1}{(s_1-p_1)^{1/2}(p_1-s_2)} \int _{p_1-\sqrt{\mu _1}}^{\sqrt{\mu _2}-p_1} \frac{1}{\vert q_1-s_2 \vert ^{1/2}} {\textrm{d}}q_1 \nonumber \\  &   \quad =\sup _{\frac{\sqrt{\mu _2}}{2}<p_1<s_1} \frac{4}{p_1-s_2} =\frac{8}{\sqrt{\mu _1}}. \end{aligned}$$

For $\frac{\sqrt{\mu _2}}{2}-\sqrt{\mu _1}\le p_1\le \frac{\sqrt{\mu _2}}{2}$ and $q_1<\frac{\sqrt{\mu _2}}{2}$, we have $s_1^2+s_2^2-p_1^2-q_1^2\ge \frac{\mu _1}{2}$. Thus,

7.53 $$\begin{aligned}  &   \sup _{\frac{\sqrt{\mu _2}}{2}-\sqrt{\mu _1}< p_1<\frac{\sqrt{\mu _2}}{2}}2 h(p_1) \int _{\max \{0,p_1-\sqrt{\mu _1}\}}^{\frac{\sqrt{\mu _2}}{2}} \frac{1}{s_1^2+s_2^2-p_1^2-q_1^2} \frac{1}{\vert q_1-s_2 \vert ^{1/2}} {\textrm{d}}q_1 \nonumber \\  &   \quad \le \frac{4}{\mu _1} \left( \frac{\sqrt{\mu _1}}{2}\right) ^{1/2} \int _{\frac{\sqrt{\mu _2}}{2}-2\sqrt{\mu _1}}^{\frac{\sqrt{\mu _2}}{2}}\frac{1}{\vert q_1-s_2 \vert ^{1/2}} {\textrm{d}}q_1 \nonumber \\  &   \quad =\frac{8 \left( \left( \frac{3\sqrt{\mu _1}}{2}\right) ^{1/2}+\left( \frac{\sqrt{\mu _1}}{2}\right) ^{1/2}\right) }{2^{1/2}\mu _1^{3/4}} \le \frac{4}{\mu _1^{1/2}}\left( \sqrt{3}+1\right) . \end{aligned}$$

In total, we have

7.54 $$\begin{aligned} \Vert D_{\mu _1,\mu _2}^{14} \Vert \le \frac{4}{\mu _1^{1/2}}\left( \sqrt{3}+1\right) . \end{aligned}$$

$$\square $$

### Proof of Lemma 6.6

#### Proof of Lemma 6.6

The integral in (6.23) is invariant under rotations of ${\tilde{q}}$. Therefore, it suffices to take the supremum over ${\tilde{q}}=q_2\ge 0$ for $d=2$ and ${\tilde{q}} =(q_2,0)$ with $q_2\ge 0$ for $d=3$. Furthermore, it suffices to restrict to $p_2\ge 0$ since the integrand is invariant under ${\tilde{p}} \rightarrow -{\tilde{p}}$. Note that under these conditions $\mu _1 \le \mu _2$. We split the domain of integration in (6.23) into two regions according to $\mu _1=\min \{\mu _1,\mu _2\} \lessgtr 0$.

*Dimension three:* We first consider the case $\mu _1<0$, i.e. $\vert p_2+q_2\vert ^2>1-p_3^2$. In this case,

7.55 $$\begin{aligned}  &   \sup _{{\tilde{q}}=(q_2,0), q_2\ge 0}\int _{{\mathbb {R}}^{2}} \chi _{\vert {\tilde{p}} \vert<2 } \chi _{p_2\ge 0}\frac{\chi _{\min \{\mu _1,\mu _2\}<0}}{(-\min \{\mu _1,\mu _2\})^{\alpha }}{\textrm{d}}{\tilde{p}}\nonumber \\  &   \quad =\sup _{q_2\ge 0}\Bigg [\int _{-1}^1{\textrm{d}}p_3 \int _{\max \{\sqrt{1-p_3^2}-q_2,0\}}^{\sqrt{4-p_3^2}} \frac{1}{((p_2+q_2)^2+p_3^2-1)^{\alpha }}{\textrm{d}}p_2 \nonumber \\  &   \qquad +\int _{1<|p_3|<2}{\textrm{d}}p_3 \int _{0}^{\sqrt{4-p_3^2}} \frac{1}{((p_2+q_2)^2+p_3^2-1)^\alpha }{\textrm{d}}p_2\Bigg ]. \end{aligned}$$

Let $q_2$ and $|p_3|\le 1$ be fixed. By substituting $x=p_2+q_2-\sqrt{1-p_3^2}$ if $q_2\le \sqrt{1-p_3^2}$, one obtains

7.56 $$\begin{aligned}  &   \int _{\max \{\sqrt{1-p_3^2}-q_2,0\}}^{2} \frac{1}{((p_2+q_2)^2+p_3^2-1)^{\alpha }}{\textrm{d}}p_2\nonumber \\  &   \quad \le \int _{0}^{2} \frac{ \chi _{q_2\le \sqrt{1-p_3^2}}}{(x+\sqrt{1-p_3^2})^2+p_3^2-1)^{\alpha }}{\textrm{d}}x\nonumber \\  &   \qquad +\int _{0}^{2} \frac{\chi _{q_2>\sqrt{1-p_3^2}}}{((p_2+\sqrt{1-p_3^2})^2+p_3^2-1)^{\alpha }}{\textrm{d}}p_2\nonumber \\  &   \quad \le \int _{0}^{2} \frac{1}{(2p_2\sqrt{1-p_3^2})^{\alpha }}{\textrm{d}}p_2 \le \frac{C}{(1-p_3^2)^{\alpha /2}} \end{aligned}$$

for some finite constant *C*. Since $\int _{-1}^1(1-p_3^2)^{-\alpha /2}{\textrm{d}}p_3<\infty$, the first term in (7.55) is bounded. The second term is bounded by

7.57 $$\begin{aligned} \int _{1<|p_3|<2}{\textrm{d}}p_3 \int _{0}^{2} \frac{1}{(p_3^2-1)^\alpha }{\textrm{d}}p_2<\infty . \end{aligned}$$

For the case $\mu _1>0$ we have $\vert p_2+q_2\vert ^2<1-p_3^2$. Hence,

7.58 $$\begin{aligned}  &   \sup _{{{\tilde{q}}=(q_2,0), q_2\ge 0}} \int _{{\mathbb {R}}^{2}} \chi _{\vert {\tilde{p}} \vert<2 }\chi _{p_2\ge 0} \frac{\chi _{0<\min \{\mu _1,\mu _2\}}}{\min \{\mu _1,\mu _2\}^{\alpha }}{\textrm{d}}{\tilde{p}}\nonumber \\  &   \quad =\sup _{q_2\ge 0} \int _{-1}^1 {\textrm{d}}p_3 \chi _{q_2\le \sqrt{1-p_3^2}} \int _0^{\sqrt{1-p_3^2}-q_2} \frac{1}{(1-(p_2+q_2)^2-p_3^2)^\alpha } {\textrm{d}}p_2.\nonumber \\ \end{aligned}$$

For fixed $|p_3|<1$ and $q_2\le \sqrt{1-p_3^2}$ substituting $x=\sqrt{1-p_3^2}-q_2-p_2$ gives

7.59 $$\begin{aligned}  &   \int _0^{\sqrt{1-p_3^2}-q_2} \frac{1}{(1-(p_2+q_2)^2-p_3^2)^\alpha } {\textrm{d}}p_2\nonumber \\  &   \quad =\int _0^{\sqrt{1-p_3^2}-q_2} \frac{1}{(1-(\sqrt{1-p_3^2}-x)^2-p_3^2)^\alpha } {\textrm{d}}x\nonumber \\  &   \quad =\int _0^{\sqrt{1-p_3^2}-q_2} \frac{1}{x^\alpha (2\sqrt{1-p_3^2}-x)^\alpha } {\textrm{d}}x. \end{aligned}$$

Thus the expression in (7.58) is bounded by

7.60 $$\begin{aligned}  &   \sup _{q_2\ge 0} \int _{-1}^1 {\textrm{d}}p_3 \chi _{q_2\le \sqrt{1-p_3^2}} \int _0^{\sqrt{1-p_3^2}-q_2} \frac{1}{x^\alpha (\sqrt{1-p_3^2}+q_2)^\alpha } {\textrm{d}}x\nonumber \\  &   \quad \le \int _{-1}^1 {\textrm{d}}p_3 \int _0^{1} \frac{1}{x^\alpha (\sqrt{1-p_3^2})^\alpha }{\textrm{d}}x<\infty . \end{aligned}$$

*Dimension two:* For the case $\mu _1<0$ we have $\vert p_2+q_2\vert >1$. Hence,

7.61 $$\begin{aligned} \sup _{q_2\ge 0} \int _{0}^2\frac{\chi _{\min \{\mu _1,\mu _2\}<0}}{(-\min \{\mu _1,\mu _2\})^{\alpha }}{\textrm{d}}p_2=\sup _{q_2\ge 0}\int _{\max \{1-q_2,0\}}^{2} \frac{1}{((p_2+q_2)^2-1)^\alpha } {\textrm{d}}p_2.\nonumber \\ \end{aligned}$$

This is finite according to (7.56).

For the case $\mu _1>0$,

7.62 $$\begin{aligned} \sup _{q_2\ge 0}\int _{0}^2\frac{\chi _{0<\min \{\mu _1,\mu _2\}}}{\min \{\mu _1,\mu _2\}^{\alpha }}{\textrm{d}}p_2= &   \sup _{0 \le q_2\le 1}\int _{0}^{1-q_2}\frac{1}{(1-(p_2+q_2)^2)^\alpha }{\textrm{d}}p_2 \nonumber \\= &   \int _{0}^{1}\frac{1}{x^\alpha (2-x)^\alpha }{\textrm{d}}x <\infty , \end{aligned}$$

where we used (7.59) in the second equality. $\square$

### Proof of Lemma 6.7

#### Proof of Lemma 6.7

The proof follows from elementary computations. We carry out the case $d=2$ and leave the case $d=3$, where one additional integration over $q_3$ needs to be performed, to the reader.

By symmetry, we may restrict to $p_1,q_1,p_2\ge 0$. Furthermore, we will partition the remaining domain of $p_2,q_2$ into nine subdomains. Let $\chi _j$ be the characteristic function of domain *j*. Since $(a+b)^2\le 2(a^2+b^2)$, there is a constant *C* such that the expression in (6.29) is bounded above by $C \sum _{j=1}^9 \lim _{\epsilon \rightarrow 0} I_j$, where

7.63 $$\begin{aligned} I_j= \sup _{0\le p_2<\epsilon }\int _{{\mathbb {R}}^2} \chi _{0<p_1,q_1<\epsilon } \left[ \int _{-\sqrt{2}}^{\sqrt{2}} \frac{2\chi _j}{\vert (p+q)^2-1 \vert + \vert (p-q)^2-1 \vert } {\textrm{d}}q_2 \right] ^2 {\textrm{d}}p_1 {\textrm{d}}q_1. \end{aligned}$$

Hence, we can consider the domains case by case and prove that $\lim _{\epsilon \rightarrow 0} I_j=0$ for each of them.

**Fig. 5:**
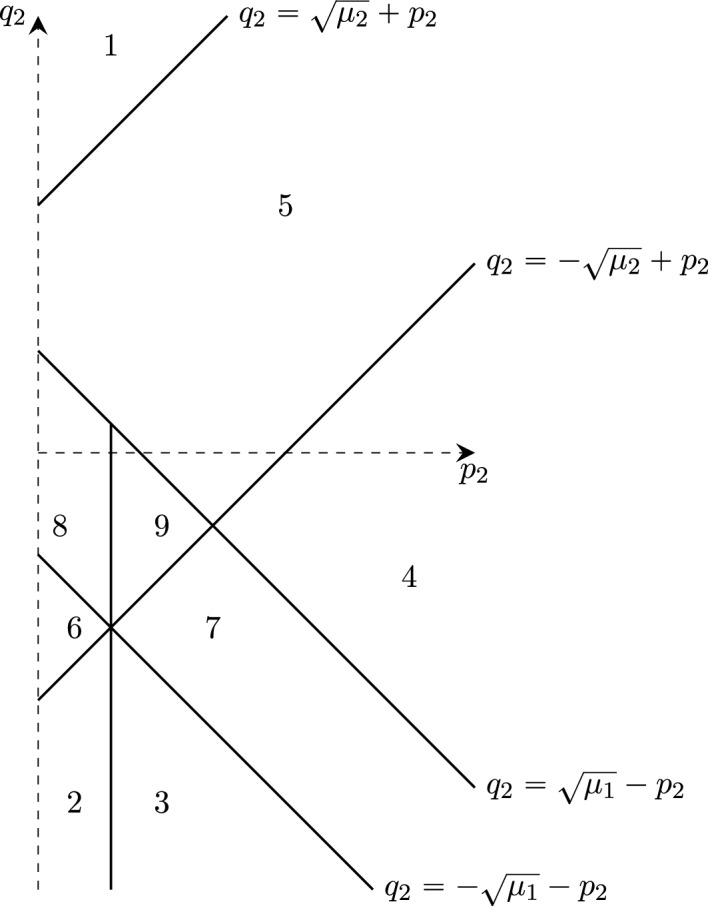
Domains occurring in the proof of Lemma 6.7

We use the notation $\mu _1=1-(p_1+q_1)^2$ and $\mu _2=1-(p_1-q_1)^2$. (Note that this differs from the notation in Lemma 6.5). Since $p_1,q_1\ge 0$ we have $\mu _2\ge \mu _1$. We assume that $\epsilon <1/4$, and thus for $p_1,q_1<\epsilon$ we have $\mu _1,\mu _2>1-4\epsilon ^2>3/4$.

For fixed $0<p_1,q_1<\epsilon$, we choose the subdomains for $p_2,q_2$ as sketched in Fig. 5. The subdomains are chosen according to the signs of $(p_2+q_2)^2-\mu _1$ and $(p_2-q_2)^2-\mu _2$, and to distinguish which of $-\sqrt{\mu _1}-p_2,-\sqrt{\mu _2}+p_2$ is larger.

We start with domains 1 to 4, where $(p+q)^2-1=(p_2+q_2)^2-\mu _1>0$ and $(p-q)^2-1=(p_2-q_2)^2-\mu _2>0$. Note that in domain 4, $p_2\ge \frac{\sqrt{\mu _1}+\sqrt{\mu _2}}{2}\ge \sqrt{1-4\epsilon ^2}$, which is larger than $\epsilon$. Hence $\chi _4=0$ for $p_2<\sqrt{1-4\epsilon ^2}$, giving $I_4=0$. For domains 2 and 3, we have

7.64 $$\begin{aligned} I_2&=\sup _{0\le p_2<\epsilon }\int _{{\mathbb {R}}^2} \chi _{0<p_1,q_1<\epsilon }\chi _{2p_2<\sqrt{\mu _2}-\sqrt{\mu _1}} \nonumber \\&\quad \times \left[ \int _{a_2}^{2} \frac{1}{p_1^2+q_1^2+p_2^2+q_2^2-1} {\textrm{d}}q_2 \right] ^2 {\textrm{d}}p_1 {\textrm{d}}q_1,\end{aligned}$$

7.65 $$\begin{aligned} I_3&=\sup _{0\le p_2<\epsilon }\int _{{\mathbb {R}}^2} \chi _{0<p_1,q_1<\epsilon }\chi _{2p_2>\sqrt{\mu _2}-\sqrt{\mu _1}}\nonumber \\&\quad \times \left[ \int _{a_3}^{2} \frac{1}{p_1^2+q_1^2+p_2^2+q_2^2-1} {\textrm{d}}q_2 \right] ^2 {\textrm{d}}p_1 {\textrm{d}}q_1, \end{aligned}$$

where $a_2=\sqrt{\mu _2}-p_2$ and $a_3=\sqrt{\mu _1}+p_2$. Since $0\le p_1,q_1,p_2<\epsilon$, we have $1-p_1^2-q_1^2-p_2^2>3/4$ and thus

7.66 $$\begin{aligned} \int _{a_j}^{2} \frac{1}{p_1^2+q_1^2+p_2^2+q_2^2-1}= &   \frac{{{\,\textrm{artanh}\,}}\frac{\sqrt{1-p_1^2-q_1^2-p_2^2}}{a_j}-{{\,\textrm{artanh}\,}}\frac{\sqrt{1-p_1^2-q_1^2-p_2^2}}{2}}{\sqrt{1-p_1^2-q_1^2-p_2^2}}\nonumber \\\le &   C {{\,\textrm{artanh}\,}}\frac{\sqrt{1-p_1^2-q_1^2-p_2^2}}{a_j}. \end{aligned}$$

Since ${{\,\textrm{artanh}\,}}(x/y)=\ln ((y+x)^2/(y^2-x^2))/2$ and $\sqrt{1-p_1^2-q_1^2-p_2^2}+a_j\le 3$ we get

7.67 $$\begin{aligned} I_2\le &   \frac{C}{2} \sup _{0\le p_2<\epsilon }\int _{{\mathbb {R}}^2} \chi _{0<p_1,q_1<\epsilon }\chi _{2p_2<\sqrt{\mu _2}-\sqrt{\mu _1}} \nonumber \\  &   \times \left[ \ln \frac{9}{2(p_1 q_1-p_2(\sqrt{\mu _2}-p_2))} \right] ^2 {\textrm{d}}p_1 {\textrm{d}}q_1 \end{aligned}$$

and

7.68 $$\begin{aligned} I_3\le &   \frac{C}{2} \sup _{0\le p_2<\epsilon }\int _{{\mathbb {R}}^2} \chi _{0<p_1,q_1<\epsilon }\chi _{2p_2>\sqrt{\mu _2}-\sqrt{\mu _1}} \nonumber \\  &   \times \left[ \ln \frac{9}{2(p_1 q_1+p_2(\sqrt{\mu _1}+p_2))} \right] ^2 {\textrm{d}}p_1 {\textrm{d}}q_1. \end{aligned}$$

For domain 2, we substitute $z=p_1+q_1$ and $r=p_1-q_1$ and obtain the bound

7.69 $$\begin{aligned} I_2\le &   \frac{C}{4} \sup _{0\le p_2<\epsilon }\int _{{\mathbb {R}}^2} \chi _{\vert r\vert<z<2\epsilon }\chi _{2p_2<\sqrt{\mu _2}-\sqrt{\mu _1}}\nonumber \\  &   \times \left[ \ln \frac{18}{z^2-r^2-4p_2(\sqrt{1-r^2}-p_2)} \right] ^2 {\textrm{d}}r {\textrm{d}}z. \end{aligned}$$

The condition $2p_2<\sqrt{\mu _2}-\sqrt{\mu _1}$ implies that $x:=z^2-r^2-4p_2(\sqrt{1-r^2}-p_2)\ge 0$. Substituting *z* by *x* gives

7.70 $$\begin{aligned} I_2\le &   \frac{C}{4} \sup _{0\le p_2<\epsilon }\int _{-2\epsilon }^{2\epsilon }{\textrm{d}}r \int _{0}^{\epsilon ^2} {\textrm{d}}x \left[ \ln \frac{18}{x} \right] ^2 \frac{1}{2\sqrt{x+r^2+4p_2(\sqrt{1-r^2}-p_2)}}\nonumber \\\le &   \frac{C}{2}\epsilon \int _{0}^{\epsilon ^2} \left[ \ln \frac{18}{x} \right] ^2 \frac{1}{\sqrt{x}}{\textrm{d}}x. \end{aligned}$$

This vanishes as $\epsilon \rightarrow 0$. For domain 3 we bound (7.68) by

7.71 $$\begin{aligned} I_3\le C \int _{{\mathbb {R}}^2} \chi _{0<p_1,q_1<\epsilon } \left[ \ln \frac{9}{2 p_1 q_1} \right] ^2 {\textrm{d}}p_1 {\textrm{d}}q_1, \end{aligned}$$

which vanishes in the limit $\epsilon \rightarrow 0$. For domain 1 note that since $\sqrt{\mu _2}+p_2\ge a_2,a_3$, we have $I_1\le I_2+I_3$.

Now consider domain 5, where $(p+q)^2-1=(p_2+q_2)^2-\mu _1>0$ and $(p-q)^2-1=(p_2-q_2)^2-\mu _2<0$. We have

7.72 $$\begin{aligned} I_5= \sup _{0\le p_2<\epsilon }\int _{{\mathbb {R}}^2} \chi _{0<p_1,q_1<\epsilon } \left[ \int _{\sqrt{\mu _1}-p_2}^{\sqrt{\mu _2}+p_2} \frac{1}{2(p_1q_1+p_2 q_2)} {\textrm{d}}q_2 \right] ^2 {\textrm{d}}p_1 {\textrm{d}}q_1.\nonumber \\ \end{aligned}$$

Integration over $q_2$ gives

7.73 $$\begin{aligned} \int _{\sqrt{\mu _1}-p_2}^{\sqrt{\mu _2}+p_2} \frac{1}{2(p_1q_1+p_2 q_2)} {\textrm{d}}q_2 =\frac{1}{2p_2}\ln \left( 1+ \frac{(\sqrt{\mu _2}-\sqrt{\mu _1})p_2+2p_2^2}{p_1 q_1+(\sqrt{\mu _1}-p_2)p_2}\right) .\nonumber \\ \end{aligned}$$

Note that $\sqrt{\mu _2}-\sqrt{\mu _1}= 4p_1q_1/(\sqrt{\mu _2}+\sqrt{\mu _1})\le 2 p_1 q_1 /\sqrt{1-4\epsilon ^2}$ and $\sqrt{\mu _1}-p_2\ge \sqrt{1-4\epsilon ^2}-\epsilon$. We can therefore bound the previous expression from above by

7.74 $$\begin{aligned} \frac{1}{2p_2}\ln \left( 1+ \frac{2 p_2}{\sqrt{1-4\epsilon ^2}}+\frac{2p_2}{\sqrt{1-4\epsilon ^2}-\epsilon }\right) \le \frac{1 }{\sqrt{1-4\epsilon ^2}}+\frac{1}{\sqrt{1-4\epsilon ^2}-\epsilon }<C,\nonumber \\ \end{aligned}$$

where we used that $\ln (1+ x)/x \le 1$ for $x\ge 0$. Therefore $I_5\le C^2 \epsilon ^2$ vanishes as $\epsilon \rightarrow 0$.

For region 6 we have

7.75 $$\begin{aligned} I_6= &   \sup _{0\le p_2<\epsilon }\int _{{\mathbb {R}}^2} \chi _{0<p_1,q_1<\epsilon }\chi _{2p_2\le \sqrt{\mu _2}-\sqrt{\mu _1}}\nonumber \\  &   \times \left[ \int _{-\sqrt{\mu _2}+p_2}^{-\sqrt{\mu _1}-p_2} \frac{1}{2(p_1 q_1+p_2 q_2)} {\textrm{d}}q_2 \right] ^2 {\textrm{d}}p_1 {\textrm{d}}q_1. \end{aligned}$$

Integration over $q_2$ gives

7.76 $$\begin{aligned} \int _{-\sqrt{\mu _2}+p_2}^{-\sqrt{\mu _1}-p_2} \frac{1}{2(p_1q_1+p_2 q_2)} {\textrm{d}}q_2 =\frac{1}{2p_2}\ln \left( 1+ p_2\frac{(\sqrt{\mu _2}-\sqrt{\mu _1}-2p_2)}{p_1 q_1-(\sqrt{\mu _2}-p_2)p_2}\right) .\nonumber \\ \end{aligned}$$

One can compute that

7.77 $$\begin{aligned} \frac{\partial }{\partial p_2}\frac{\sqrt{\mu _2}-\sqrt{\mu _1}-2p_2}{p_1 q_1-(\sqrt{\mu _2}-p_2)p_2}=\frac{8}{(\sqrt{\mu _2}+\sqrt{\mu _1}-2p_2)^2}>0. \end{aligned}$$

Thus, for $\chi _{2p_2\le \sqrt{\mu _2}-\sqrt{\mu _1}}$ we have

7.78 $$\begin{aligned} \frac{\sqrt{\mu _2}-\sqrt{\mu _1}-2p_2}{p_1 q_1-(\sqrt{\mu _2}-p_2)p_2}\le \lim _{p_2\rightarrow (\sqrt{\mu _2}-\sqrt{\mu _1})/2}\frac{\sqrt{\mu _2}-\sqrt{\mu _1}-2p_2}{p_1 q_1-(\sqrt{\mu _2}-p_2)p_2}=\frac{2}{\sqrt{\mu _1}}.\nonumber \\ \end{aligned}$$

The expression in (7.76) is thus bounded above by

7.79 $$\begin{aligned} \frac{1}{2p_2}\ln \left( 1+ p_2 \frac{2}{\sqrt{\mu _1}}\right) \le \frac{1}{\sqrt{\mu _1}}\le \frac{1}{\sqrt{1-4\epsilon ^2}}, \end{aligned}$$

which is bounded as $\epsilon \rightarrow 0$. In total, we have $I_6 \le C \epsilon ^2$, which vanishes in the limit $\epsilon \rightarrow 0$.

For region 7,

7.80 $$\begin{aligned} I_7= &   \sup _{0\le p_2<\epsilon }\int _{{\mathbb {R}}^2} \chi _{0<p_1,q_1<\epsilon }\chi _{2p_2\ge \sqrt{\mu _2}-\sqrt{\mu _1}}\nonumber \\  &   \times \left[ \int _{-\sqrt{\mu _1}-p_2}^{-\sqrt{\mu _2}+p_2} \frac{1}{-2(p_1 q_1+p_2 q_2)} {\textrm{d}}q_2 \right] ^2 {\textrm{d}}p_1 {\textrm{d}}q_1. \end{aligned}$$

Integration over $q_2$ gives

7.81 $$\begin{aligned} \int _{-\sqrt{\mu _1}-p_2}^{-\sqrt{\mu _2}+p_2} \frac{1}{-2(p_1 q_1+p_2 q_2)} {\textrm{d}}q_2 =\frac{1}{2p_2}\ln \left( 1+ p_2\frac{(\sqrt{\mu _2}-\sqrt{\mu _1}-2p_2)}{p_1 q_1-(\sqrt{\mu _2}-p_2)p_2}\right) .\nonumber \\ \end{aligned}$$

According to (7.77), for $(\sqrt{\mu _2}-\sqrt{\mu _1})/2\le p_2<\epsilon$ this is bounded by

7.82 $$\begin{aligned} \frac{1}{2p_2}\ln \left( 1+ p_2\frac{(\sqrt{\mu _2}-\sqrt{\mu _1}-2\epsilon )}{p_1 q_1-(\sqrt{\mu _2}-\epsilon )\epsilon }\right) \le \frac{1}{2} \frac{2\epsilon -(\sqrt{\mu _2}-\sqrt{\mu _1})}{(\sqrt{\mu _2}-\epsilon )\epsilon -p_1 q_1}. \end{aligned}$$

For $p_1,q_1<\epsilon$ this can be further estimated by

7.83 $$\begin{aligned} \frac{1}{2} \frac{2\epsilon }{(\sqrt{\mu _2}-\epsilon )\epsilon -\epsilon ^2} \le \frac{1}{\sqrt{1-4\epsilon ^2}-2\epsilon }, \end{aligned}$$

which is bounded for $\epsilon \rightarrow 0$. Hence, $I_7 \le C \epsilon ^2$ vanishes for $\epsilon \rightarrow 0$.

For domains 8 and 9, we have

7.84 $$\begin{aligned} I_8&=\sup _{0\le p_2<\epsilon }\int _{{\mathbb {R}}^2} \chi _{0<p_1,q_1<\epsilon }\chi _{2p_2<\sqrt{\mu _2}-\sqrt{\mu _1}}\nonumber \\&\quad \times \left[ \int _{-\sqrt{\mu _1}-p_2}^{\sqrt{\mu _1}-p_2} \frac{1}{1-p_1^2-q_1^2-p_2^2-q_2^2} {\textrm{d}}q_2 \right] ^2 {\textrm{d}}p_1 {\textrm{d}}q_1,\end{aligned}$$

7.85 $$\begin{aligned} I_9&=\sup _{0\le p_2<\epsilon }\int _{{\mathbb {R}}^2} \chi _{0<p_1,q_1<\epsilon }\chi _{2p_2>\sqrt{\mu _2}-\sqrt{\mu _1}}\nonumber \\&\quad \times \left[ \int _{-\sqrt{\mu _2}+p_2}^{\sqrt{\mu _1}-p_2} \frac{1}{1-p_1^2-q_1^2-p_2^2-q_2^2} {\textrm{d}}q_2 \right] ^2 {\textrm{d}}p_1 {\textrm{d}}q_1. \end{aligned}$$

We bound

7.86 $$\begin{aligned}  &   \int _{-\sqrt{\mu _1}-p_2}^{\sqrt{\mu _1}-p_2} \frac{1}{1-p_1^2-q_1^2-p_2^2-q_2^2} {\textrm{d}}q_2\le 2\int _{0}^{\sqrt{\mu _1}+p_2} \frac{1}{1-p_1^2-q_1^2-p_2^2-q_2^2} {\textrm{d}}q_2\nonumber \\  &   \quad =\frac{1}{\sqrt{1-p_1^2-q_1^2-p_2^2}}\ln \left( \frac{\sqrt{1-p_1^2-q_1^2-p_2^2}+\sqrt{\mu _1}+p_2}{\sqrt{1-p_1^2-q_1^2-p_2^2}-\sqrt{\mu _1}-p_2} \right) \nonumber \\  &   \quad =\frac{1}{\sqrt{1-p_1^2-q_1^2-p_2^2}}\ln \left( \frac{(\sqrt{1-p_1^2-q_1^2-p_2^2}+\sqrt{\mu _1}+p_2)^2}{2(p_1 q_1-p_2(\sqrt{\mu _1}+p_2))} \right) \nonumber \\  &   \quad \le \frac{1}{\sqrt{1-3\epsilon ^2}}\ln \left( \frac{9}{2(p_1 q_1-p_2(\sqrt{\mu _1}+p_2))} \right) . \end{aligned}$$

Substituting $z=p_1+q_1$ and $r=p_1-q_1$ we obtain

7.87 $$\begin{aligned} \begin{aligned} I_8\le&C \sup _{0\le p_2<\epsilon }\int _{{\mathbb {R}}^2} \chi _{0<p_1,q_1<\epsilon }\chi _{2p_2<\sqrt{\mu _2}-\sqrt{\mu _1}} \\  &\times \ln \left( \frac{9}{2(p_1 q_1-p_2(\sqrt{\mu _1}+p_2))} \right) ^2 {\text {d}}p_1 {\text {d}}q_1 \\  &\le \frac{C}{2}\sup _{0\le p_2<\epsilon }\int _{0}^{2\epsilon } {\text {d}}z \int _{-\epsilon }^\epsilon {\text {d}}r \chi _{|r|<z}\chi _{2p_2<\sqrt{1-r^2}-\sqrt{1-z^2}} \\  &\times \ln \left( \frac{18}{z^2-r^2-4p_2(\sqrt{1-z^2}+p_2)} \right) ^2. \end{aligned} \end{aligned}$$

Substituting *r* by $x=z^2-r^2-4p_2(\sqrt{1-z^2}+p_2)$ and using Hölder’s inequality, we obtain

7.88 $$\begin{aligned} I_8\le &   \frac{C}{4}\sup _{0\le p_2<\epsilon }\int _{4p_2-4p_2^2}^{2\epsilon } {\textrm{d}}z \int _{0}^{z^2-4p_2(p_2+\sqrt{1-z^2})} \ln \left( \frac{18}{x} \right) ^2 \nonumber \\  &   \times \frac{1}{\sqrt{z^2-4p_2(\sqrt{1-z^2}+p_2)-x}}{\textrm{d}}x\nonumber \\\le &   \frac{C}{4}\sup _{0\le p_2<\epsilon }\int _{4p_2-4p_2^2}^{2\epsilon } {\textrm{d}}z \Bigg [\left( \int _{0}^{z^2-4p_2(p_2+\sqrt{1-z^2})} \ln \left( \frac{18}{x} \right) ^6 {\textrm{d}}x \right) ^{1/3} \nonumber \\  &   \times \left( \int _{0}^{z^2-4p_2(p_2+\sqrt{1-z^2})} \frac{1}{(z^2-4p_2(\sqrt{1-z^2}+p_2)-x)^{3/4}}{\textrm{d}}x\right) ^{2/3}\Bigg ].\nonumber \\ \end{aligned}$$

In the last line we substitute $y=z^2-4p_2(\sqrt{1-z^2}+p_2)-x$, and then we use $z^2-4p_2(\sqrt{1-z^2}+p_2)-x\le 4\epsilon ^2$ to arrive at the bound

7.89 $$\begin{aligned} \begin{aligned} I_8&\le \frac{C}{4}\sup _{0\le p_2<\epsilon }\int _{4p_2-4p_2^2}^{2\epsilon } {\text {d}}z \Bigg [\left( \int _{0}^{4\epsilon ^2} \ln \left( \frac{18}{x} \right) ^6 {\text {d}}x \right) ^{1/3}\left( \int _{0}^{4\epsilon ^2} \frac{1}{y^{3/4}}{\text {d}}y\right) ^{2/3}\Bigg ] \\  &\le \frac{C}{2} \epsilon \left( \int _{0}^{4\epsilon ^2} \ln \left( \frac{18}{x} \right) ^6 {\text {d}}x \right) ^{1/3}\left( \int _{0}^{4\epsilon ^2} \frac{1}{y^{3/4}}{\text {d}}y\right) ^{2/3}, \end{aligned}\end{aligned}$$

which vanishes as $\epsilon \rightarrow 0$. For $I_9$ we bound (analogously to (7.86))

7.90 $$\begin{aligned} \begin{aligned}&\int _{-\sqrt{\mu _2}+p_2}^{\sqrt{\mu _1}-p_2} \frac{1}{1-p_1^2-q_1^2-p_2^2-q_2^2} {\text {d}}q_2 \\  &\quad \le 2 \int _{0}^{\sqrt{\mu _2}-p_2} \frac{1}{1-p_1^2-q_1^2-p_2^2-q_2^2}{\text {d}}q_2 \\  &\quad =\frac{1}{\sqrt{1-p_1^2-q_1^2-p_2^2}}\ln \left( \frac{\big (\sqrt{1-p_1^2-q_1^2-p_2^2}+\sqrt{\mu _2}-p_2\big )^2}{2(p_2(\sqrt{\mu _2}-p_2)-p_1 q_1)} \right) \\  &\quad \le \frac{1}{\sqrt{1-3\epsilon ^2}}\ln \left( \frac{4}{2(p_2(\sqrt{\mu _2}-p_2)-p_1 q_1)} \right) . \end{aligned} \end{aligned}$$

Substituting $z=p_1+q_1$ and $r=p_1-q_1$ we obtain

7.91 $$\begin{aligned} \begin{aligned} I_9\le&C \sup _{0\le p_2<\epsilon }\int _{{\mathbb {R}}^2} \chi _{0<p_1,q_1<\epsilon }\chi _{2p_2>\sqrt{\mu _2}-\sqrt{\mu _1}} \\  &\times \ln \left( \frac{4}{2(p_2(\sqrt{\mu _2}-p_2)-p_1 q_1)} \right) ^2 {\text {d}}p_1 {\text {d}}q_1 \\\le&\frac{C}{2}\sup _{0\le p_2<\epsilon }\int _{-\epsilon }^\epsilon {\text {d}}r \int _{0}^{2\epsilon } {\text {d}}z \chi _{|r|<z}\chi _{2p_2>\sqrt{1-r^2}-\sqrt{1-z^2}} \\  &\times \ln \left( \frac{8}{4p_2(\sqrt{1-r^2}-p_2) -z^2+r^2} \right) ^2. \end{aligned}\end{aligned}$$

Substituting *z* by $x=4p_2(\sqrt{1-r^2}-p_2) -z^2+r^2$ and using Hölder’s inequality, we obtain

7.92 $$\begin{aligned} I_9\le &   \frac{C}{4}\sup _{0\le p_2<\epsilon }\int _{-\epsilon }^\epsilon {\textrm{d}}r \int _{0}^{4p_2(\sqrt{1-r^2}-p_2)+r^2} \ln \left( \frac{8}{x} \right) ^2 \nonumber \\  &   \times \frac{1}{\sqrt{4p_2(\sqrt{1-r^2}-p_2) +r^2-x}} {\textrm{d}}x \nonumber \\\le &   \frac{C}{4}\sup _{0\le p_2<\epsilon }\int _{-\epsilon }^\epsilon {\textrm{d}}r \left( \int _{0}^{4p_2(\sqrt{1-r^2}-p_2)+r^2} \ln \left( \frac{8}{x} \right) ^6 {\textrm{d}}x\right) ^{1/3}\nonumber \\  &   \times \left( \int _{0}^{4p_2(\sqrt{1-r^2}-p_2)+r^2} \frac{1}{(4p_2(\sqrt{1-r^2}-p_2) +r^2-x)^{3/4}} {\textrm{d}}x\right) ^{2/3} \nonumber \\\le &   \frac{C}{2} \epsilon \left( \int _{0}^{4\epsilon +\epsilon ^2} \ln \left( \frac{8}{x} \right) ^6 {\textrm{d}}x\right) ^{1/3}\left( \int _{0}^{4\epsilon +\epsilon ^2} \frac{1}{y^{3/4}} {\textrm{d}}y\right) ^{2/3}, \end{aligned}$$

which vanishes for $\epsilon \rightarrow 0$. $\square$

### Proof of Lemma 6.9

#### Proof of Lemma 6.9

To prove Lemma 6.9 we show that the following expressions are finite:

(i) $$\sup _{T>\mu /2} \sup _{ q \in {\mathbb {R}}^d} \Vert V^{1/2} B_{T}(\cdot , q) |V |^{1/2}\Vert $$
(ii) $$\sup _T \sup _{ q \in {\mathbb {R}}^d} \Vert V^{1/2} B_{T}(\cdot , q) \chi _{|\cdot |^2>3\mu } |V |^{1/2}\Vert $$
(iii) $$\sup _T \sup _{q\in {\mathbb {R}}^d} \Vert V^{1/2} B_{T}(\cdot , q) \chi _{((\cdot +q)^2-\mu )((\cdot -q)^2-\mu )<0} |V |^{1/2}\Vert $$
(iv) $$\sup _T \sup _{\vert q \vert>\frac{\sqrt{\mu }}{2}} \Vert V^{1/2} B_{T}(\cdot , q) \chi _{p^2<3\mu } \chi _{((\cdot +q)^2-\mu )((\cdot -q)^2-\mu )>0} |V |^{1/2}\Vert $$
(v) $$\sup _T \sup _{\vert q \vert<\frac{\sqrt{\mu }}{2}} \left\| V^{1/2} \left[ B_{T}(\cdot , q) \chi _{|\cdot |^2<3\mu } \chi _{((\cdot +q)^2-\mu )((\cdot -q)^2-\mu )>0}-Q_{T}(q) \right] \right. \left. \times |V |^{1/2}\right\| $$

In combination, they prove (6.33).

We start with (i) and (ii). By Lemma 2.1 there is a constant $c_0$ depending only on $\mu$, such that $B_T(p,q)\le c_0/(1+p^2)$ for all $T>\mu /2$ and $p,q\in {\mathbb {R}}^d$. Similarly, using (2.3) one sees that there is a constant $c_1$ depending only on $\mu$ such that $B_T(p,q)\le c_1/(1+p^2)$ for all $T>0$ and $p,q\in {\mathbb {R}}^d$ with $q^2>3\mu$. The claim follows since $\Vert |V|^{1/2}\frac{1}{1-\Delta } |V |^{1/2}\Vert$ is bounded [8, 9, 11].

For (iii), it suffices to prove that

7.93 $$\begin{aligned} Y= \sup _{T} \sup _{q\in {\mathbb {R}}^d} \int _{{\mathbb {R}}^d} B_{T}(p, q)\chi _{((p+q)^2-\mu )((p-q)^2-\mu )<0} {\textrm{d}}p <\infty , \end{aligned}$$

since (iii) is bounded by $\Vert V \Vert _1 Y$. The integrand is invariant under rotation of $(p,q)\rightarrow (R p, Rq)$ around the origin. Hence, the integral only depends on the absolute value of *q* and we may take the supremum over *q* of the form $q=(\vert q \vert ,0)$ only. For $p,(q_1,0)$ satisfying $((p+(q_1,0))^2-\mu )((p-( q_1,0))^2-\mu )<0$, we can estimate that by [6, Lemma 4.7]

7.94 $$\begin{aligned}&B_{T}(p,(q_1,0))\nonumber \\&\quad \le \frac{2}{T} \exp \left( -\frac{1}{T}\min \{(\vert p_1\vert + \vert q_1 \vert )^2+{\tilde{p}}^2-\mu , \mu -(\vert p_1\vert - \vert q_1 \vert )^2-{\tilde{p}}^2 \}\right) . \end{aligned}$$

Note that $(\vert p_1\vert + \vert q_1 \vert )^2+{\tilde{p}}^2-\mu<\mu -(\vert p_1\vert - \vert q_1 \vert )^2-{\tilde{p}}^2 \leftrightarrow p^2+q_1^2<\mu$. We can therefore further estimate

7.95 $$\begin{aligned}  &   B_{T}(p,(q_1,0)) \chi _{(\vert p_1\vert + \vert q_1 \vert )^2+{\tilde{p}}^2>\mu>(\vert p_1\vert - \vert q_1 \vert )^2+{\tilde{p}}^2} \nonumber \\  &   \quad \le \frac{2}{T} \exp \left( -\frac{1}{T}((\vert p_1\vert + \vert q_1 \vert )^2+{\tilde{p}}^2-\mu )\right) \chi _{(\vert p_1\vert + \vert q_1 \vert )^2+{\tilde{p}}^2>\mu } \chi _{p^2+q_1^2<\mu }\nonumber \\  &   \qquad +\frac{2}{T} \exp \left( -\frac{1}{T}(\mu -(\vert p_1\vert - \vert q_1 \vert )^2-{\tilde{p}}^2)\right) \chi _{\mu >(\vert p_1\vert - \vert q_1 \vert )^2+{\tilde{p}}^2}. \end{aligned}$$

We now integrate the bound over *p* and use the symmetry in $p_1$ to restrict to $p_1>0$, replace $\vert p_1\vert$ by $p_1$ and then extend the domain to $p_1\in {\mathbb {R}}$. We obtain

7.96 $$\begin{aligned} Y\le &   \sup _{T} \sup _{q_1\in {\mathbb {R}}} \frac{4}{T} \left[ \int _{{\mathbb {R}}^d} \exp \left( -\frac{1}{T}(( p_1 + \vert q_1 \vert )^2+{\tilde{p}}^2-\mu )\right) \right. \nonumber \\  &   \times \left. \chi _{( p_1 + \vert q_1 \vert )^2+{\tilde{p}}^2>\mu } \chi _{p^2+q_1^2<\mu }{\textrm{d}}p \right. \nonumber \\  &   +\left. \int _{{\mathbb {R}}^d} \exp \left( -\frac{1}{T}(\mu -( p_1 - \vert q_1 \vert )^2-{\tilde{p}}^2)\right) \chi _{\mu >( p_1 - \vert q_1 \vert )^2+{\tilde{p}}^2} {\textrm{d}}p\right] .\quad \end{aligned}$$

Now we substitute $p_1 \pm \vert q_1 \vert$ by $p_1$ and obtain

7.97 $$\begin{aligned} Y&\le \sup _{T} \sup _{\vert q_1\vert<\sqrt{\mu }}\frac{4}{T} \int _{{\mathbb {R}}^d} \exp \left( -\frac{1}{T}(p_1^2+{\tilde{p}}^2-\mu )\right) \chi _{p_1^2+{\tilde{p}}^2>\mu } \chi _{(p_1-\vert q_1 \vert )^2+{\tilde{p}}^2+q_1^2<\mu }{\textrm{d}}p\nonumber \\&\quad +\sup _{T}\frac{4}{T} \int _{{\mathbb {R}}^d} \exp \left( -\frac{1}{T}(\mu - p_1^2-{\tilde{p}}^2)\right) \chi _{\mu >p_1^2+{\tilde{p}}^2} {\textrm{d}}p\nonumber \\&\le \sup _{T} \frac{4 \vert {\mathbb {S}}^{d-1}\vert (2\sqrt{\mu })^{d-1}e^{\mu /T}}{T} \int _{\sqrt{\mu }}^{\infty } e^{-r^2/T} {\textrm{d}}r\nonumber \\&\quad +\sup _{T}\frac{4 \vert {\mathbb {S}}^{d-1}\vert \sqrt{\mu }^{d-1}e^{-\mu /T}}{T} \int _{0}^{\sqrt{\mu }} e^{r^2/T} {\textrm{d}}r, \end{aligned}$$

where we used that $(p_1-\vert q_1 \vert )^2+{\tilde{p}}^2+q_1^2<\mu \Rightarrow p^2<2\mu$. Note that

7.98 $$\begin{aligned} \frac{ \sqrt{\mu }e^{\mu /T}}{T} \int _{\sqrt{\mu }}^{\infty } e^{-r^2/T} {\textrm{d}}r= \frac{\pi ^{1/2}}{2} \sqrt{\frac{\mu }{T}}e^{\mu /T} \text {erfc}\left( \sqrt{\frac{\mu }{T}}\right) \end{aligned}$$

and

7.99 $$\begin{aligned} \frac{ \sqrt{\mu }e^{-\mu /T}}{T} \int _{0}^{\sqrt{\mu }} e^{r^2/T} {\textrm{d}}r= \frac{\pi ^{1/2}}{2} \sqrt{\frac{\mu }{T}}e^{-\mu /T} \text {erfi}\left( \sqrt{\frac{\mu }{T}}\right) . \end{aligned}$$

As in the proof of [6, Lemma 4.4], we conclude that $Y<\infty$ since the functions $xe^{x^2} \text {erfc}(x)$ and $xe^{-x^2} \text {erfi}(x)$ are bounded for $x\ge 0$.

For (iv), it again suffices to prove that

7.100 $$\begin{aligned} X=\sup _T \sup _{\vert q \vert> \frac{\sqrt{\mu }}{2}}\int _{{\mathbb {R}}^d} B_{T}(p,q) \chi _{p^2<3\mu } \chi _{((p+q)^2-\mu )((p-q)^2-\mu )>0} {\textrm{d}}p <\infty ,\nonumber \\ \end{aligned}$$

since (iv) is bounded by $\Vert V \Vert _1 X$. Again we can restrict to *q* of the form $q=(\vert q \vert ,0)$. The idea is to split the integrand in *X* into four terms localized in different regions. The integrand is supported on the intersection and the complement of the two disks/balls with radius $\sqrt{\mu }$ centered at $(\pm q_1,0)$. (For $d=2$ this is the white region in Fig. 3).

- The first term covers the domain with $\vert {\tilde{p}} \vert > \sqrt{\mu }$ outside the disks/balls: $X_1=\sup _T \sup _{\vert q_1 \vert> \frac{\sqrt{\mu }}{2}}\int _{{\mathbb {R}}^d} B_{T}(p,(q_1,0)) \chi _{p^2<3\mu } \chi _{ {\tilde{p}}^2 >\mu } {\textrm{d}}p$
- The second term covers the remaining domain with $\vert p_1 \vert > \vert q_1 \vert$ outside the two disks/balls:
  $$\begin{aligned} X_2=\sup _T \sup _{\vert q_1 \vert> \frac{\sqrt{\mu }}{2}} \int _{{\tilde{p}}^2<\mu } {\textrm{d}}{\tilde{p}} \int _{\vert p_1 \vert >\sqrt{\mu -{\tilde{p}}^2}+\vert q_1 \vert } {\textrm{d}}p_1 B_{T}(p,(q_1,0)) \chi _{p^2<3\mu } \end{aligned}$$
- The third term covers the remaining domain with $\vert p_1 \vert < \vert q_1 \vert$ outside the two disks/balls:
  $$\begin{aligned} X_3=\sup _T \sup _{\vert q_1 \vert > \frac{\sqrt{\mu }}{2}} \int _{\mu -q_1^2<{\tilde{p}}^2<\mu } {\textrm{d}}{\tilde{p}} \int _{\vert p_1 \vert<-\sqrt{\mu -{\tilde{p}}^2}+\vert q_1 \vert } {\textrm{d}}p_1 B_{T}(p,(q_1,0)) \chi _{p^2<3\mu } \end{aligned}$$
- The fourth term covers the domain in the intersection of the two disks/balls:
  $$\begin{aligned} X_4=\sup _T \sup _{\vert q_1 \vert > \frac{\sqrt{\mu }}{2}} \int _{{\tilde{p}}^2<\mu -q_1^2} {\text {d}}{\tilde{p}} \int _{\vert p_1 \vert<\sqrt{\mu -{\tilde{p}}^2}-\vert q_1 \vert } {\text {d}}p_1 B_{T}(p,(q_1,0)) \chi _{p^2<3\mu } \end{aligned}$$

We prove that each $X_j$ is finite. It then follows that $X \le X_1+X_2+X_3+X_4$ is finite. We use the bounds

7.101 $$\begin{aligned} B_{T}(p,(q_1,0))\le \left\{ \begin{array}{cc} \frac{1}{p^2+q_1^2-\mu } &  \text {if}\ (\vert p_1\vert - \vert q_1 \vert )^2+{\tilde{p}}^2 >\mu , \\ \frac{1}{\mu - p^2-q_1^2} &  \text {if}\ (\vert p_1\vert +\vert q_1 \vert )^2+{\tilde{p}}^2<\mu , \end{array}\right. \end{aligned}$$

which follow from (6.18). The first line applies to $X_1,X_2, X_3$, the second line to $X_4$. For $X_1$, we have $p^2+q_1^2-\mu>q_1^2>\mu /4$ and thus $X_1<\infty$. Similarly, for $X_2$, we have $p^2+q_1^2-\mu = (\sqrt{ q_1^2+p_1^2} +\sqrt{\mu -{\tilde{p}}^2})(\sqrt{ q_1^2+p_1^2} -\sqrt{\mu -{\tilde{p}}^2}) \ge \vert q_1\vert (\vert p_1\vert -\sqrt{\mu -{\tilde{p}}^2})\ge q_1^2\ge \mu /4$ and thus $X_2<\infty$. For $X_3$, we have $p^2+q_1^2-\mu \ge \vert q_1\vert (\vert q_1\vert -\sqrt{\mu -{\tilde{p}}^2})\ge \frac{\sqrt{\mu }}{2}(\vert q_1\vert -\sqrt{\mu -{\tilde{p}}^2})$. Hence, $X_3 \le \sup _{\vert q_1 \vert > \frac{\sqrt{\mu }}{2}}\frac{4}{\sqrt{\mu }} \int _{\mu -q_1^2<{\tilde{p}}^2<\mu } {\textrm{d}}{\tilde{p}} <\infty$. For $X_4$ we have $\mu -p^2-q_1^2\ge \mu -(\sqrt{\mu -{\tilde{p}}^2}-\vert q_1 \vert )^2- {\tilde{p}}^2-q_1^2 =2 \vert q_1 \vert (\sqrt{\mu -{\tilde{p}}^2}-|q_1|)$. Thus,

7.102 $$\begin{aligned} X_4\le \sup _{\vert q_1 \vert > \frac{\sqrt{\mu }}{2}} \int _{{\tilde{p}}^2<\mu -q_1^2} \frac{1}{|q_1|} {\textrm{d}}{\tilde{p}}<\infty . \end{aligned}$$

To prove that (v) is finite, let $S_{T,d}(q):L^1({\mathbb {R}}^d)\rightarrow L^\infty ({\mathbb {R}}^d)$ be the operator with integral kernel

7.103 $$\begin{aligned} S_{T,d}(q)(x,y)= &   \frac{1}{(2\pi )^d} \int _{{\mathbb {R}}^d} \left[ e^{i (x-y)\cdot p } -e^{i \sqrt{\mu } (x-y)\cdot p/\vert p \vert } \right] \nonumber \\  &   \times B_{T}(p,q) \chi _{((p+q)^2-\mu )((p-q)^2-\mu )>0} \chi _{p^2<3\mu } {\textrm{d}}p. \end{aligned}$$

Then v equals $\sup _T \sup _{\vert q \vert <\frac{\sqrt{\mu }}{2}} \left\Vert V^{1/2} S_{T,d}(q) |V |^{1/2}\right\Vert$. With (2.3) and $\vert e^{i x}- e^{i y}\vert \le \min \left\{ \vert x-y\vert ,2\right\}$ we obtain

7.104 $$\begin{aligned} \vert S_{T,d}(q)(x,y) \vert\le &   \frac{1}{(2\pi )^d} \int _{{\mathbb {R}}^d} \frac{\min \left\{ \vert (\vert p \vert -\sqrt{\mu }) (x-y) \cdot p/\vert p \vert \vert ,2\right\} }{\vert p^2+q^2-\mu \vert } \nonumber \\  &   \times \chi _{((p+q)^2-\mu )((p-q)^2-\mu )>0} \chi _{p^2<3\mu } {\textrm{d}}p\nonumber \\\le &   \frac{1}{(2\pi )^d} \int _{{\mathbb {R}}^d} \frac{\min \left\{ \vert \vert p\vert -\sqrt{\mu }\vert \vert x-y \vert ,2\right\} }{\vert p^2+q^2-\mu \vert } \nonumber \\  &   \times \chi _{((p+q)^2-\mu )((p-q)^2-\mu )>0} \chi _{p^2<3\mu } {\textrm{d}}p. \end{aligned}$$

Again, the integral only depends on $\vert q \vert$, so we may restrict to $q=(\vert q \vert ,0)$. We now switch to angular coordinates. Recall the notation $r_\pm$ and $e_\varphi$ introduced before (6.37) and that $(\vert r \cos \varphi \vert \mp \vert q_1 \vert )^2+r^2 \sin \varphi ^2\gtrless \mu \leftrightarrow r\gtrless r_\pm (e_\varphi )$. For $d=2$ we have

7.105 $$\begin{aligned} \vert S_{T,2}((q_1,0))(x,y) \vert \le \frac{1}{(2\pi )^2} \int _0^{2\pi } \Bigg [\int _{r_+(e_\varphi )}^{\sqrt{3\mu }} \frac{\min \left\{ \vert r-\sqrt{\mu } \vert \vert x-y \vert ,2\right\} }{r^2+q_1^2-\mu }r{\textrm{d}}r\nonumber \\ + \int _{0}^{r_-(e_\varphi )} \frac{\min \left\{ (\sqrt{\mu }-r) \vert x-y \vert ,2\right\} }{\mu -r^2-q_1^2} r{\textrm{d}}r\Bigg ] {\textrm{d}}\varphi =: g(x,y,q_1)\nonumber \\ \end{aligned}$$

and for $$d=3$$

7.106 $$\begin{aligned} \vert S_{T,3}((q_1,0))(x,y) \vert \le \frac{1}{(2\pi )^2} \int _0^{\pi } \Bigg [\int _{r_+(e_\theta )}^{\sqrt{3\mu }} \frac{\min \left\{ \vert r-\sqrt{\mu } \vert \vert x-y \vert ,2\right\} }{r^2+q_1^2-\mu }\sin \theta r^2{\textrm{d}}r\nonumber \\ + \int _{0}^{r_-(e_\theta )} \frac{\min \left\{ (\sqrt{\mu }-r) \vert x-y \vert ,2\right\} }{\mu -r^2-q_1^2} \sin \theta r^2{\textrm{d}}r\Bigg ] {\textrm{d}}\theta \le \frac{\sqrt{3\mu }}{2}g(x,y,q_1).\nonumber \\ \end{aligned}$$

We bound *g* by

7.107 $$\begin{aligned} \begin{aligned}&\vert g(x,y,q_1) \vert \\  &\quad \le \frac{1}{(2\pi )^2} \int _0^{2\pi } \left[ \int _{r_+(e_\varphi )}^{\sqrt{3\mu }} \frac{\min \left\{ (r-r_+(e_\varphi ) ) \vert x-y \vert ,2\right\} +\min \left\{ \vert \sqrt{\mu }- r_+(e_\varphi )\vert \vert x-y \vert ,2\right\} }{r^2+q_1^2-\mu } r {\text {d}}r \right. \\  &\qquad \left. + \int _{0}^{r_-(e_\varphi )} \frac{\min \left\{ (r_-(e_\varphi )-r) \vert x-y \vert ,2\right\} +\min \left\{ (\sqrt{\mu }-r_-(e_\varphi ))\vert x-y \vert ,2\right\} }{\mu -r^2-q_1^2} r {\text {d}}r \right] {\text {d}}\varphi . \end{aligned} \end{aligned}$$

Note that $r_+(e_\varphi )$ attains the minimal value $\sqrt{\mu -q_1^2}$ at $\vert \varphi \vert =\frac{\pi }{2}$ and the maximal value $\sqrt{\mu }+\vert q_1 \vert$ at $\vert \varphi \vert =0$. Similarly, $r_-(e_\varphi )$ attains the maximal value $\sqrt{\mu -q_1^2}$ at $\vert \varphi \vert =\frac{\pi }{2}$ and the minimal value $\sqrt{\mu }-\vert q_1 \vert$ at $\vert \varphi \vert =0$. For the first summand in both integrals we take the supremum over the angular variable. For the second summand in both integrals, we carry out the integration over *r* and use that $\vert \sqrt{\mu }-r_-(e_\varphi )\vert , \vert \sqrt{\mu }- r_+(e_\varphi )\vert \le \vert q_1 \vert$. We obtain the bound

7.108 $$\begin{aligned}&\vert g(x,y,q_1) \vert \nonumber \\&\quad \le \frac{1}{2\pi } \int _{0}^{\sqrt{3\mu }} \frac{\min \left\{ \vert r-\sqrt{\mu -q_1^2} \vert \vert x-y \vert ,2\right\} r}{\vert r^2+q_1^2-\mu \vert }{\textrm{d}}r +\frac{\min \left\{ \vert q_1 \vert \vert x-y \vert ,2\right\} }{2(2\pi )^2} \nonumber \\&\qquad \times \int _0^{2\pi } \left[ \ln \left( \frac{2\mu +q_1^2}{r_+(e_\varphi )^2+q_1^2-\mu }\right) +\ln \left( \frac{\mu -q_1^2}{\mu -q_1^2 -r_-(e_\varphi )^2}\right) \right] {\textrm{d}}\varphi . \end{aligned}$$

Recall that we are only interested in $\vert q_1 \vert <\sqrt{\mu }/2$. For the first term, we use that $r\le \sqrt{3\mu }$ and $\vert r^2+q_1^2-\mu \vert = \vert r-\sqrt{\mu -q_1^2} \vert \vert r+\sqrt{\mu -q_1^2} \vert \ge \vert r-\sqrt{\mu -q_1^2} \vert \sqrt{\mu -q_1^2}$. This gives the following bound, where we first carry out the *r*-integration and then use that $\sqrt{\mu -q_1^2}\ge \sqrt{3\mu }/2$:

7.109 $$\begin{aligned}  &   \frac{\sqrt{3\mu }}{\pi \sqrt{\mu -q_1^2}} \int _{0}^{\sqrt{3\mu }}\min \left\{ \frac{\vert x-y \vert }{2}, \frac{1}{\vert r-\sqrt{\mu -q_1^2}\vert }\right\} {\textrm{d}}r\nonumber \\  &   \quad \le \frac{\sqrt{3\mu }}{\pi \sqrt{\mu -q_1^2}} \left[ \ln \left( \max \left\{ 1, \frac{\sqrt{\mu -q_1^2}\vert x-y\vert }{2}\right\} \right) \right. \nonumber \\  &   \qquad \left. +2+\ln \left( \max \left\{ 1, \frac{(\sqrt{3\mu }-\sqrt{\mu -q_1^2})\vert x-y \vert }{2}\right\} \right) \right] \nonumber \\  &   \quad \le C \left[ 1+\ln \left( 1+ \frac{\sqrt{3\mu }\vert x-y \vert }{2}\right) \right] . \end{aligned}$$

For the second term, we use that

7.110 $$\begin{aligned} \frac{2\mu +q_1^2}{r_+(e_\varphi )^2+q_1^2-\mu }\frac{\mu -q_1^2}{\mu -q_1^2 -r_-(e_\varphi )^2}= \frac{2\mu +q_1^2}{4 \vert e_{\varphi ,1} \vert ^2 \vert q_1 \vert ^2} \end{aligned}$$

and $\vert q_1 \vert <\sqrt{\mu }/2$, as well as $\vert e_{\varphi ,1}\vert =\vert \cos \varphi \vert \ge \frac{1}{2}\min \{\vert \frac{\pi }{2}-\varphi \vert ,\vert \frac{3\pi }{2}-\varphi \vert \}$, to arrive at the bound

7.111 $$\begin{aligned}  &   \frac{\min \left\{ \vert q_1 \vert \vert x-y \vert ,2\right\} }{(2\pi )^2} \int _0^{2\pi } \ln \left( \frac{\sqrt{3\mu }}{2\vert e_{\varphi ,1} q_1\vert }\right) {\textrm{d}}\varphi \nonumber \\  &   \quad \le \frac{4 \min \left\{ \vert q_1 \vert \vert x-y \vert ,2\right\} }{(2\pi )^2} \int _0^{\pi /2} \ln \left( \frac{\sqrt{3\mu }}{\vert \varphi q_1\vert }\right) {\textrm{d}}\varphi \nonumber \\  &   \quad =\frac{\min \left\{ \vert q_1 \vert \vert x-y \vert ,2\right\} }{2\pi } \left( 1+\ln \left( \frac{2\sqrt{3\mu }}{\pi \vert q_1\vert }\right) \right) \nonumber \\  &   \quad =\frac{\min \left\{ \vert q_1 \vert \vert x-y \vert ,2\right\} }{2\pi } \left( 1+\ln \left( \sqrt{3\mu }\vert x-y \vert \right) +\ln \left( \frac{2\pi }{\vert x- y \vert \vert q_1\vert }\right) \right) ,\nonumber \\ \end{aligned}$$

where we used $\int \ln (1/x) {\textrm{d}}x =x+ x \ln (1/x)$. Since $x \ln (1/x) \le C$, this is bounded above by

7.112 $$\begin{aligned} \frac{1}{\pi } \left( 1+\max \left\{ \ln \left( \sqrt{3\mu }\vert x-y \vert \right) ,0\right\} \right) +C. \end{aligned}$$

In total, we obtain the bound

7.113 $$\begin{aligned} \sup _{\vert q_1 \vert <\frac{\sqrt{\mu }}{2}} \vert g(x,y,q_1) \vert \le C\left[ 1+\ln \left( 1+ \sqrt{\mu }\vert x-y \vert \right) \right] . \end{aligned}$$

Let $M:L^2({\mathbb {R}}^d)\rightarrow L^2({\mathbb {R}}^d)$ be the operator with integral kernel $M(x,y)=|V|^{1/2} (x) (1+\ln \left( 1+\sqrt{\mu }\vert x-y \vert \right) ) |V|^{1/2}(y)$. We have

7.114 $$\begin{aligned} \sup _T \sup _{\vert q \vert <\frac{\sqrt{\mu }}{2}} \left\Vert V^{1/2} S_{T,d}(q) |V|^{1/2}\right\Vert \le C(\mu ,d) \Vert M \Vert \end{aligned}$$

for some constant $C(\mu ,d)<\infty$. The operator *M* is Hilbert–Schmidt, since the function $x\mapsto (1+\ln (1+\vert x\vert )^2)|V(x)|$ is in $L^1({\mathbb {R}}^d).$ $\square$

## Data Availability

Data sharing not applicable to this article as no datasets were generated or analysed during the current study.
